# Discovery of Azaindolin-2-One as a Dual Inhibitor of GSK3β and Tau Aggregation with Potential Neuroprotective Activity

**DOI:** 10.3390/ph15040426

**Published:** 2022-03-31

**Authors:** Taha F. S. Ali, Halil I. Ciftci, Mohamed O. Radwan, Eslam Roshdy, Ahmed M. Shawky, Mohammed A. S. Abourehab, Hiroshi Tateishi, Masami Otsuka, Mikako Fujita

**Affiliations:** 1 Medicinal Chemistry Department, Faculty of Pharmacy, Minia University, Minia 61519, Egypt; m211749@hiroshima-u.ac.jp; 2 Medicinal and Biological Chemistry Science Farm Joint Research Laboratory, Faculty of Life Sciences, Kumamoto University, Kumamoto 862-0973, Japan; hiciftci@kumamoto-u.ac.jp (H.I.C.); mohamedradwan@kumamoto-u.ac.jp (M.O.R.); htateishi@kumamoto-u.ac.jp (H.T.); motsuka@gpo.kumamoto-u.ac.jp (M.O.); 3 Department of Drug Discovery, Science Farm, Ltd., Kumamoto 862-0976, Japan; 4 National Research Centre, Chemistry of Natural Compounds Department, Pharmaceutical and Drug Industries Research Division, Dokki, Cairo 12622, Egypt; 5 Department of Chemistry, Graduate School of Science, Hiroshima University, Higashi-Hiroshima, Hiroshima 739-8526, Japan; 6 Science and Technology Unit (STU), Umm Al-Qura University, Makkah 21955, Saudi Arabia; amesmail@uqu.edu.sa; 7 Central Laboratory for Micro-Analysis, Minia University, Minia 61519, Egypt; 8 Department of Pharmaceutics, Faculty of Pharmacy, Umm Al-Qura University, Makkah 21955, Saudi Arabia; maabourehab@uqu.edu.sa

**Keywords:** azaindolin-2-one, GSK3β, Tau, protein aggregation, neurofibrillary tangles, neuroprotective, Alzheimer’s disease

## Abstract

The inhibition of glycogen synthase kinase 3β (GSK3β) activity through pharmacological intervention represents a promising approach for treating challenging neurodegenerative disorders like Alzheimer’s disease. Similarly, abnormal tau aggregate accumulation in neurons is a hallmark of various neurodegenerative diseases. We introduced new dual GSK3β/tau aggregation inhibitors due to the excellent clinical outcome of multitarget drugs. Compound **(*E*)-2f** stands out among the synthesized inhibitors as a promising GSK3β inhibitor (IC_50_ 1.7 µM) with a pronounced tau anti-aggregation effect in a cell-based model of tauopathy. Concurrently, **(*E*)-2f** was demonstrated to be non-toxic to normal cells, making it a promising neuroprotective lead compound that needs further investigation.

## 1. Introduction

Alzheimer’s disease (AD) is a neurological disorder manifested with memory loss as well as cognitive and linguistic impairment [1]. Despite several theoretical ideas and models, there is no complete explanation for AD etiology, which has become a critical constraint in developing innovative AD therapies. However, AD has two neuropathological characteristics: the β-amyloid plaques and the neurofibrillary tangles (NFTs), both of which are prevalent in the hippocampus region [2,3]. When these two conditions are combined, cholinergic neurons are gradually lost, resulting in cognitive impairment.

According to the Amyloid Cascade Theory, AD amyloid-β (Aβ) deposition in the brain is the first event. However, there is mounting evidence that this explanation falls short of explaining many aspects of AD pathogenesis. Increased inflammatory markers in Alzheimer’s patients and identifying AD risk genes linked to innate immune processes denote that neuroinflammation plays a crucial role in AD’s etiology [4]. Glial cells, including astrocytes, microglia, NG2 glia, and oligodendrocytes, may play a significant role in the pathophysiology of AD. Astrocytes and microglia are critical for the course and outcome of AD, either because they act as effector cells, producing neuroprotective cytokines, or failing to perform their homeostatic roles, exposing neurons to excitotoxicity and oxidative stress [5].

The blood–brain barrier (BBB) is another factor that contributes to the initiation and maintenance of chronic inflammation in Alzheimer’s disease. The BBB is part of the neurovascular unit (NVU), including glial cells, neurons, and pericytes. During Alzheimer’s disease, the NVU becomes dysfunctional, and each of its components may experience functional alterations that lead to neuronal damage and cognitive loss [6].

Many epidemiological studies correlate type 2 diabetes mellitus (DM2) with Alzheimer’s disease. Diabetic patients have a higher rate of cognitive deterioration and are more likely to develop dementia. Cognitive deficiencies in people with diabetes impact psychomotor efficiency, attention, learning and memory, mental flexibility, and executive function [7].

Indeed, the currently licensed medications for AD are not disease-modifying and only provide moderate and brief symptom relief. Since Alzheimer’s disease is multifactorial, traditional single-target therapy is ineffective for treatment. Consequently, there is no specific drug for AD, despite numerous studies [8]. Nevertheless, one of the most critical targets for AD is the “Glycogen synthase kinase 3β “(GSK3β), a serine/threonine phosphokinase that plays a crucial role in the pathophysiology of AD [1]. GSK3β has been extensively studied in the last decade, particularly concerning its role in neurological disorders. GSK3 activity is detected with high levels in the frontal lobe of AD patients, and its upregulated expression is distinguished in the post-synaptosome supernatants and hippocampus [9]. GSK3β directly regulates more than 40 proteins [10]. Metabolic/signaling proteins, structural proteins, and transcription factors are the three primary classes of GSK3β substrates [11,12]. Among the structural proteins identified as GSK3Bsubstrates, microtubule-associated proteins (MAPs), including Tau and MAP1B, are critical for neuronal polarity and axonal extension [13]. GSK3 activation phosphorylates the microtubule-associated protein tau, resulting in the formation of NFTs. Furthermore, pro-inflammatory mediators, microglia, astrocyte activation, and migration increase upon upregulation of GSK3β. All of these changes reduce neuronal–glial connections and exacerbate neuronal fragility [14,15,16,17].

Besides AD, many neurodegenerative diseases are marked by aberrant tau protein deposition in the brain. These tau-associated diseases are called Tauopathies. More than 20 disorders are associated with various tau pathologies, including globular glial tauopathies, chronic traumatic encephalopathy, Down syndrome, postencephalitic parkinsonism, neurofibrillary tangle dementia, and AD, among others [18].

Over the last decade, the multitarget drug discovery (MTDD) technique has raised hopes for drug development for multifactorial diseases such as AD [19]. These conditions are caused by distress in many linked networks, which the traditional single-target therapy cannot counter. The multitarget approach offers a pragmatic resolutive option for these diseases by providing chemotypes with several synergistic pharmacodynamic actions. A deliberate target combination must be pursued to achieve this goal. A multitarget drug must be a polypharmacological agent (interacts simultaneously with many targets), has more effectiveness than single targeted therapy, and has fewer adverse effects [20]. Multitarget drug (MTD) improves therapeutic efficacy or delays the emergence of resistance. Since the clinical studies required for an MTD are less sophisticated than those required for specific extensive therapy, it saves money, labor, and time [20].

**Figure 1:**
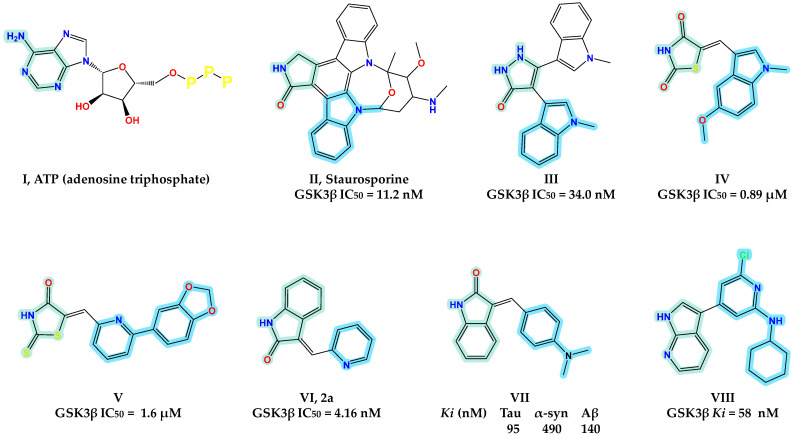
GSK3β and protein aggregation inhibitors.

The growing interest in GSK3 has resulted in numerous inhibitors with distinct scaffolds and different action mechanisms (e.g., ATP competition, allosteric modulation, and irreversible or covalent inhibition) [21,22]. Various natural and synthetic compounds have been studied as ATP-competitive GSK3β inhibitors over the last few decades (Figure 1). Natural compounds like Staurosporine (**II**) have been extensively investigated [23]. In 2011, Chen et al. repositioned compound **III** as an ATP-competitive GSK3β inhibitor rather than a protein kinase C inhibitor, as previously stated. In vitro, the bisindolyl-pyrazolone-based compound **III** demonstrated excellent GSK3β inhibitory action (IC_50_ = 34 nM) [24]. Gandini et al. identified compound **IV** as the first multitargeted scaffold that has the potential to inhibit GSK3β and tau-cascade [25].

Bourahla et al. developed compound **V** as a rhodamine-based GSK3β inhibitor with pyridyl substituent [26]. In 2019, Lozinskaya et al. developed a novel ATP competitive GSK3β inhibitor **VI** with a 3-pyridyl 2-oxindole ring as a scaffold [27]. Compound **VII,** reported by Chu et al., showed triple inhibitory actions at nanomolar concentrations. In vitro binding assays confirmed the critical efficiency of compound **VII** on alpha-synuclein (α-syn), beta-amyloid (Aβ), and tau fibrils [28]. Tong et al. developed an azaindole-pyridine-based GSK3β inhibitor (compound **VIII**) and identified the azaindole moiety as the hinge-binding motif [29]. Interestingly, Selenica M-L et al. demonstrated in vivo that six different classes of small-molecules GSK3β inhibitors effectively inhibit tau phosphorylation in regions relevant to the pathogenesis of AD and at brain concentrations within their IC50 range [30]. Therefore, the inhibition of GSK3β by small molecules is a promising strategy for therapeutic targeting of AD.

Since the MTDD technique is ideal for designing ligands for diseases with complicated pathology and lacks definitive treatment, we used the MTDD technique to design ligands bound to several targets, all associated with the AD etiology. Our ligands act as inhibitors for GSK3β as well as Aβ/tau/α-syn aggregation.

### Design of Dual GSK3β/tau Aggregation Inhibitors

It is challenging to design a ligand that can bind with two different targets with no homologous binding sites, such as GSK3β and tau protein [31]. More specifically, combining the molecular scaffolds of two central nervous system (CNS)-directed single-target ligands in a single small molecule may also be difficult, particularly in pharmacokinetics adjustment [32]. Many traditional GSK3 inhibitors have five-membered heterocyclic rings, such as 2-iminothiazolidin-4-one, hydantoin, or thiazolidinediones; these rings are essential to hydrogen bond acceptors and donors, which are critical to be active against GSK3β [33,34]. Instead, oxindole and its *N* bioisostere have attracted attention as new fragments with the same hydrogen bond functionalities. Compounds **VI** and **VIII** are typical examples of GSK3β inhibitors with oxindole (compound **VI**) or its *N* bioisostere azaindole (Compound **VIII**). The rationale for selecting these two nuclei is the extra ability to prevent the development of tau aggregates and destabilize preexisting tau aggregates in a dose-dependent manner.

**Figure 2:**
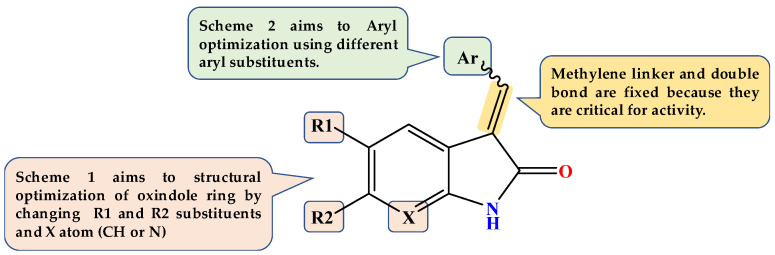
Design of our novel compounds as GSK3β inhibitors.

It was hypothesized that 5-arylidene substitution plays a vital role in the activity of 2-iminothiazolidin-4-one based GSK3β inhibitors. 5-Arylidene not only enhances GSK3β affinity but also enhances GSK3β selectivity as they do not fit in similar regions of homologous kinases [34]. This design would also allow the preservation of planarity and aromaticity, which are essential for tau fibril interaction [35]. Based on numerous reported examples of GSK3β inhibitors (Compounds **V**, **VI**, and **VIII**) and docking studies, we predicted that hybridizing pyridyl ring with oxindole or its *N* bioisostere would provide us with dual-acting anti-AD chemotypes, particularly as anti-tau and GSK3β inhibitors (Figure 2).

## 2. Results

We tested the following to determine if the synthesized compounds could hit both targets at the same time: (a) early GSK3β activity screening of all produced compounds, (b) Molecular docking study, (c) Selective cytotoxicity on cancer cells and normal blood cells, (d) Anti-tau aggregation in a tau pathology cell model.

### 2.1. Chemistry

**Figure 3:**
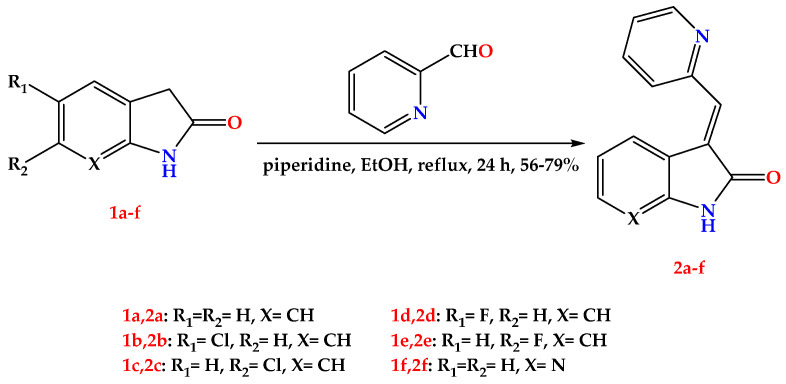
General procedure for the synthesis of compounds **2a–f**.

**Figure 4:**
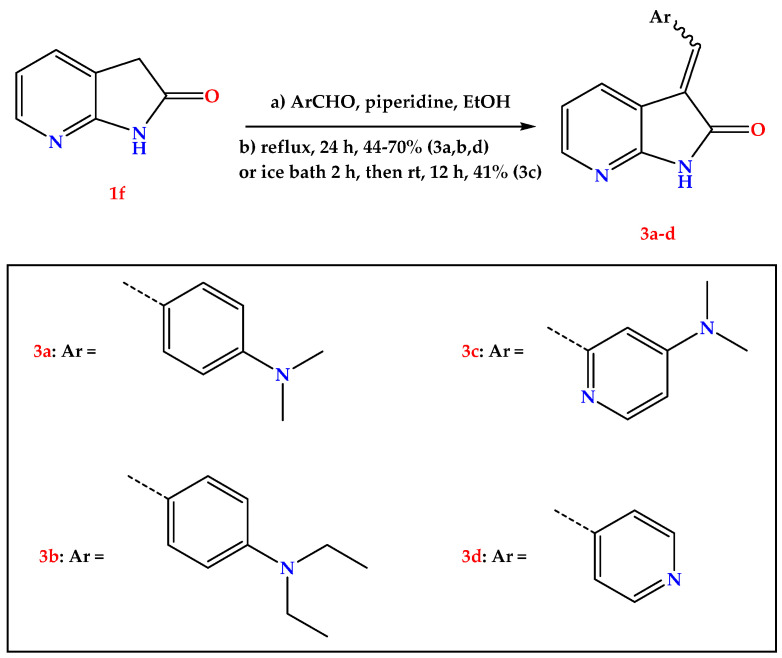
General procedure for the synthesis of compounds **3a–d**.

The target compounds were synthesized in two stages, as depicted in Figure 3 and Figure 4. In Figure 3, to structurally optimize the first part (oxindole or its pyrrolo [2,3-b] pyridine-2-one bioisostere), we have fixed the aryl substitution with the methylene linker as a 2-vinyl pyridine substituent on the indole ring. As demonstrated in Figure 3, we have synthesized six targeted molecules (**2a–f**) by 24 h refluxing of differently substituted oxindoles (**1a–f**) or 1,3-dihydro-2H-pyrrolo [2,3-b]pyridin-2-one with picolinaldehyde in the presence of ethanol as a solvent and piperidine as a catalyst.

To optimize the aryl part structurally, we kept the first part as 1,3-dihydro-2H-pyrrolo [2,3-b] pyridine-2-one, as depicted in Figure 4. The second series was synthesized by 24 h refluxing of 1,3-dihydro-2H-pyrrolo [2,3-b] pyridine-2-one with different aryl substituents in ethanol and piperidine to get three novel molecules (**3a,b** and **3d**). In comparison, compound **3c** was obtained without reflux by stirring in an ice bath for two hours then at room temperature for 12 h.

### 2.2. GSK3β Inhibition Assay

Screening of GSK3β inhibition activity for our new library was conducted using the Kinase-Glo luminescence assay to determine how much ATP is reduced after a kinase reaction [3]. All compounds were first tested at 10 μM and hit with a percentage inhibition of less than 50%, then were examined further to derive IC_50_ values, which were calculated using the linear regression parameter. Staurosporine (**II**) was used as a reference compound; it showed 98.46% inhibition at 10 μM. Excitingly, one of our first version compounds **(*E*)-2f** demonstrated 76.4% inhibition, while two compounds (**(*E*)-** and **(*E*)-2c**) showed percent inhibition at 45% and the others (**(*E*)-2d** and **(*E*)-2e**) showed less than 30%.

**Figure 5:**
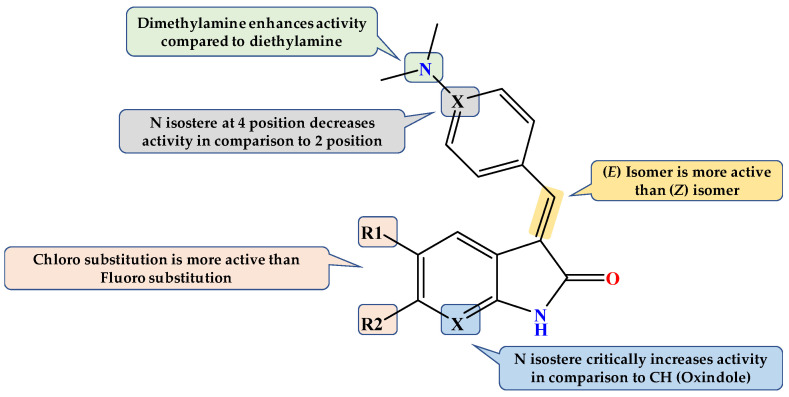
Structure-activity relationship (SAR) study of compounds **2a–f** and **3a–d** as novel GSK3β inhibitors.

As **(*E*)-2f** has the most potency, we tried to optimize structurally **(*E*)-2f** by using different pyridyl substituents, as shown in Figure 4. Upon measuring the IC_50_ of these compounds, none had IC_50_ values better than **(*E*)-2f,** which revealed an IC_50_ of 1.7 μM (Figure 5). However, the novel compounds had IC_50_ of no more than 3 μM, except **3b** and **3d,** as shown in Table 1.

### 2.3. Molecular Docking Study

In this study, the GSK3β protein structure (PDB ID: 4IQ6) was used because its co-crystallized ligand has azaindole chemotype, which is structurally similar to our azaindolin-2-one scaffold [29].

**Figure 6:**
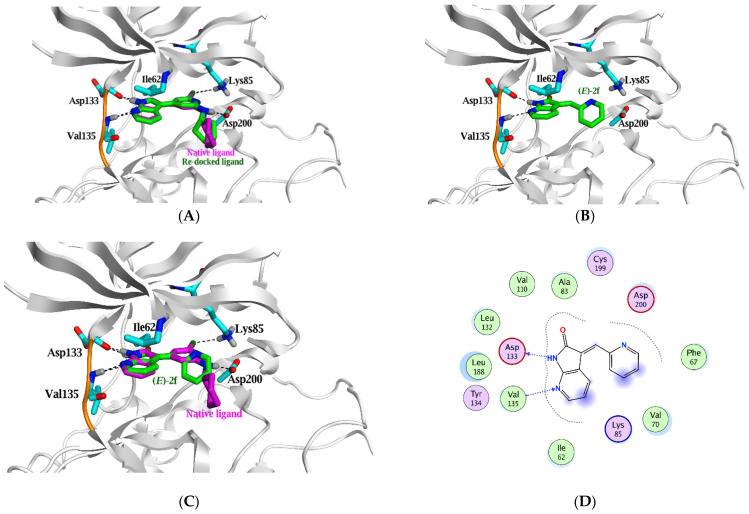

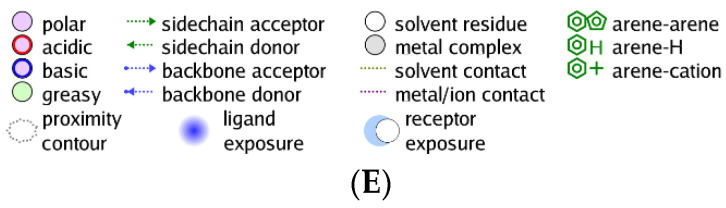
(**A**) Comparison between the binding mode of the native ligand (magenta stick model) and its superposed re-docked ligand (green stick model) within the active site of GSK3β (PDB ID: 4IQ6) predicted by Molecular Operating Environment (MOE) 2019.01. (**B**) The top-scoring docked pose of **(*E*)-2f**. (**C**) Comparison between the binding mode of the native ligand (magenta stick model) and **(*E*)-2f** (green stick model); hydrogen bonds are shown as black dashed lines, and the critical amino acid residues are displayed as a cyan stick model. (**D**) 2D illustration **(*E*)-2f** shows two crucial interactions with the two essential amino acid residues: (**E**) the key for different interactions shown in 2D depiction.

Prior to docking our compounds, the Molecular Operating Environment (MOE) was able to reproduce the pose of the native ligand within the active site of GSK3β protein as demonstrated in Figure 6A, where both the native and re-docked ligands made almost the same types of interactions with the critical amino acid residues. The re-docked ligand’s binding free energy (ΔG) equals −7.4 Kcal/mol with an acceptable Root Mean Square Deviation (RMSD) value of 1.4Å (Table 1).

**Figure 7:**
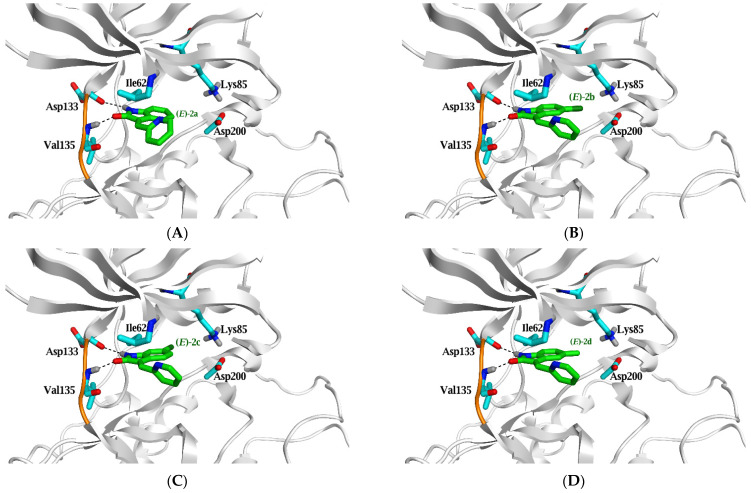

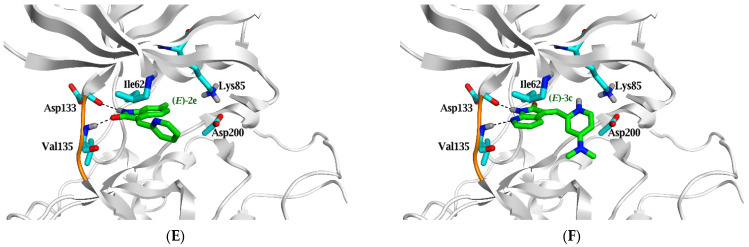
The top-ranked pose for compounds **2a** (**A**)**, 2b** (**B**)**, 2c** (**C**)**, 2d** (**D**)**, 2e** (**E**) and **3c** (**F**) (green stick model) in their ***E*** configuration within the active site of GSK3β (PDB ID: 4IQ6) predicted by MOE 2019.01. Hydrogen bonds are shown as black dashed lines, and the critical amino acid residues are displayed as a cyan stick model.

The top-ranked docking poses of indolin-2-one based compounds (**2a–e**) are depicted in Figure 7A–E. The NH group in the indolin-2-one moiety makes a weak H-bond with the critical residue (Asp 133) at the ATP binding site at the hinge region with the binding free energy −0.5 to −3.2 Kcal/mol. These binding energies are feeble compared to the native ligand, where its NH group makes a strong H-bond with the same residue (Asp133) with a binding free energy of −4.5 Kcal/mol.

**Figure 8:**
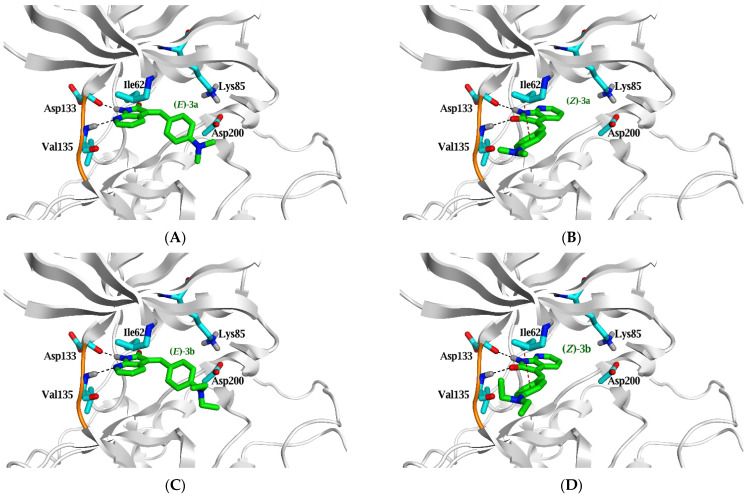

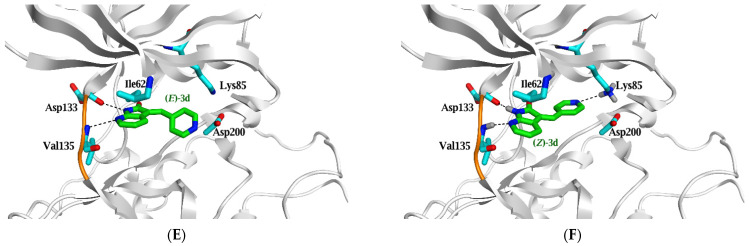
Comparison between the binding mode of ***E*** and ***Z*** configurations for compounds **3a** (**A**,**B**)**, 3b** (**C**,**D),** and **3d** (**E**,**F**) (green stick model) within the active site of GSK3β (PDB ID: 4IQ6) predicted by MOE 2019.01. Hydrogen bonds are shown as black dashed lines, while H–π interactions are red dashed lines with the critical amino acid residues (cyan stick model).

Remarkably, only the ***E*** configuration of azaindolin-2-one based compounds (**2f** and **3a–d**) can make these two strong interactions. In contrast, their ***Z*** configuration produces weak interactions due to incorporating amidic carbonyl group in the interaction rather than the strong H-bonds of the *N* atom of azaindolin-2-one moiety (Figure 8A–F).

### 2.4. Tau Aggregation Inhibition in a Cell Model of Tauopathy and Western Blot Analysis

**Figure 9:**
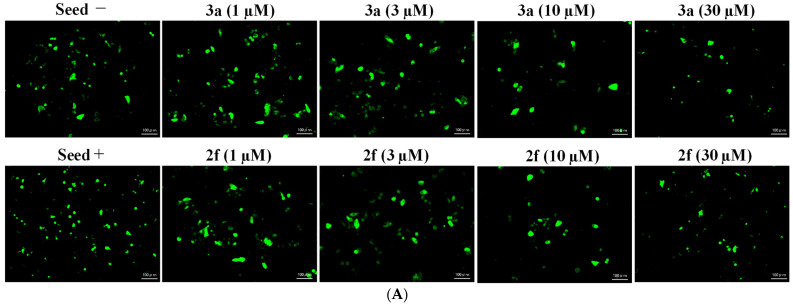

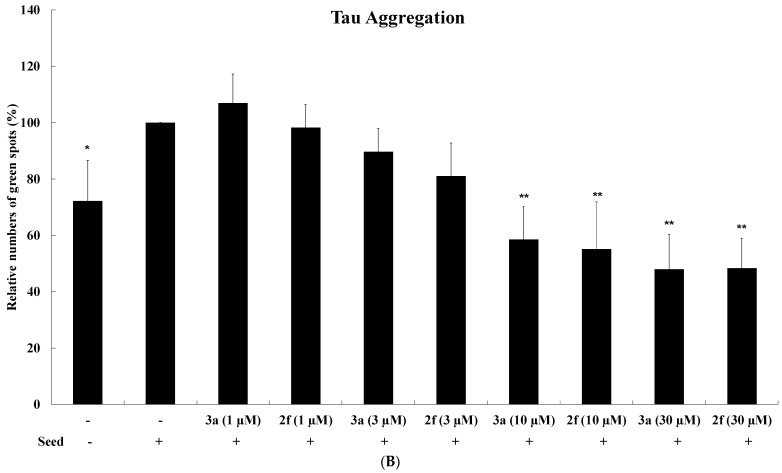
Anti-tau aggregation activity of compounds **(*E*)-2f** and **3a** at 1, 3, 10, and 30 µM concentrations. (**A**) Tau proteins were stained with green fluorescence in HeLa cells transfected with the expression vector of tau 2N4R isoform carrying P301L mutation and incubated with the compounds. Microscopic observation was performed, and photos were shown on a 100 µm scale. Seed negative (seed−) and seed positive (seed+) indicate the absence and presence of fibrils of tau protein with P301L mutation, respectively. (**B**) Relative green spots per area (%) after exposure of Hela cells to compounds **(*E*)-2f** and **3a** are compared to seed positive (seed+ = 100%). ImageJ counted more than 100 spots in the same area in each sample. *: denotes significant difference from control (seed+) at *p* < 0.05, **: denotes significant difference from control (seed+) at *p* < 0.01.

Aggregation of Tau is observed using Hela cells transduced with the expression vector of tau 2N4R isoform carrying P301L mutation and fibrils of tau protein with P301L mutation as seed [36,37]. We tested the anti-aggregation effects of **(*E*)-2f** and **3a** at four different concentrations, namely (1 µM, 3 µM, 10 µM, 30 µM), the measurements combined with negative seed (negative control = normal aggregation level), and positive seed (positive control = AD aggregation levels). In other words, seed— and + indicate the absence and presence of fibrils of tau protein with P301L mutation as seed, respectively. At 10 µM concentration, compound **(*E*)-2f** showed a significant anti-aggregation effect and even had less fibrillization than the negative control, while at 3 µM concentration it has moderate efficacy, similar to standard compound **3a**, and demonstrated no efficacy at 1 µM (Figure 9). Surprisingly, at 30 µM concentrations, both **(*E*)-2f** and **3a** completely protected Hela cells from tau aggregates (Figure S41).

**Figure 10:**
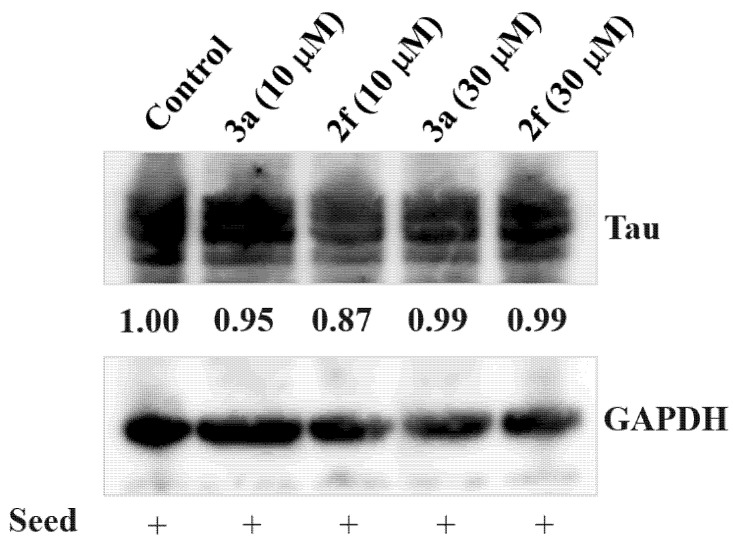
Effect of compounds **(*E*)-2f** and **3a** on Tau levels. HeLa cells transfected with the expression vector of tau 2N4R isoform carrying P301L mutation and incubated with compounds **(*E*)-2f** and **3a** at 10 and 30 µM concentrations for 24 h. Then Hela cells were lysed, and the lysates were analyzed by Western blotting. The relative amount of total tau expressed in the total cell lysate of Hela cells of each lane was quantified by ImageJ. The total tau level of Hela cells with positive seed (seed+) alone was calculated from total tau after GAPDH normalization and is presented as Control (1.00).

Western blot analysis was then used to confirm the amount of the total tau in Hela cells used for microscopic observation. The lysate of Hela cells, after incubation with compounds **(*E*)-2f** and **3a** at 10 and 30 µM concentrations, were analyzed by immunoblotting using the same anti-Tau antibody (Figure S42). ImageJ was used to quantify the total tau at each concentration and compared to the total tau level of Hela cells with positive seed (seed+) alone as a control. As depicted in Figure 10, the total tau level at each concentration of our compounds demonstrated little or no difference compared to untreated Hela cells.

### 2.5. Selective Cytotoxicity on Cancer Cells and Normal Blood Cells

**(*E*)-2f** displayed non-considerable cytotoxicity on peripheral blood mononuclear cells (PBMC) at an even concentration of more than 300 µM. Similarly, compound **3a** showed IC_50_ at 94.55 µM, as shown in Table 2. Additionally, **(*E*)-2f** moderately suppressed K562 at 4.7, U251 at 10.3, and HCT116 at 9.8 µM, while **(*E*)-2f** did not suppress A375 cell lines up to 100 µM (Figures S36–S40).

## 3. Discussion

The structural cores of our synthesized compounds are as follows: (a) the first one is an oxindole ring or its bioisostere 1,3-dihydro-2H-pyrrolo [2,3-b] pyridine-2-one ring, (b) a methylene linker, and (c) an aryl substitution which is attached to the methylene linker. Following the literature-known condensation reaction “Knoevenagel condensation”, we applied a strategy to optimize the main parts of our targeted compounds. Interestingly, all target compounds produced by 24 h refluxing of different azaindole or substituted oxindoles with the corresponding aldehyde, except compound **3c**, were not produced with the reflux method. Instead, stirring in an ice bath for two hours followed by stirring the reaction mixture at room temperature for 12 h yielded our target compound **3c**.

When pyridine-2-carbaldehyde was used in condensation, only (***E***) isomer was produced; however, when pyridine-4-carbaldehyde or amine substituted benzaldehyde was used, a combination of ***E***/***Z*** isomers was produced. The unusual behavior of 2-pyridine derivatives compared to 4-pyridyl amine substituted benzaldehyde equivalents can be attributed to the secondary nonbonded contact between the nitrogen and the 4-H oxindole proton, which stabilizes the coplanar arrangement of the aromatic rings in 2-pyridine derivatives [27].

In the GSK3β inhibitory assay, compound **(*E*)-2f** had the best inhibitory activity on GSK3β with an IC_50_ of 1.7 μM. Preliminary SAR of our compounds revealed that chloro substitution at positions 5 and 6 on the oxindole ring enhanced activity compared to the non-substituted oxindole. However, non-substituted oxindole showed more efficacy than 5 or 6 fluorooxindole. Surprisingly, the efficacy was significantly increased when the oxindole was replaced with its N bioisostere nucleus. Furthermore, N bioisostere demonstrated the greatest potency.

Moreover, 2-pyridyl is more effective than 4 pyridyl substituent and amine substituted phenyl. The amine substitution on the phenyl ring is more potent to be dimethylamine than diethylamine. In brief, the superior activity of **(*E*)-2f** is mainly due to azaindolin-2-one chemotype, while 2-pyridyl substitution enhances this GSK3β inhibitory activity.

Molecular simulation studies of compounds **2a–f** and **3a–d** were performed using Molecular Operating Environment to explain the potency difference between indolin-2-one and azaindolin-2-one scaffolds on GSK3 inhibition activity in vitro (MOE). The docking interactions and orientations of compounds **2a–e** demonstrates their weak inhibitory activity against GSK3β in vitro. Briefly, indolin-2-one based compounds (**2a–e**) interact with the two essential amino acids (Asp 133 and Val 135) through relatively weak H-bonds compared to the native ligand. On the contrary, azaindolin-2-one, containing compounds (**2f** and **3a–d**), made two strong H-bonds with the two essential amino acids (Asp 133 and Val 135) with their amidic NH group and N atom of the azaindolin-2-one moiety.

Compounds **2f** and **3c** are the only azaindolin-2-one-based compounds in the E configuration. Other azaindolin-2-one compounds (**3a**, **3b**, and **3d**) are a mixture of ***E*** and ***Z*** configurations. It is evident that the ***E*** configuration is responsible for the actual inhibitory activity of azaindolin-2-one compounds. Furthermore, the docking poses of the ***Z*** configuration of azaindolin-2-one compounds are very similar to those of the docked indolin-2-one compounds (**2a–e**). Therefore, compounds **2f** and **3c** are expected to have a better binding affinity to GSK3β protein than other azaindolin-2-one compounds (**3a**, **3b**, and **3d**). Compound **2f** demonstrated the most significant inhibitory activity against GSK3 in vitro; this superior activity could be attributed to its ability to form additional hydrophobic interactions and its two strong H-bonds with the two critical amino acids.

Compound **3a** has previously been demonstrated to have potent binding affinities in vitro on alpha-synuclein (α-syn), beta-amyloid (Aβ), and tau fibrils [9]. Therefore, we decided to validate the anti-aggregating characteristics of **(*E*)-2f** compared to **3a** as a standard. Indeed, recombinant shortened tau isoforms have been more popular in recent years for in vitro tau aggregation. Adding anionic cofactors (e.g., heparin, arachidonic acid) results in the development of fibrils that mirror those found in the brains of AD patients. Consequently, we used Tau Aggregation Assay Kits to monitor the fibrillization of tau-transfected HeLa cells using fluorescence assay techniques. In this assay, the numbers of aggregates were assessed. Some aggregates were detected in the cells without fibrils as seeds, which may be formed by something naturally present in the cells. The number of aggregates increases when the expression vector of tau 2N4R isoform carrying P301L mutation (fibrils of tau protein with P301L mutation) is transduced into Hela cells, and our compounds suppress the aggregation. Both compounds, **(*E*)-2f** and **3a**, have a concentration-dependent inhibitory activity against tau aggregation. Consequently, azaindolin-2-one scaffold in both compounds seems to be essential for anti-tau aggregation, as well as the previous GSK3β inhibition assay.

One possible explanation of this anti-tau aggregation is that these compounds decrease the amount of tau expressed in Hela cells. Therefore, we performed a western blot analysis to confirm this hypothesis. The results revealed that both compounds, **(*E*)-2f** and **3a,** at two different concentrations have minimal effect on total tau levels compared to untreated Hela cells. Consequently, the anti-tau aggregation effect of our compounds is due to their chaperon effect (direct binding to Tau) rather than lowering the tau expression.

Based on the findings, it can be concluded that our compounds have anti-tau aggregation activity, regardless of aggregate shapes. This tau aggregates formation is considered one of the models of tau fibrils formation.

The active site of GSK3β is highly similar to the active sites of cyclin-dependent kinase (CDK) family members, which might lead promiscuous inhibitors to induce undesirable effects [27]. CDK inhibition has been linked to peripheral blood mononuclear cells (PBMC) toxicity. To assess the cytotoxicity of our compounds on normal cells, we tested **(*E*)-2f**, which has been identified as a potent GSK3β inhibitor against PBMC viability and some selected cancerous cell lines. Moreover, compound **3a** was used as a standard. **(*E*)-2f** is harmless to PBMC at doses much beyond their IC_50_ values in a functional GSK3β test. In addition, **(*E*)-2f** moderately suppressed selected cancer cell lines such as K562, U251, and HCT116, while it needs a concentration over 100 µM to suppress A375.

## 4. Materials and Methods

### 4.1. Chemistry

All chemical compounds were purchased from Merch (Tokyo, Japan), Alfa Aesar (Ward Hill, MA, USA), Tokyo Chemical Industry (Tokyo, Japan), Kanto Chemical (Tokyo, Japan), Nacalai Tesque (Kyoto, Japan) and FUJFILM-Wako (Osaka, Japan). Thin-layer chromatography (TLC) was performed to follow the progress of the reactions on pre-coated plates (Merck TLC sheets silica 60 F254). NMR spectra (BRUKER 600 MHz) used TMS as a reference. A JEOL JMSDX303HF recorded mass spectra (EI), and high-resolution mass spectra (HRMS). HRMS were recorded using positive fast atom bombardment (FAB) with 3-nitrobenzyl alcohol (NBA) as the matrix.

#### 4.1.1. General Procedure for the Synthesis of Compounds **2a–f**

A solution of **1a–e** (1 mmol) in absolute ethanol (30 mL) was added to picolinaldehyde (1 mmol) followed by a catalytic amount of piperidine (2 drops), and the mixture was refluxed for 8–12 h. After cooling to room temperature, a colored precipitate was collected by filtration, washed with absolute cold ethanol then diethyl ether. The resulting product was recrystallized from methanol to afford **2a–e**.

(*E*)-3-(pyridin-2-ylmethylene)indolin-2-one (**2a**) [27].

Brown powder; 155 mg (70% yield); ^1^H NMR (600 MHz, DMSO) δ 10.61 (s, 1H), 8.99 (d, *J* = 7.7 Hz, 1H), 8.88 (d, *J* = 3.7 Hz, 1H), 7.95 (td, *J* = 7.7, 1.8 Hz, 1H), 7.87 (d, *J* = 7.9 Hz, 1H), 7.56 (s, 1H), 7.47 (ddd, *J* = 7.6, 4.7, 1.1 Hz, 1H), 7.28 (td, *J* = 7.6, 1.2 Hz, 1H), 6.98 (td, *J* = 7.6, 1.1 Hz, 1H), 6.86 (d, *J* = 7.4 Hz, 1H); ^13^C NMR (150 MHz, DMSO) δ 169.23, 153.21, 149.63, 143.57, 137.21, 133.60, 130.79, 129.26, 128.35, 127.87, 124.09, 121.48, 121.19, 109.56; MS (EI+) *m/z* 221 (M-H)^+^. Refer to Figures S1–S3.

(*E*)-5-chloro-3-(pyridin-2-ylmethylene)indolin-2-one (**2b**).

Orange powder; 145 mg (56% yield); ^1^H NMR (600 MHz, DMSO) δ 10.75 (s, 1H), 9.13 (d, *J* = 2.3 Hz, 1H), 8.88 (ddd, *J* = 4.7, 1.6, 0.5 Hz, 1H), 7.97 (td, *J* = 7.7, 1.8 Hz, 1H), 7.90 (d, *J* = 7.8 Hz, 1H), 7.62 (s, 1H), 7.49 (ddd, *J* = 7.6, 4.7, 1.2 Hz, 1H), 7.32 (dd, *J* = 8.3, 2.3 Hz, 1H), 6.87 (d, *J* = 8.3 Hz, 1H); ^13^C NMR (150 MHz, DMSO) δ 168.95, 152.80, 149.59, 142.32, 137.46, 135.25, 130.19, 129.05, 128.31, 127.55, 125.09, 124.59, 123.05, 110.92; MS (EI+) *m/z* 255 (M-H)^+^. Refer to Figures S4–S6.

(*E*)-6-chloro-3-(pyridin-2-ylmethylene)indolin-2-one (**2c**).

Green powder; 159 mg (62% yield); ^1^H NMR (600 MHz, DMSO) δ 10.77 (s, 1H), 9.06 (d, *J* = 8.3 Hz, 1H), 8.87 (d, *J* = 3.7 Hz, 1H), 7.95 (td, *J* = 7.7, 1.8 Hz, 1H), 7.88 (d, *J* = 7.8 Hz, 1H), 7.59 (s, 1H), 7.47 (ddd, *J* = 7.5, 4.7, 1.0 Hz, 1H), 7.02 (dd, *J* = 8.3, 2.0 Hz, 1H), 6.87 (d, *J* = 2.0 Hz, 1H); ^13^C NMR (150 MHz, DMSO) δ 169.23, 152.95, 149.67, 144.92, 137.32, 134.79, 134.35, 129.35, 128.74, 128.05, 124.33, 120.91, 120.44, 109.56; MS (EI+) *m/z* 255 (M-H)^+^. Refer to Figures S7–S9.

(*E*)-5-fluoro-3-(pyridin-2-ylmethylene)indolin-2-one (**2d**).

Orange powder; 160 mg (67% yield); ^1^H NMR (600 MHz, DMSO) δ 10.62 (s, 1H), 8.92 (dd, *J* = 10.2, 2.7 Hz, 1H), 8.89 (d, *J* = 3.7 Hz, 1H), 7.95 (td, *J* = 7.7, 1.7 Hz, 1H), 7.88 (d, *J* = 7.8 Hz, 1H), 7.59 (s, 1H), 7.47 (ddd, *J* = 7.5, 4.8, 0.9 Hz, 1H), 7.11 (td, *J* = 8.9, 2.8 Hz, 1H), 6.83 (dd, *J* = 8.5, 4.7 Hz, 1H); ^13^C NMR (151 MHz, DMSO) δ 169.24, 158.08, 156.53, 152.88, 149.67, 139.92, 139.91, 137.43, 135.02, 128.99, 128.95, 128.93, 124.48, 122.60, 122.53, 117.06, 116.90, 115.05, 114.87, 110.17, 110.12; MS (EI+) *m/z* 239 (M-H)^+^; HRMS (EI+) Calcd for C_14_H_9_FN_2_O: 240.0699; found: 240.0675. Refer to Figures S10–S13.

(*E*)-6-fluoro-3-(pyridin-2-ylmethylene)indolin-2-one (**2e**).

Green powder; 135 mg (56% yield); ^1^H NMR (600 MHz, DMSO) δ 10.79 (s, 1H), 9.11 (dd, *J* = 8.6, 6.1 Hz, 1H), 8.89–8.85 (m, 1H), 7.95 (td, *J* = 7.7, 1.8 Hz, 1H), 7.87 (d, *J* = 7.8 Hz, 1H), 7.54 (s, 1H), 7.47 (ddd, *J* = 7.6, 4.7, 1.1 Hz, 1H), 6.84–6.76 (m, 1H), 6.69 (dd, *J* = 9.1, 2.5 Hz, 1H). ^13^C NMR (150 MHz, DMSO) δ 169.66, 164.44, 162.80, 153.09, 149.65, 145.60, 145.51, 137.34, 133.26, 133.23, 130.05, 129.99, 128.51, 128.14, 128.13, 124.27, 118.07, 118.05, 107.53, 107.38, 97.75, 97.57; MS (EI+) *m/z* 239 (M-H)^+^. Refer to Figures S14–S16.

(*E*)-3-(pyridin-2-ylmethylene)-1,3-dihydro-2H-pyrrolo [2,3-b]pyridin-2-one (**2f**).

Red powder; 176 mg (79% yield); ^1^H NMR (600 MHz, DMSO) δ 11.21 (s, 1H), 9.25 (dd, *J* = 7.6, 1.6 Hz, 1H), 8.88 (dd, *J* = 4.7, 1.0 Hz, 1H), 8.12 (dd, *J* = 5.2, 1.7 Hz, 1H), 7.95 (td, *J* = 7.6, 1.8 Hz, 1H), 7.89 (d, *J* = 7.8 Hz, 1H), 7.68 (s, 1H), 7.48 (ddd, *J* = 7.5, 4.7, 1.2 Hz, 1H), 7.01 (dd, *J* = 7.6, 5.2 Hz, 1H); ^13^C NMR (150 MHz, DMSO) δ 168.88, 157.74, 152.78, 149.78, 148.54, 137.39, 135.40, 135.13, 128.85, 127.62, 124.54, 117.60, 116.05; MS (EI+) *m/z* 222 (M-H)^+^; HRMS (EI+) Calcd for C_13_H_9_N_3_O: 223.0746; found: 223.0724. Refer to Figures S17–S20.

#### 4.1.2. General Procedure for the Synthesis of Compounds **3a–d**

A solution of 1e (1 mmol) in absolute ethanol (30 mL) was added to corresponding aldehyde (1 mmol) followed by a catalytic amount of piperidine (2 drops), and the mixture was refluxed for 8–12 h. After cooling to room temperature, a colored precipitate was collected by filtration, washed with absolute cold ethanol, then diethyl ether. The resulting product was recrystallized from methanol to afford **3a**,**b**, and **d**. Compound **3c** was obtained without reflux by stirring in an ice bath for two hours, then room temperature for 12 h.

(*E*/*Z*)-3-(4-(dimethylamino)benzylidene)-1,3-dihydro-2*H*-pyrrolo [2,3-b]pyridin-2-one (**3a**) [9].

Red powder; 186 mg (70% yield); ^1^H NMR (600 MHz, DMSO) δ 11.05 (s, 1H), 8.04 (m, 2H), 7.65 (d, *J* = 9.2 Hz, 2H), 7.64 (s, 1H), 6.93 (dd, *J* = 7.6, 5.2 Hz, 1H), 6.82 (d, *J* = 8.9 Hz, 2H), 3.04 (s, 6H); *E*:*Z* ratio is 80:20; ^13^C NMR (150 MHz, DMSO) δ 168.98, 156.04, 151.70, 146.46, 139.20, 132.37, 128.22, 120.64, 119.86, 117.00, 116.19, 111.55, 39.59; MS (EI+) *m/z* 265 (M)^+^. Refer to Figures S21–S23.

(*E*/*Z*)-3-(4-(diethylamino)benzylidene)-1,3-dihydro-2*H*-pyrrolo [2,3-b]pyridin-2-one (**3b**).

Dark red powder; 131 mg (44% yield); ^1^H NMR (600 MHz, DMSO) δ 11.02 (s, 1H), 8.08–8.01 (m, 2H), 7.63 (d, *J* = 8.9 Hz, 2H), 7.61 (s, 1H), 6.92 (dd, *J* = 7.6, 5.2 Hz, 1H), 6.78 (d, *J* = 9.0 Hz, 2H), 3.43 (q, *J* = 6.9 Hz, 4H), 1.13 (t, *J* = 7.0 Hz, 6H); *E*:*Z* ratio is 90:10; ^13^C NMR (150 MHz, DMSO) δ 169.07, 155.90, 149.33, 146.23, 139.22, 132.87, 128.20, 119.92, 119.11, 116.97, 116.27, 111.01, 43.84, 12.44; MS (EI+) *m/z* 293 (M)^+^; HRMS (EI+) Calcd for C_18_H_19_N_3_O: 293.1528; found: 293.1517. Refer to Figures S24–S27.

(*E*)-3-((5-(dimethylamino)pyridin-2-yl)methylene)-1,3-dihydro-2*H*-pyrrolo [2,3-b]pyridin-2-one (**3c**).

Reddish brown powder; 110 mg (41% yield); ^1^H NMR (600 MHz, DMSO) δ 11.17 (s, 1H), 9.41 (dd, *J* = 7.5, 1.2 Hz, 1H), 8.41 (d, *J* = 5.8 Hz, 1H), 8.11 (dd, *J* = 5.1, 1.3 Hz, 1H), 7.62 (s, 1H), 7.22 (d, *J* = 2.3 Hz, 1H), 7.02 (dd, *J* = 7.5, 5.2 Hz, 1H), 6.67 (dd, *J* = 5.8, 2.5 Hz, 1H), 3.02 (s, 6H); ^13^C NMR (150 MHz, DMSO) δ 169.14, 157.48, 154.74, 152.80, 149.54, 148.05, 137.73, 135.63, 126.75, 117.49, 116.38, 112.65, 106.78, 38.72; MS (FAB+) *m/z* 267 (M+H)^+^; HRMS (FAB+) Calcd for C_15_H_15_N_4_O: 267.1240; found: 267.1268. Refer to Figures S28–S31.

(*E*/*Z*)-3-(pyridin-4-ylmethylene)-1,3-dihydro-2H-pyrrolo [2,3-b]pyridin-2-one (**3d**).

Brown powder; 121mg (54% yield); ^1^H NMR (600 MHz, DMSO) δ 11.32 (s, 1H), 8.72 (dd, *J* = 4.4, 1.6 Hz, 2H), 8.11 (dd, *J* = 5.2, 1.5 Hz, 1H), 7.68 (s, 1H), 7.67 (dd, *J* = 7.7, 1.5 Hz, 1H), 7.63 (ddd, *J* = 4.4, 1.5, 0.6 Hz, 2H), 6.89 (dd, *J* = 7.6, 5.2 Hz, 1H); *E*:*Z* ratio is 20:80; ^13^C NMR (150 MHz, DMSO) δ 167.68, 157.40, 150.27, 148.88, 141.71, 134.25, 130.14, 128.85, 123.14, 117.55, 114.73; MS (EI+) *m/z* 223 (M)^+^; HRMS (EI+) Calcd for C_13_H_9_N_3_O: 223.0746; found: 223.0717. Refer to Figures S32–S35.

### 4.2. GSK-3β Inhibition Assay

According to the manufacturer’s protocol, the assay was conducted using GSK3β Kinase Enzyme System and ADP-Glo Kinase Assay (Promega Corporation, Madison, WI, USA) in 384-well plates. The kinase reaction was performed at room temperature using 2 ng/μL of human recombinant GSK3β, 0.2 μg/μL of GSK3 substrate (YRRAAVPPSPSLSRHSSPHQ (pS) EDEEE; amino acids 636–661), and 25 μM ATP in 4X kinase buffer containing 50 μM DTT. The compounds were added to enzyme solution and incubated for 2 h at room temperature. Then, kinase activity was quantified using the ADP-Glo Kinase Assay as previously described [38].

### 4.3. Molecular Docking Study

All the docking algorithms, ranking, and visualization processes were performed using Molecular Operating Environment (MOE) 2019.01 (Chemical Computing Group, Montreal, QC, Canada) software within the active site of GSK-3β (PDB ID: 4IQ6), downloaded from the Protein Data Bank [29]. The protein structure (PDB ID: 4IQ6) was prepared through the QuickPrep suite executed in MOE. Then, all chemical structures of the docked compounds were built, and their energies were minimized using the default force field, AMBER10. Before docking, the native co-crystallized ligand was re-docked into the binding site using the default parameters to validate the docking study at the binding site. The top-ranked re-docked pose has an RMSD value of 1.4 Å, and its energy score was −7.4 kcal/mol. Non-essential ligands and water molecules were removed before docking. The compounds were docked in the active site using rigid receptor docking protocol, where ligand conformations were placed in the active site with the Triangle Matcher method using AMBER10 as a default force field, finally ranking the generated poses by the London dG scoring function. The generated docking poses were reviewed, and the poses of the best binding affinities were considered. All poses were generated and labeled using MOE software.

### 4.4. Tau Aggregation Inhibition in a Cell Model of Tauopathy and Western Blot Analysis

According to the manufacturer’s protocol, the assay was performed using Tau Aggregation Assay Kit (Cosmo Bio, Tokyo, Japan [catalog number: TAU01]). Briefly, HeLa cells were transfected with pCMV-Tau(2N4R)-P301L (20 ng) using Lipofectamine 3000 (ThermoFisher Scientific, Waltham, MA, USA) and treated with F-Tau (RD)-P301L (fibrils of tau protein with P301L mutation as seed, included in the assay kit). Drugs were added after 6 h, and the cells were incubated for 1 day. Next, immunostaining was performed basically as previously described [39]. Tau proteins were stained with green fluorescence. In this staining, a TAU antibody (Proteintech, Rosemont, IL, USA [catalog number: 10274-1-AP]) was used as the first antibody, and goat anti-rabbit IgG (H + L) highly cross-adsorbed secondary antibody, Alexa Fluor 488 ThermoFisher Scientific) was used as the second antibody. An All-in-One Fluorescence Microscope BIOREVO BZ-9000 (Keyence, Osaka, Japan) was used as the microscope. ImageJ counted more than 100 spots in the same area in each sample [40,41].

Western blot analysis was performed by lysing Hela cells in PBS/Laemmli sample buffer (1:1) as described previously [42]. TAU antibody (Proteintech, Rosemont, IL, USA [catalog number: 10274-1-AP]) or GAPDH antibody (0411) (Santa Cruz, Dallas, TX, USA [catalog number: sc-47724]) was used. Chemiluminescence detected immunoreactivity using ImmunoStar LD (FUJIFILM-Wako, Osaka, Japan).

### 4.5. Selective Cytotoxicity on Cancer Cells and Normal Blood Cells

#### 4.5.1. Cells

The human cervical carcinoma cell line HeLa, obtained from the American Type Culture Collection (ATCC), was cultured in Dulbecco’s modified Eagle’s medium (DMEM) supplemented with 5% heat-inactivated fetal bovine serum (FBS). The human glioblastoma cell line U251 (obtained from ATCC), colon carcinoma cell line HCT116 [provided by the RIKEN BRC through the National Bio-Resource Project of the MEXT/AMED, Japan (RCB2979)], and human melanoma cell line A375, obtained from ATCC, were cultured in DMEM supplemented with 10% heat-inactivated FBS. The human chronic myelogenous leukemia cell line K562 and human peripheral blood mononuclear cells (PBMC), both cells obtained from ATCC, were cultured in RPMI 1640 supplemented with 10% heat-inactivated FBS described previously [43].

#### 4.5.2. MTT Assay

Cells were seeded in a 24-well plate, and the compounds were added. After incubation for 3 days, a solution (1.1 mg/mL) of 3-(4,5-dimethylthiazol-2-yl)-2,5-diphenyl-2H-tetrazolium bromide (MTT, from DOJINDO Laboratories, Kumamoto, Japan) was added, and MTT assay was performed as previously described [44].

## 5. Conclusions

For years, we have been interested in AD disease and its multifactorial nature. In the current study, we succeeded in synthesizing a multitargeted chemotype that can deal with the multifactorial nature of AD. We designed a unique, second-in-class chemical series of dual inhibitors that inhibit GSK3β and tau aggregation based on structural resemblances and known SAR data for GSK3β and Tau aggregation inhibitors. We chose to begin with oxindole and its *N* bioisostere, which have been previously reported as binding motifs for inhibiting GSK3β and preventing tau aggregates’ development, and destabilizing preexisting tau aggregates in a dose-dependent manner. Hybridizing these scaffolds with pyridyl group via methylene linker enhanced the GSK3β activity and ensured the anti-aggregation effect. This design has resulted in **(*E*)-2f**, a low molecular weight fragment with specificity and potency at two structurally different tau targets, GSK3β and a fibrillar protein, and is safe on normal cells. Our research identified azaindolin-2-one as a promising scaffold that could be used to develop future multitargeted therapies for AD.

## Figures and Tables

**Table 1:** Binding free energy (ΔG) to GSK3β in kcal/mol, IC_50_ in µM, ligand atoms, and amino acid residues forming interactions and the type, distance, and energy of these interactions for compounds **2a–f** and **3a–d** compared to the native ligand.

Ligand	ΔG in Kcal/mol (RMSD)	IC_50_ (µM)	Ligand	Interaction Parameters			
		GSK3β	Atoms	Interaction	AA Residue	δ(Å)	E (Kcal/mol)
Native ligand	---	0.058 *	N21	H-donor	Asp 133	2.78	−4.5
			N22	H-donor	Asp 200	3.02	−2.0
			CL23	H-donor	Glu 97	3.18	−0.6
			N19	H-acceptor	Val 135	3.07	−2.2
			CL23	H-acceptor	Lys 85	3.6	−0.8
Re-docked	−7.4 (1.4)		N21	H-donor	Asp 133	2.9	−4.4
			N22	H-donor	Asp 200	3.3	−1.3
			CL23	H-donor	Glu 97	3.36	−0.5
			N19	H-acceptor	Val 135	3.19	−2.1
			CL23	H-acceptor	Lys 85	3.72	−0.7
**(*E*)-2a**	−5.2 (1.6)	>10	N7	H-donor	Asp 133	3.58	−0.5
			O10	H-acceptor	Val 135	2.98	−3.3
**(*E*)-2b**	−6.0 (1.5)	>10	N7	H-donor	Asp 133	3.16	−3.2
			O10	H-acceptor	Val 135	2.98	−3.5
**(*E*)-2c**	−5.9 (0.8)	>10	N7	H-donor	Asp 133	3.34	−1.9
			O10	H-acceptor	Val 135	3.03	−3.3
**(*E*)-2d**	−5.7 (1.9)	>10	N7	H-donor	Asp 133	3.2	−3.1
			O10	H-acceptor	Val 135	3	−3.4
**(*E*)-2e**	−5.6 (1.1)	>10	N7	H-donor	Asp 133	3.27	−2.5
			O10	H-acceptor	Val 135	3.01	−3.3
**(*E*)-2f**	−6.0 (1.7)	1.7	N7	H-donor	Asp 133	2.97	−4.8
			N3	H-acceptor	Val 135	3.29	−2.7
**(*E*)-3a**	−6.3 (0.8)	1.9	N7	H-donor	Asp 133	2.93	−4.8
			N3	H-acceptor	Val 135	3.16	−3.2
**(*Z*)-3a**	−5.8 (1.6)		N1	H-donor	Asp 133	3.45	−1.6
			O10	H-acceptor	Val 135	3.43	−1.2
			6-ring	π-H	Ile 62	3.84	−0.5
**(*E*)-3b**	−6.5 (1.4)	3	N7	H-donor	Asp 133	2.97	−4.7
			N3	H-acceptor	Val 135	3.2	−3.0
**(*Z*)-3b**	−6.1 (1.8)		N1	H-donor	Asp 133	3.45	−1.6
			O10	H-acceptor	Val 135	3.4	−1.4
			6-ring	π-H	Ile 62	3.88	−0.6
**(*E*)-3c**	−6.4 (1.8)	3.2	N7	H-donor	Asp 133	3	−4.8
			N3	H-acceptor	Val 135	3.27	−2.7
**(*E*)-3d**	−5.9 (1.7)	5.9	N7	H-donor	Asp 133	2.96	−4.8
			N3	H-acceptor	Val 135	3.26	−2.8
**(*Z*)-3d**	−5.8 (1.2)		N1	H-donor	Asp 133	3.25	−1.6
			N8	H-acceptor	Val 135	3.4	−0.9
			N15	H-acceptor	Lys 85	3.54	−1.2
			6-ring	π-H	Asp 200	3.53	−0.5

* IC_50_ value of the co-crystalized ligand as reported.

**Table 2:** The cytotoxic effects of **(*E*)-2f** and **3a** on K562, U251, HCT-116, A375 cells, and PBMC after 72 h drug treatment.

IC50 (µM) after 72 h Drug Treatment					
Compound	K562	U251	HCT-116	A375	PBMC
**(*E*)-** **2f**	13.80	28.2	12.8	14.00	>300
**3a**	4.7	10.3	9.8	>100	94.55

## Data Availability

Data is contained within the article and supplementary material.

## Supplementary Materials

The following supporting information can be downloaded at: https://www.mdpi.com/article/10.3390/ph15040426/s1, Figure S1. 1H-NMR spectrum of compound **(*E*)-2a** (600 MHz, DMSO-d6); Figure S2. 13C-NMR spectrum of compound **(*E*)-2a** (150 MHz, DMSO-d6); Figure S3. MS (EI+) of compound **(*E*)-2a**; Figure S4. 1H-NMR spectrum of compound **(*E*)-2b** (600 MHz, DMSO-d6); Figure S5. 13C-NMR spectrum of compound **(*E*)-2b** (150 MHz, DMSO-d6); Figure S6. MS (EI+) of compound **(*E*)-2b**; Figure S7. 1H-NMR spectrum of compound **(*E*)-2c** (600 MHz, DMSO-d6); Figure S8. 13C-NMR spectrum of compound **(*E*)-2c** (150 MHz, DMSO-d6); Figure S9. MS (EI+) of compound **(*E*)-2c**; Figure S10. 1H-NMR spectrum of compound **(*E*)-2d** (600 MHz, DMSO-d6); Figure S11. 13C-NMR spectrum of compound **(*E*)-2d** (150 MHz, DMSO-d6); Figure S12. MS (EI+) of compound **(*E*)-2d**; Figure S13. HRMS (EI+) of compound **(*E*)-2d**; Figure S14. 1H-NMR spectrum of compound **(*E*)-2e** (600 MHz, DMSO-d6); Figure S15. 13C-NMR spectrum of compound **(*E*)-2e** (150 MHz, DMSO-d6); Figure S16. MS (EI+) of compound **(*E*)-2e**; Figure S17. 1H-NMR spectrum of compound **(*E*)-2f** (600 MHz, DMSO-d6); Figure S18. 13C-NMR spectrum of compound **(*E*)-2f** (150 MHz, DMSO-d6); Figure S19. MS (EI+) of compound **(*E*)-2f**; Figure S20. HRMS (EI+) of compound **(*E*)-2f**; Figure S21. 1H-NMR spectrum of compound **(*E*/*Z*)-3a** (600 MHz, DMSO-d6); Figure S22. 13C-NMR spectrum of compound **(*E*/*Z*)-3a** (150 MHz, DMSO-d6); Figure S23. MS (EI+) of compound **(*E*/*Z*)-3a**; Figure S24. 1H-NMR spectrum of compound **(*E*/*Z*)-3b** (600 MHz, DMSO-d6); Figure S25. 13C-NMR spectrum of compound **(*E*/*Z*)-3b** (150 MHz, DMSO-d6); Figure S26. MS (EI+) of compound **(*E*/*Z*)-3b**; Figure S27. HRMS (EI+) of compound **(*E*/*Z*)-3b**; Figure S28. 1H-NMR spectrum of compound **(*E*)-3c** (600 MHz, DMSO-d6); Figure S29. 13C-NMR spectrum of compound **(*E*)-3c** (150 MHz, DMSO-d6); Figure S30. MS (FAB+) of compound **(*E*)-3c**; Figure S31. HRMS (FAB+) of compound **(*E*)-3c**; Figure S32. 1H-NMR spectrum of compound **(*E*/*Z*)-3d** (600 MHz, DMSO-d6); Figure S33. 13C-NMR spectrum of compound **(*E*/*Z*)-3d** (150 MHz, DMSO-d6); Figure S34. MS (EI+) of compound **(*E*/*Z*)-3d**; Figure S35. HRMS (EI+) of compound **(*E*/*Z*)-3d**; Figure S36. Cell viability Dose-response curve for inhibition of K562 cells; Figure S37. Cell viability Dose-response curve for inhibition of U251 cells; Figure S38. Cell viability Dose-response curve for inhibition of HTC116 cells; Figure S39. Cell viability Dose-response curve for inhibition of A375 cells; Figure S40. Cell viability Dose-response curve for inhibition of PBMC cells; Figure S41. Effects of different concentrations of seed positive (seed +), **2f** (30 μM) and **3a** (30 μM) on Hela cell morphology photographed under an inverted microscope 24 h after treatment compared to untreated Hela cells, seed negative (seed -); Figure S42. The whole gel of western blot analysis showing the effect of different concentrations of **3a** (10 μM), **2f** (10 μM) **3a** (30 μM) and **2f** (30 μM) on total tau proteins (A), using GAPDH as a reference (B) after 24 h treatment compared to Hela cells, seed positive (seed +).
