# Supplementary material for: Discovery of Azaindolin-2-One as a Dual Inhibitor of GSK3β and Tau Aggregation with Potential Neuroprotective Activity
Source: Pharmaceuticals (Basel). 2022 Mar 31;15(4):426. doi: 10.3390/ph15040426 (PMC9029746; doi:10.3390/ph15040426)
Supplement: Supplementary file 1 [file pharmaceuticals-15-00426-s001.zip › pharmaceuticals-1580201-supplementary.pdf]

Supplementary Data:

Discovery of azaindolin-2-one as a dual inhibitor of GSK3β and tau aggregation with potential neuroprotective activity

Taha F.S. Ali[1,2*], Halil I. Ciftci[2,3], Mohamed O. Radwan[2,4], Eslam Roshdy[1,5], Ahmed M. Shawky[6,7], Mohammed A.S. Abourehab[8], Hiroshi Tateishi[2], Masami Otsuka[2,3] and Mikako Fujita[2*]

[1]Medicinal Chemistry Department, Faculty of Pharmacy, Minia University, Minia 61519, Egypt

[2]Medicinal and Biological Chemistry Science Farm Joint Research Laboratory, Faculty of Life Sciences, Kumamoto University, Kumamoto 862-0973, Japan; H.I.C. (hiciftci@kumamoto-u.ac.jp), M.O.R. (mohamedradwan@kumamoto-u.ac.jp), H.T. (htateishi@kumamoto-u.ac.jp), M.O. (motsuka@gpo.kumamoto-u.ac.jp)

[3]Department of Drug Discovery, Science Farm Ltd., Kumamoto 862-0976, Japan

[4]Chemistry of Natural Compounds Department, Pharmaceutical and Drug Industries Research Division, National Research Centre, Dokki 12622, Cairo, Egypt;

[5]Department of Chemistry, Graduate School of Science, Hiroshima University, Higashi-Hiroshima 739-8526, Hiroshima, Japan; E.R. (m211749@hiroshima-u.ac.jp)

[6]Science and Technology Unit (STU), Umm Al-Qura University, Makkah 21955, Saudi Arabia; A.M.S. (amesmail@uqu.edu.sa)

[7]Central Laboratory for Micro-analysis, Minia University, Minia 61519, Egypt

[8]Department of Pharmaceutics, Faculty of Pharmacy, Umm Al-Qura University, Makkah, 21955 Saudi Arabia; M.A.S.A. (maabourehab@uqu.edu.sa)

***Correspondence**: taha.ali@mu.edu.eg (T.F.S.A.); mfujita@kumamoto-u.ac.jp (M.F.); Tel.: +201069835295 (T.F.S.A.); +81-96-371-4622 (M.F.)

Table of contents

Figure	Title	page
S1	^{1}H-NMR spectrum of compound **(*E*)-2a** (600MHz, DMSO-*d*6)	4
S2	^{13}C-NMR spectrum of compound **(*E*)-2a** (150MHz, DMSO-*d*6)	5
S3	MS (EI+) of compound **(*E*)-2a**	6
S4	^{1}H-NMR spectrum of compound **(*E*)-2b** (600MHz, DMSO-*d*6)	7
S5	^{13}C-NMR spectrum of compound **(*E*)-2b** (150MHz, DMSO-*d*6)	8
S6	MS (EI+) of compound **(*E*)-2b**	9
S7	^{1}H-NMR spectrum of compound **(*E*)-2c** (600MHz, DMSO-*d*6)	10
S8	^{13}C-NMR spectrum of compound **(*E*)-2c** (150MHz, DMSO-*d*6)	11
S9	MS (EI+) of compound **(*E*)-2c**	12
S10	^{1}H-NMR spectrum of compound **(*E*)-2d** (600MHz, DMSO-*d*6)	13
S11	^{13}C-NMR spectrum of compound **(*E*)-2d** (150MHz, DMSO-*d*6)	14
S12	MS (EI+) of compound **(*E*)-2d**	15
S13	HRMS (EI+) of compound **(*E*)-2d**	16
S14	^{1}H-NMR spectrum of compound **(*E*)-2e** (600MHz, DMSO-*d*6)	17
S15	^{13}C-NMR spectrum of compound **(*E*)-2e** (150MHz, DMSO-*d*6)	18
S16	MS (EI+) of compound **(*E*)-2e**	19
S17	^{1}H-NMR spectrum of compound **(*E*)-2f** (600MHz, DMSO-*d*6)	20
S18	^{13}C-NMR spectrum of compound **(*E*)-2f** (150MHz, DMSO-*d*6)	21
S19	MS (EI+) of compound **(*E*)-2f**	22
S20	HRMS (EI+) of compound **(*E*)-2f**	23
S21	^{1}H-NMR spectrum of compound **(*E*/*Z*)-3a** (600MHz, DMSO-*d*6)	24
S22	^{13}C-NMR spectrum of compound **(*E*/*Z*)-3a** (150MHz, DMSO-*d*6)	25
S23	MS (EI+) of compound **(*E*/*Z*)-3a**	26
S24	^{1}H-NMR spectrum of compound **(*E*/*Z*)-3b** (600MHz, DMSO-*d*6)	27
S25	^{13}C-NMR spectrum of compound **(*E*/*Z*)-3b** (150MHz, DMSO-*d*6)	28
S26	MS (EI+) of compound **(*E*/*Z*)-3b**	29
S27	HRMS (EI+) of compound **(*E*/*Z*)-3b**	30
S28	^{1}H-NMR spectrum of compound **(*E*)-3c** (600MHz, DMSO-*d*6)	31
S29	^{13}C-NMR spectrum of compound **(*E*)-3c** (150MHz, DMSO-*d*6)	32
S30	MS (FAB+) of compound **(*E*)-3c**	33

S31	HRMS (FAB+) of compound **(*E*)-3c**	34
S32	^{1}H-NMR spectrum of compound **(*E*/*Z*)-3d** (600MHz, DMSO-*d6*)	35
S33	^{13}C-NMR spectrum of compound **(*E*/*Z*)-3d** (150MHz, DMSO-*d6*)	36
S34	MS (EI+) of compound **(*E*/*Z*)-3d**	37
S35	HRMS (EI+) of compound **(*E*/*Z*)-3d**	38
S36	Cell viability Dose-response curve for inhibition of K562 cells.	39
S37	Cell viability Dose-response curve for inhibition of U251 cells.	40
S38	Cell viability Dose-response curve for inhibition of HTC116 cells.	41
S39	Cell viability Dose-response curve for inhibition of A375 cells.	42
S40	Cell viability Dose-response curve for inhibition of PBMC cells.	43
S41	Effects of different concentrations of seed positive (seed +), **2f (30µM)** and **3a (30µM)** on Hela cell morphology photographed under an inverted microscope 24 hours after treatment; compared to untreated Hela cells, seed negative (seed -).	44
S42	The whole gel of western blot analysis showing the effect of compounds **3a (10µM)**, **2f (10µM) 3a (30µM)** and **2f (30µM)** on total tau proteins **(A),** using GAPDH as a reference **(B)** after 24 hours treatment compared to Hela cells, seed positive (seed +). The same membrane stained with anti-Tau in **(A)** and anti-GAPDH in **(B)**.	45

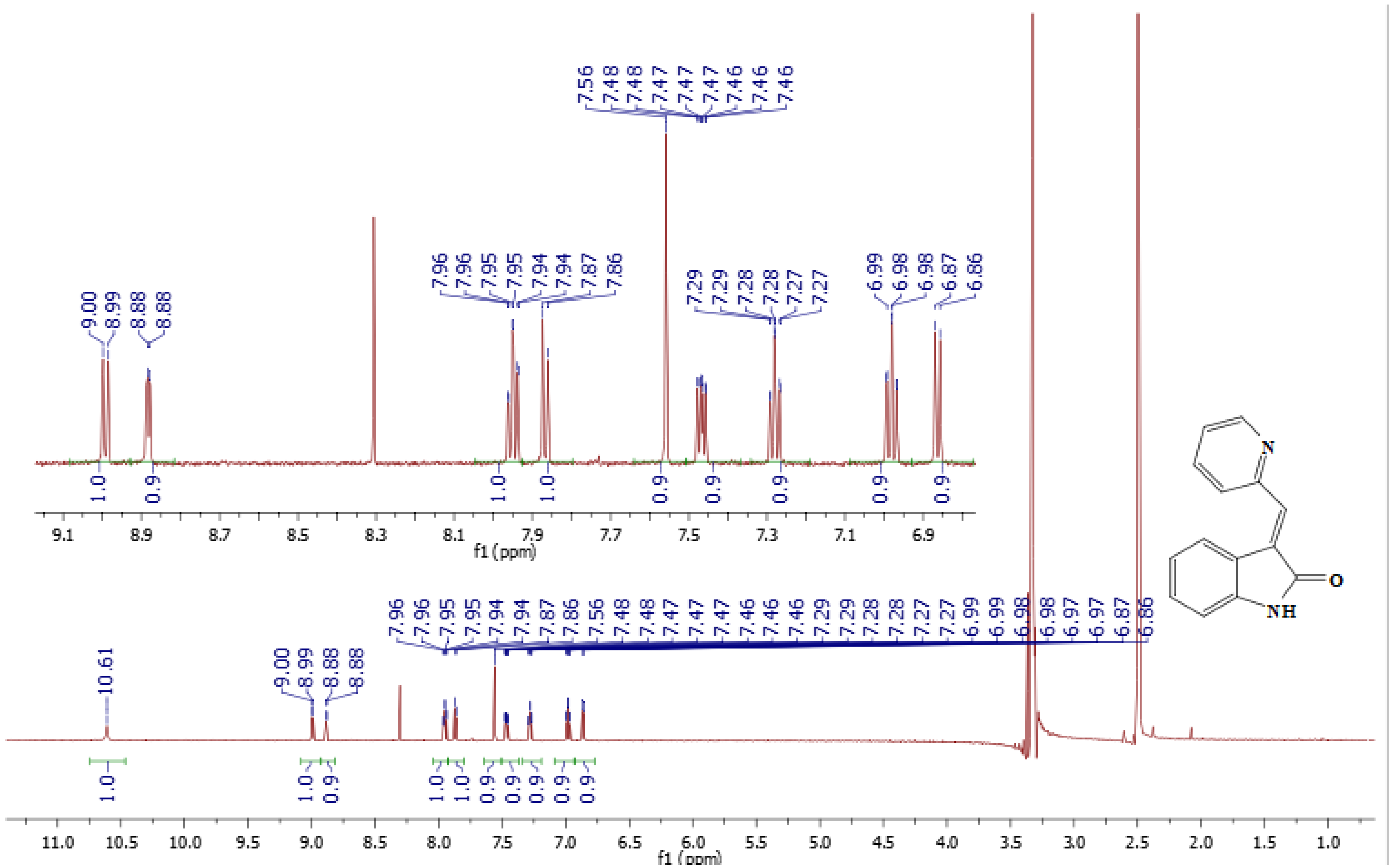

Figure S1. ^{1}H-NMR spectrum of compound **(*E*)-2a** (600MHz, DMSO-*d*6)

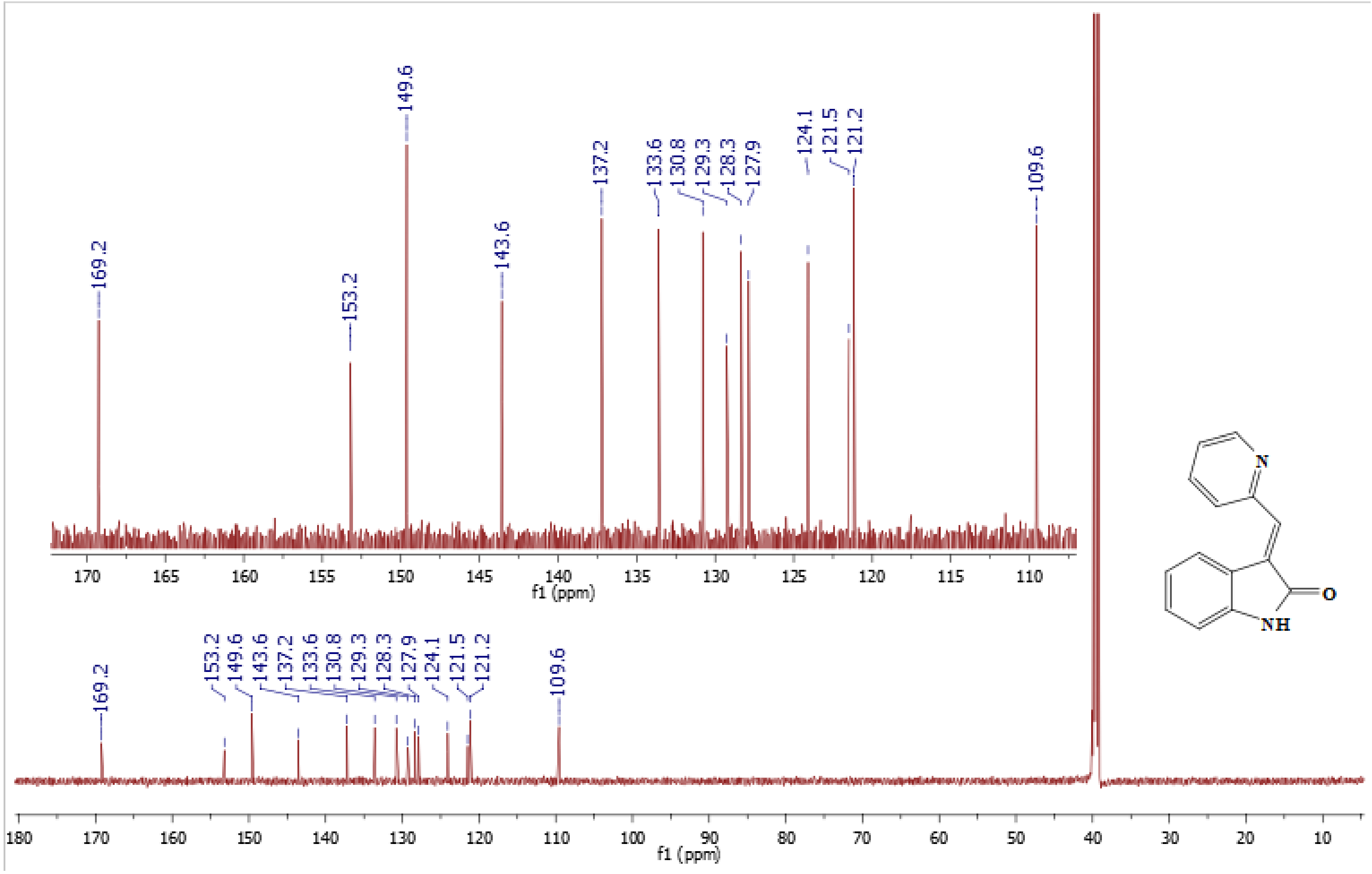

Figure S2. ^{13}C-NMR spectrum of compound **(*E*)-2a** (150MHz, DMSO-*d*6)

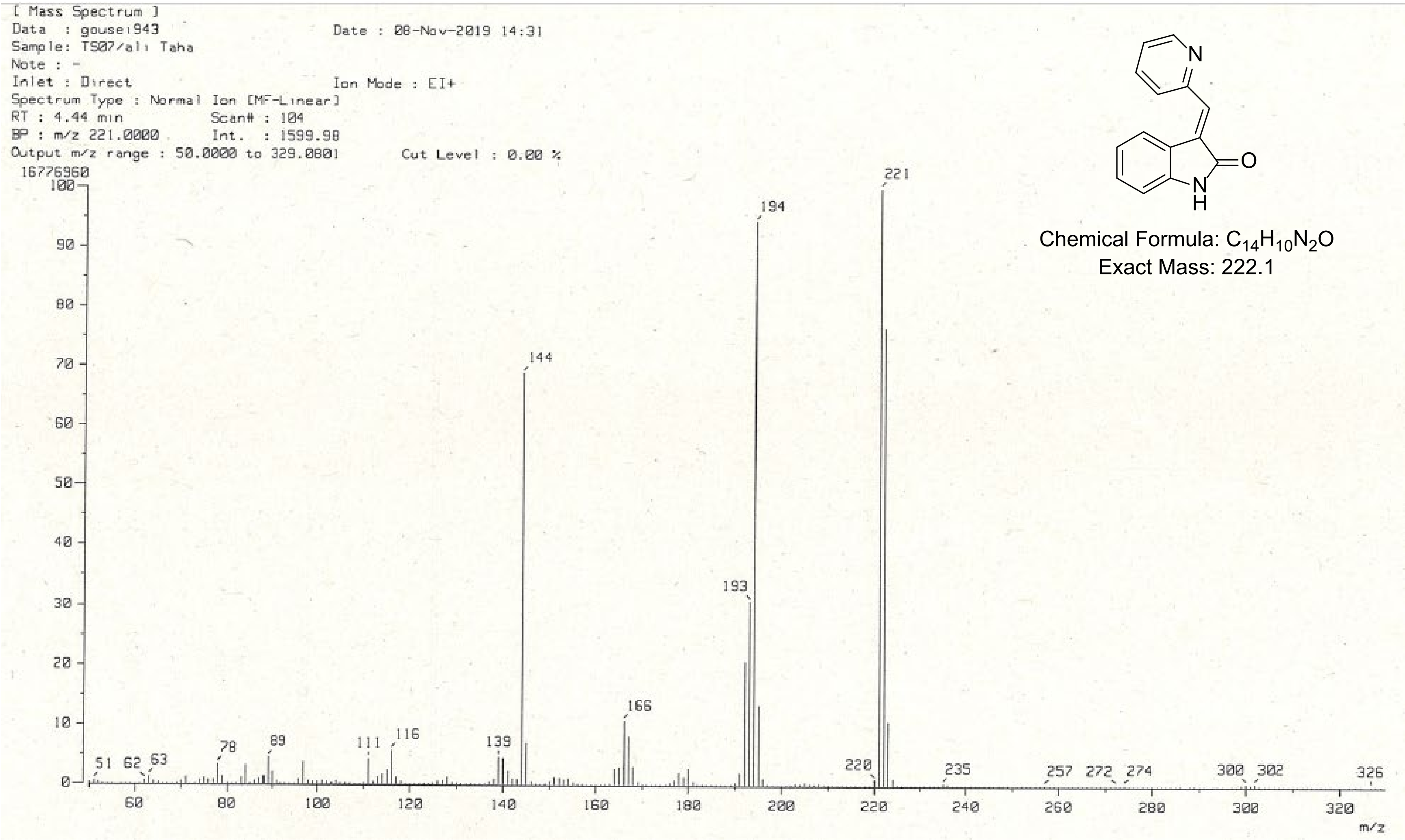

Chemical Formula: $C_{14}H_{10}N_2O$
Exact Mass: 222.1

Figure S3. MS (EI+) of compound ***(E)*-2a**

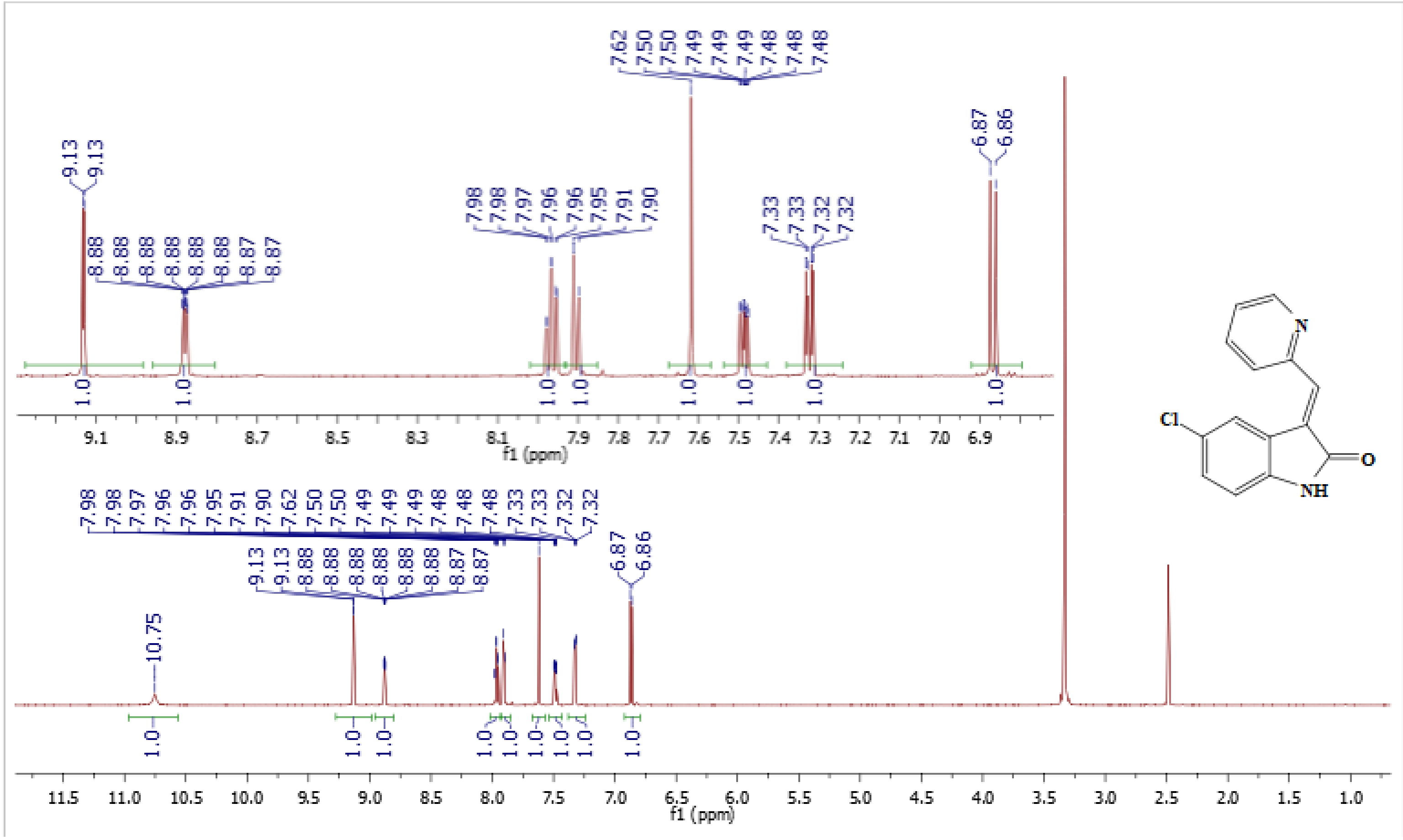

Figure S4. ^{1}H-NMR spectrum of compound ***(E)*-2b** (600MHz, DMSO-*d*6)

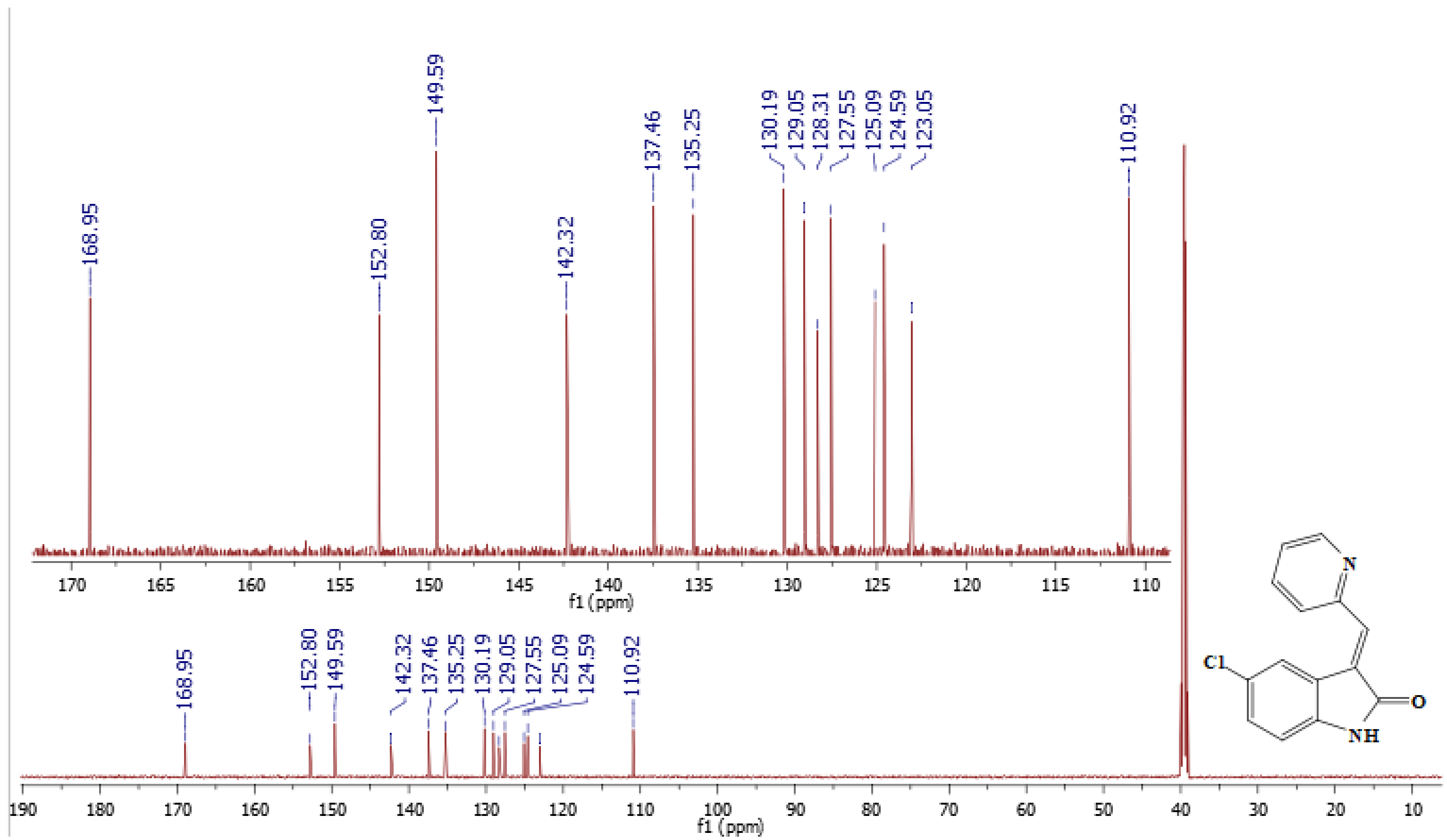

Figure S5. ^{13}C-NMR spectrum of compound **(*E*)-2b** (150MHz, DMSO-*d*6)

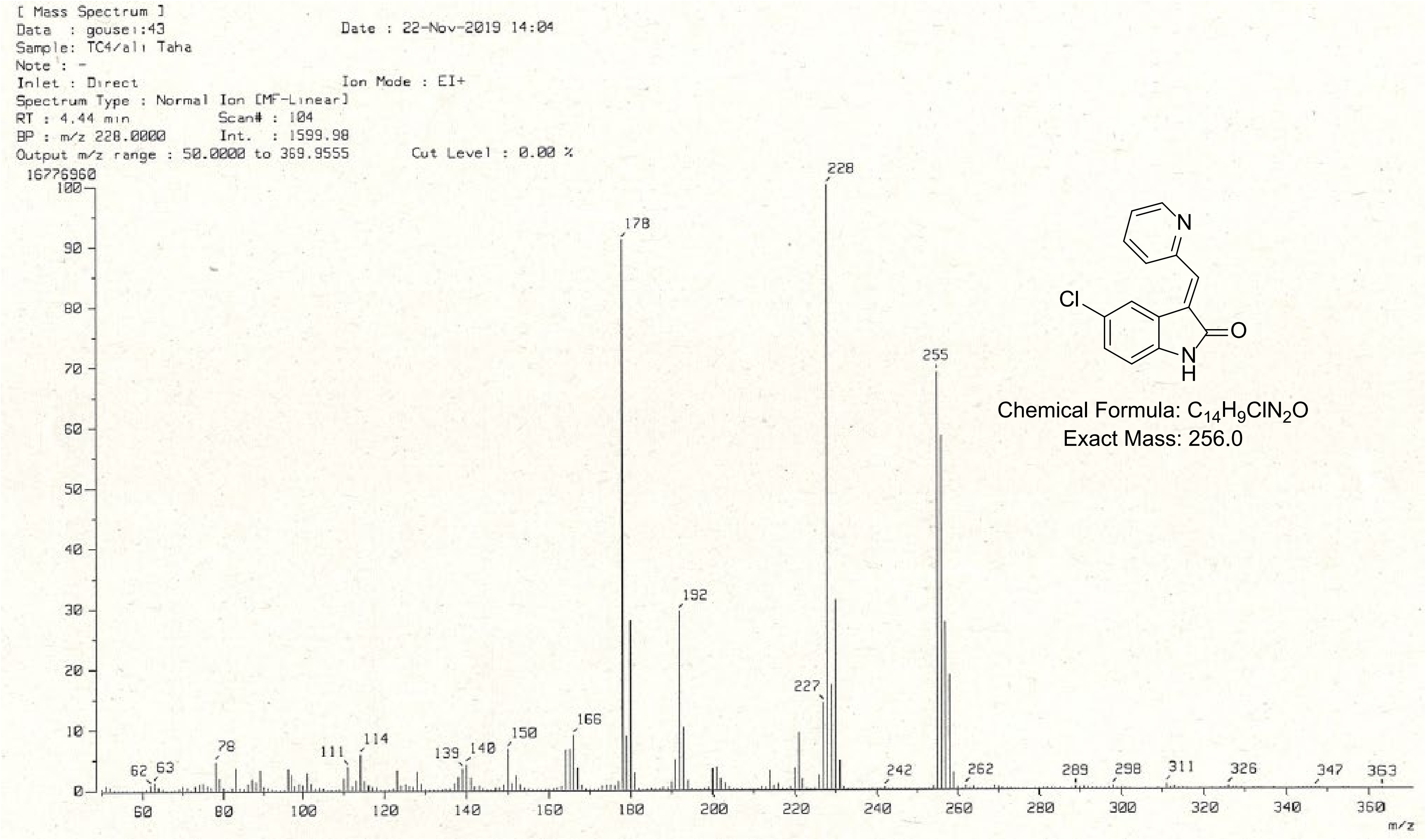

Figure S6. MS (EI+) of compound ***(E)*-2b**

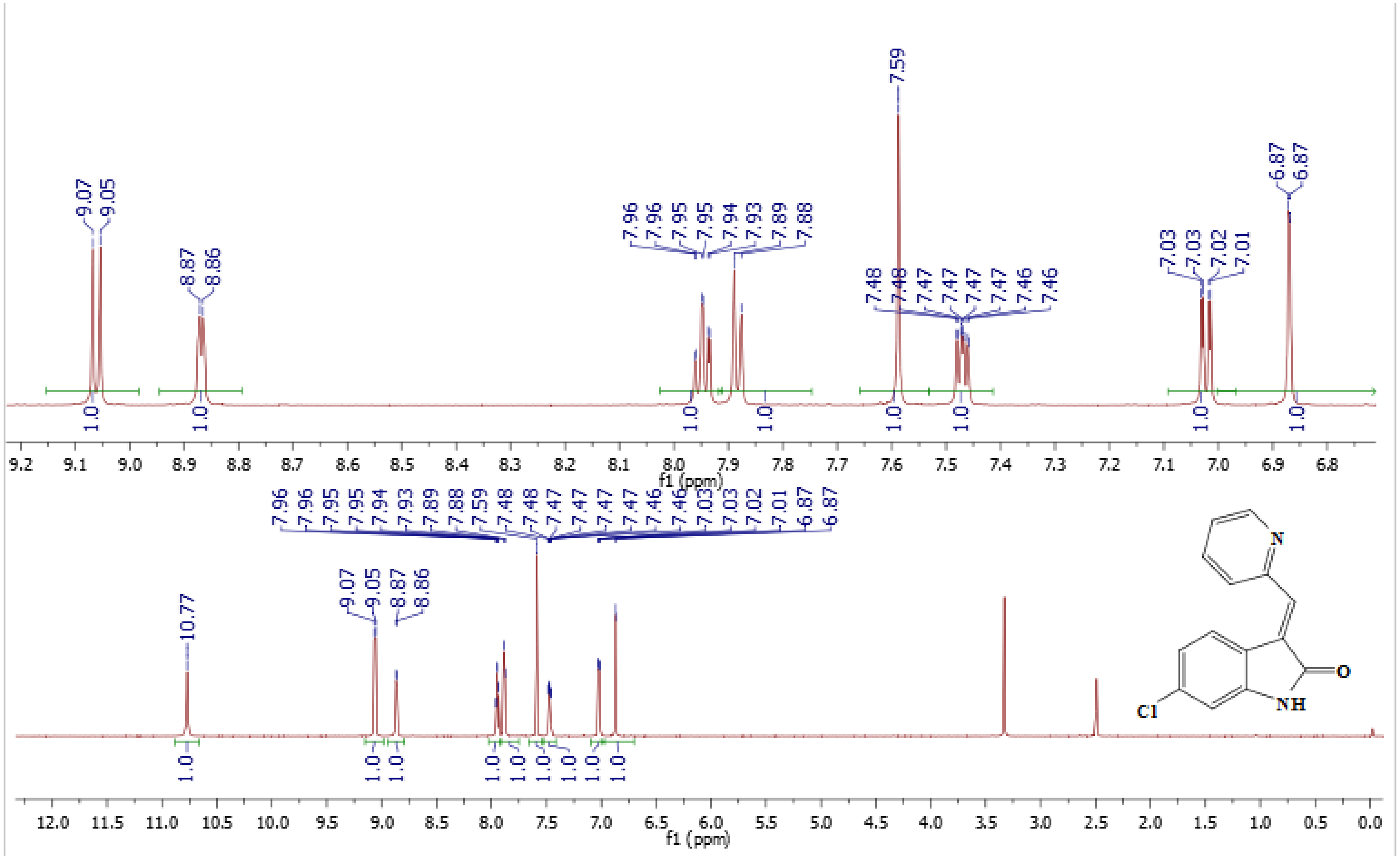

Figure S7. ^{1}H-NMR spectrum of compound **(*E*)-2c** (600MHz, DMSO-*d*6)

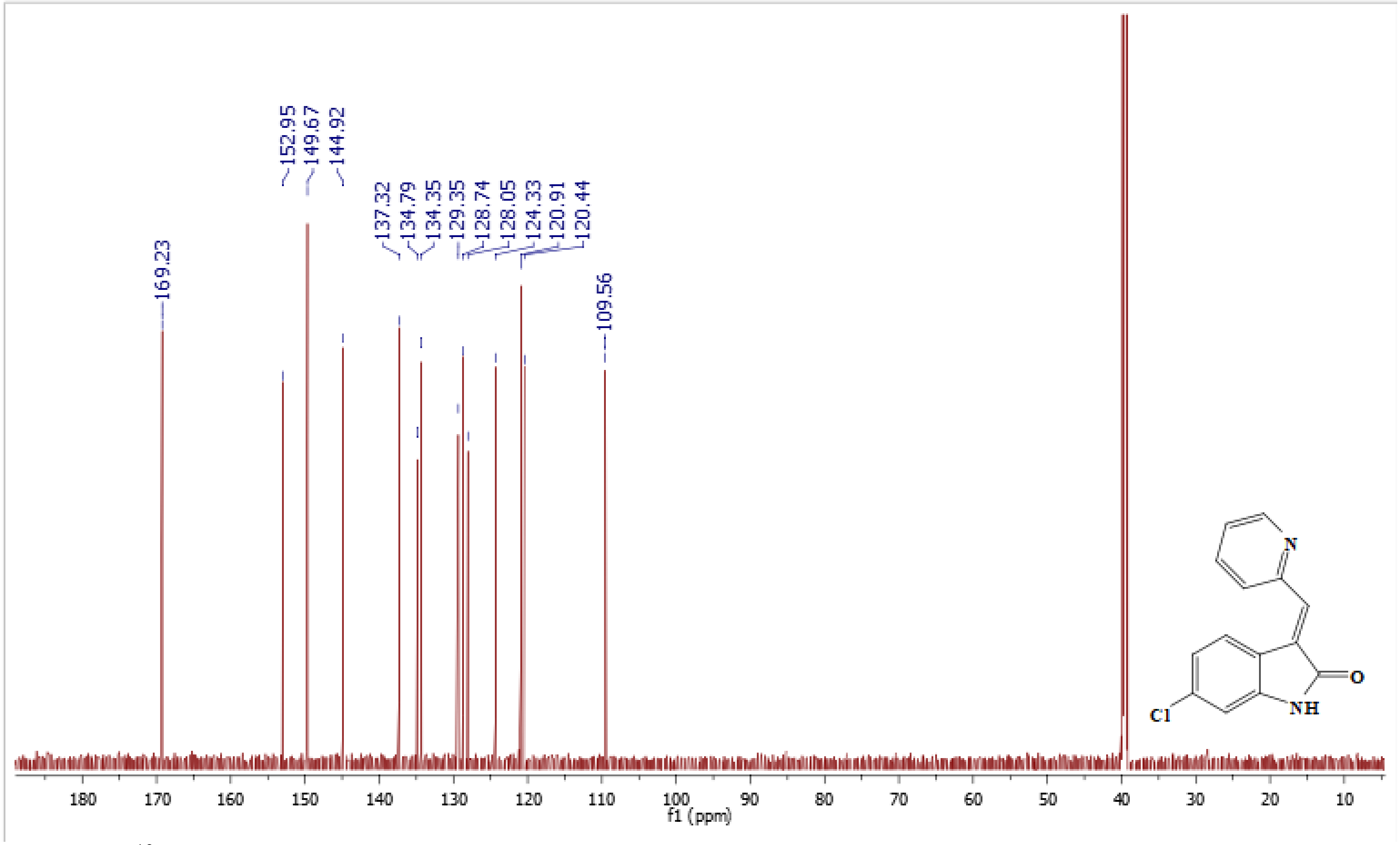

Figure S8. ^{13}C-NMR spectrum of compound ***(E)*-2c** (150MHz, DMSO-*d6*)

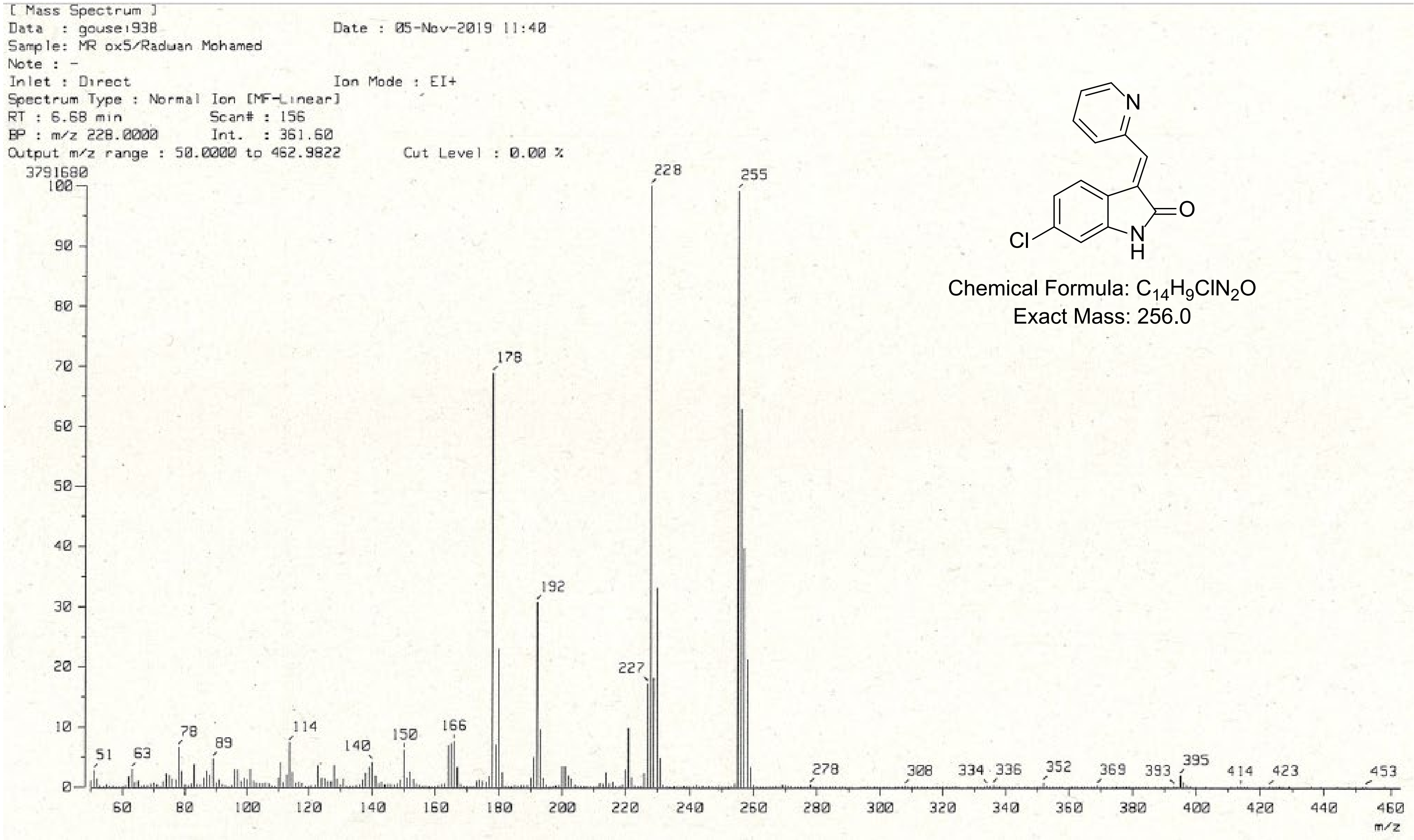

Figure S9. MS (EI+) of compound ***(E)*-2c**

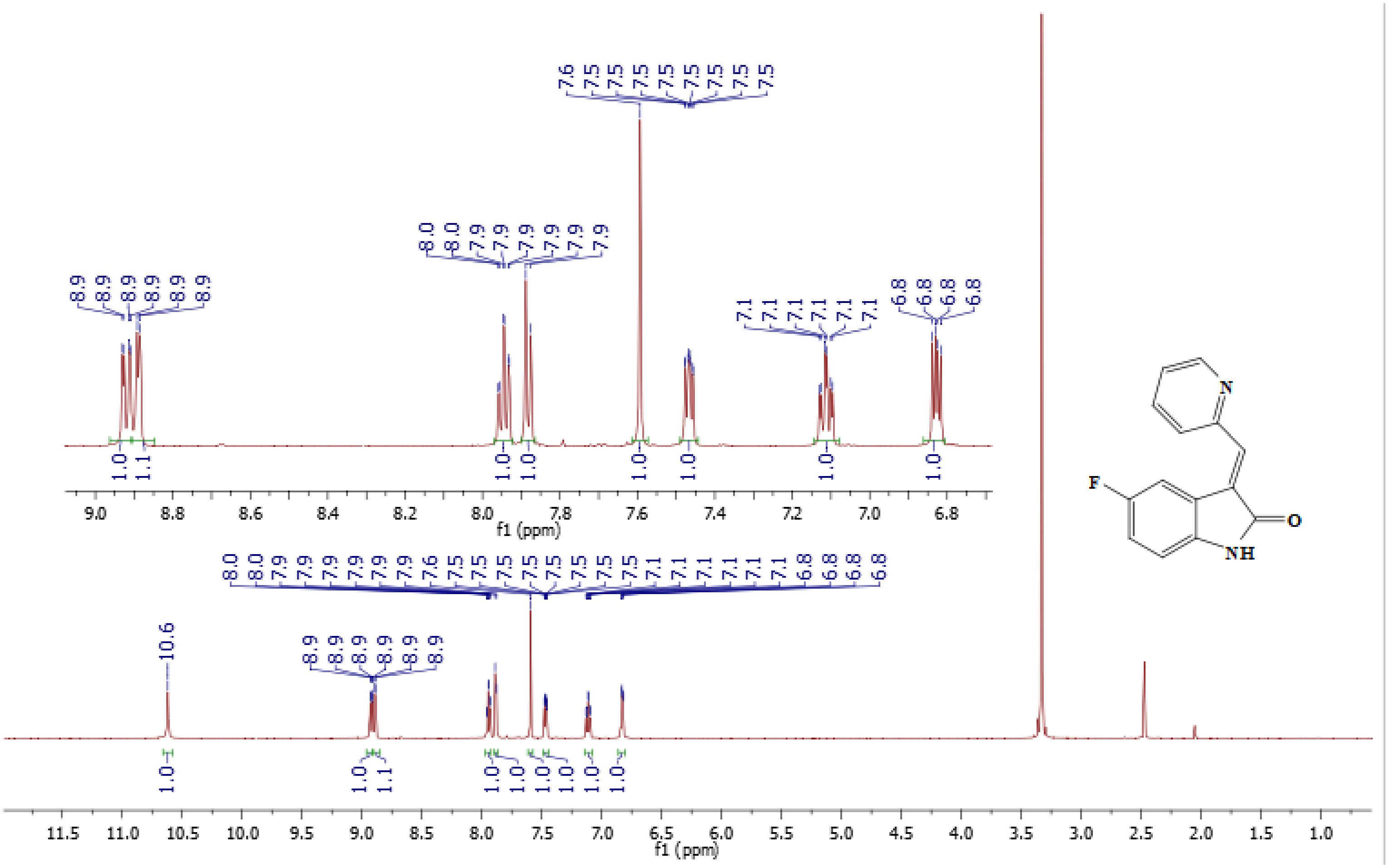

Figure S10. ^{1}H-NMR spectrum of compound ***(E)*-2d** (600MHz, DMSO-*d*6)

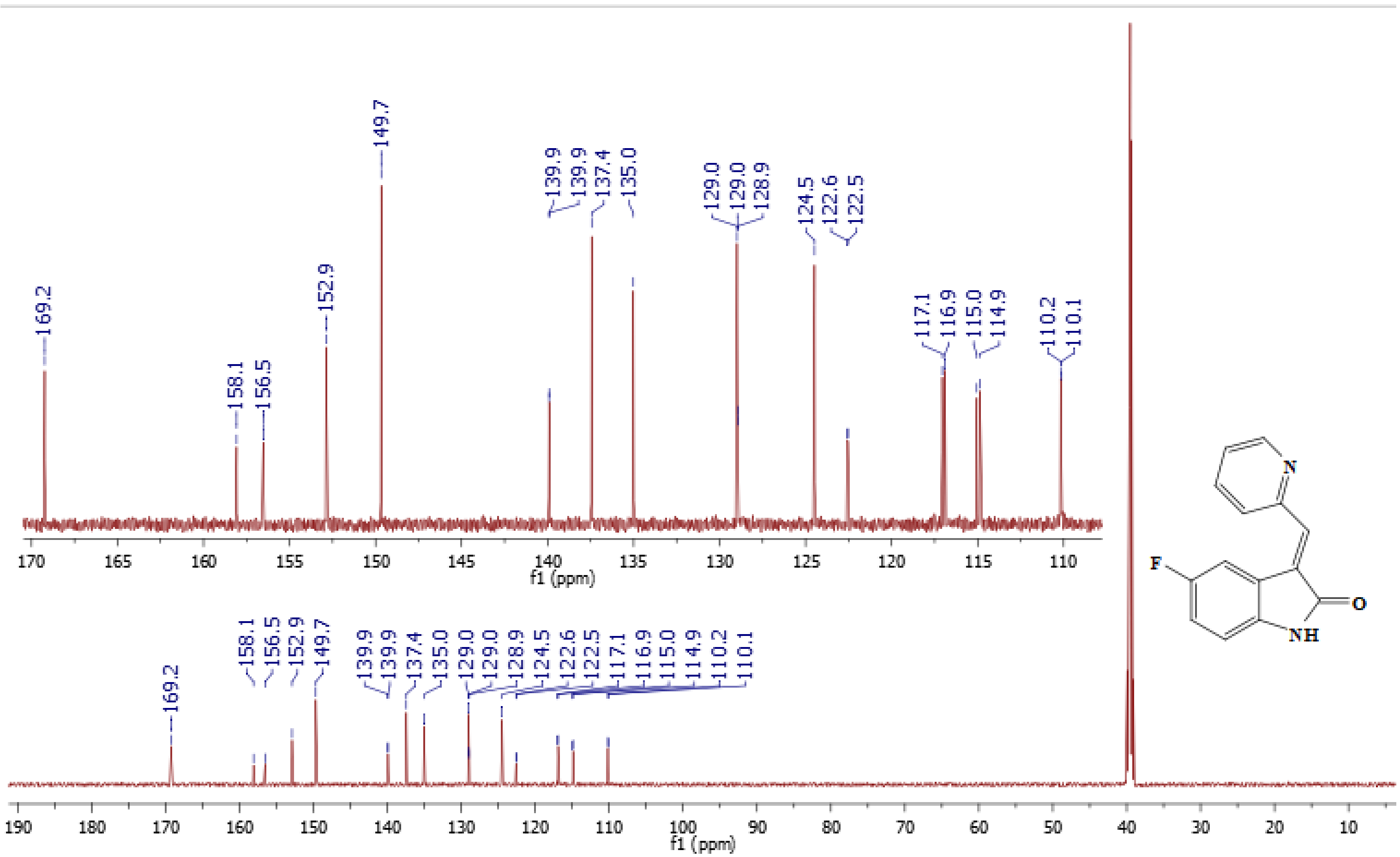

Figure S11. ^{13}C-NMR spectrum of compound ***(E)*-2d** (150MHz, DMSO-*d*6)

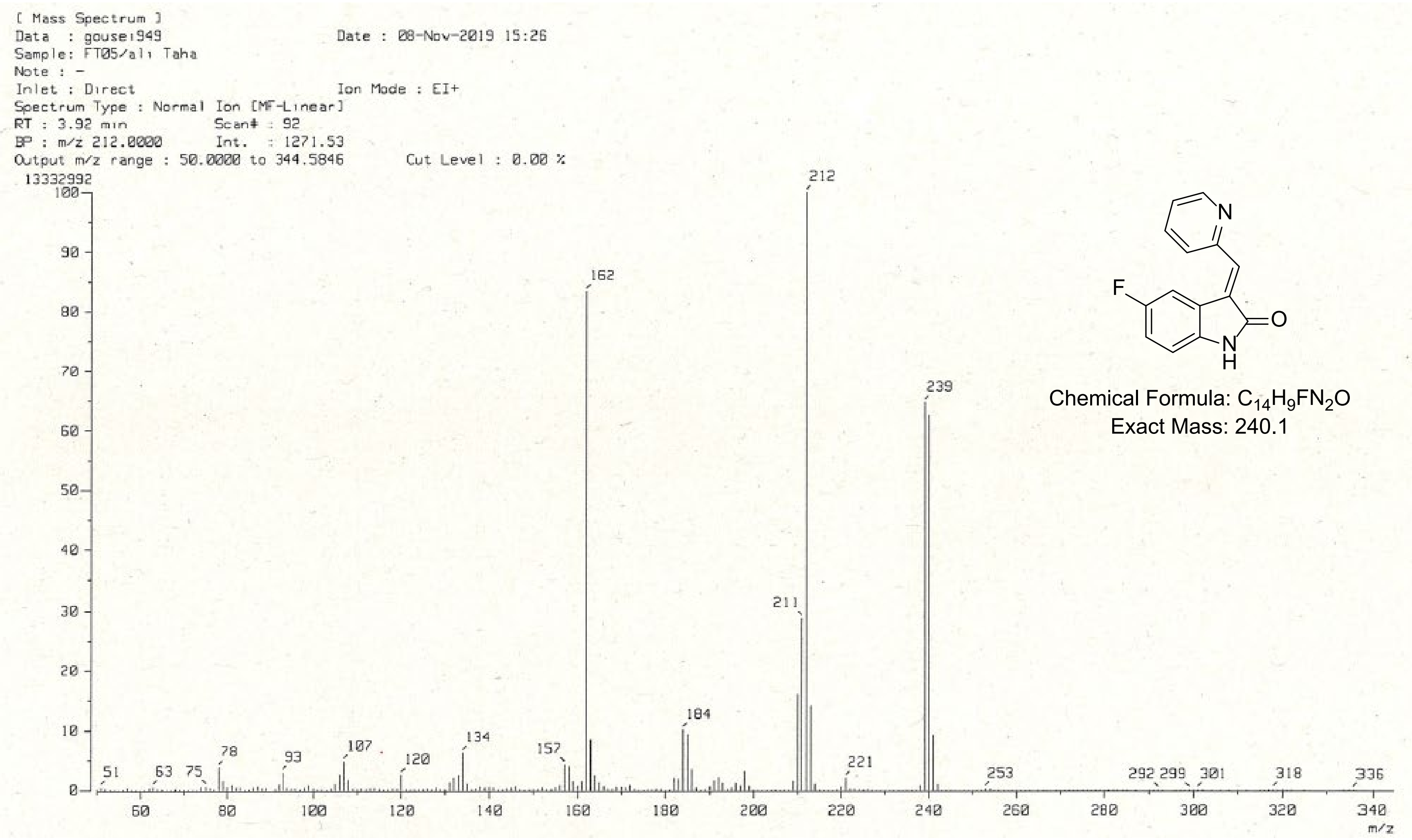

Figure S12. MS (EI+) of compound ***(E)*-2d**

[Elemental Composition] Page: 1

Data : gousei:02 Date : 18-Nov-2019 16:17

Sample: FT05/ali Taha

Note : -

Inlet : Direct Ion Mode : EI+

RT : 0.42 min Scan#: 13

Elements : C 100/0, H 100/0, O 2/0, N 3/1, F 2/0

Mass Tolerance : 10mmu

Unsaturation (U.S.) : -0.5 - 150.0

Observed m/z	Int%	Err[ppm / mmu]	U.S.	Composition
240.0675	68.4	-5.1 / -1.2	15.0	C 17 H 8 N 2
		-40.7 / -9.8	10.5	C 13 H 10 O 2 N 3
		-9.8 / -2.4	11.0	C 14 H 9 O N 2 F
		+21.1 / +5.1	11.5	C 15 H 8 N F 2
		-14.6 / -3.5	7.0	C 11 H 10 O 2 N 2 F 2
		+37.8 / +9.1	7.5	C 10 H 8 O 2 N 3 F 2

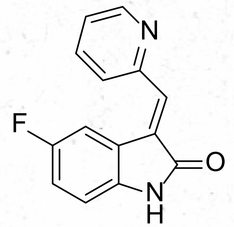

Chemical Formula: $C_{14}H_9FN_2O$
Exact Mass: 240.0699

Figure S13. HRMS (EI+) of compound ***(E)*-2d**

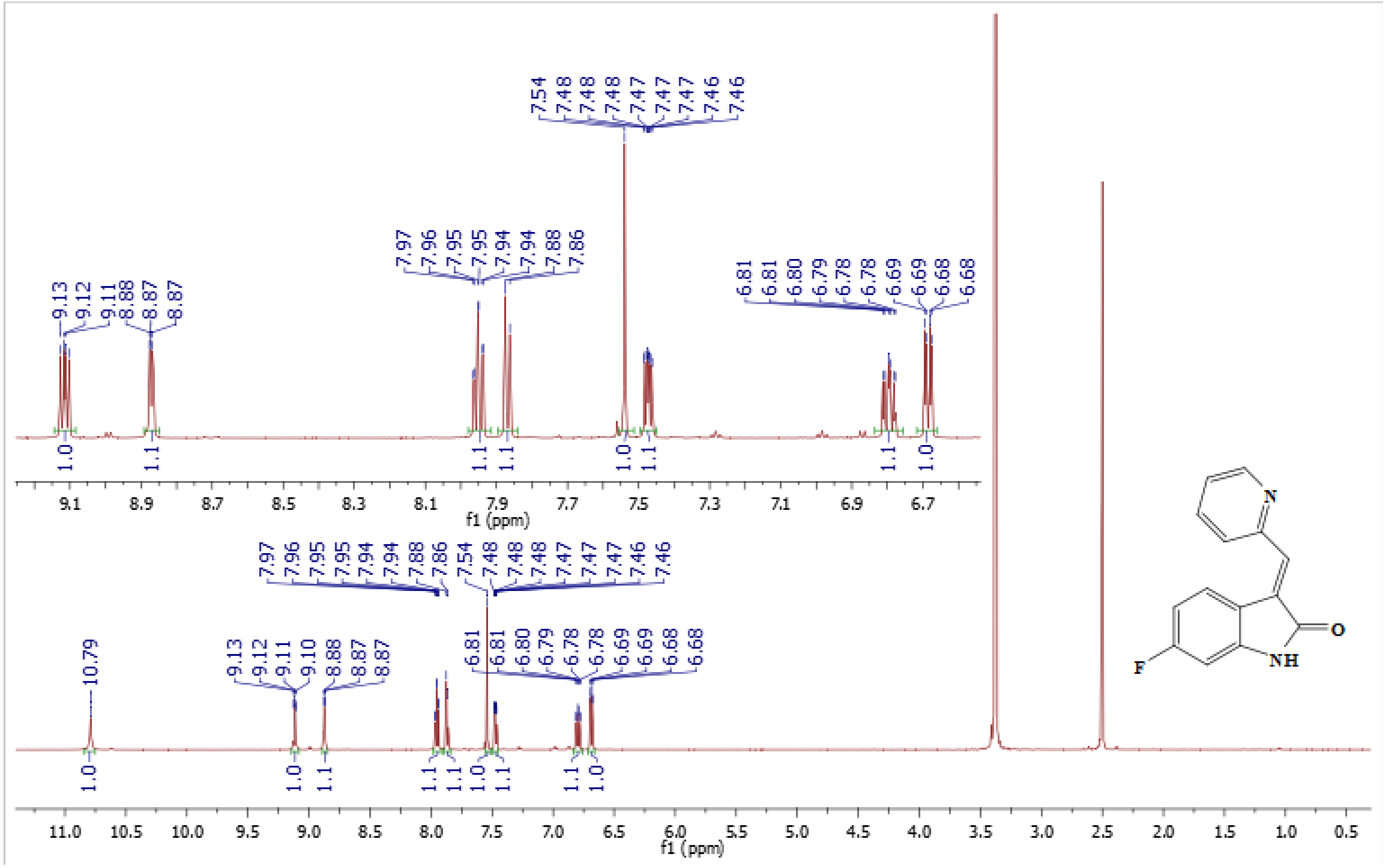

Figure S14. ^{1}H-NMR spectrum of compound **(*E*)-2e** (600MHz, DMSO-*d*6)

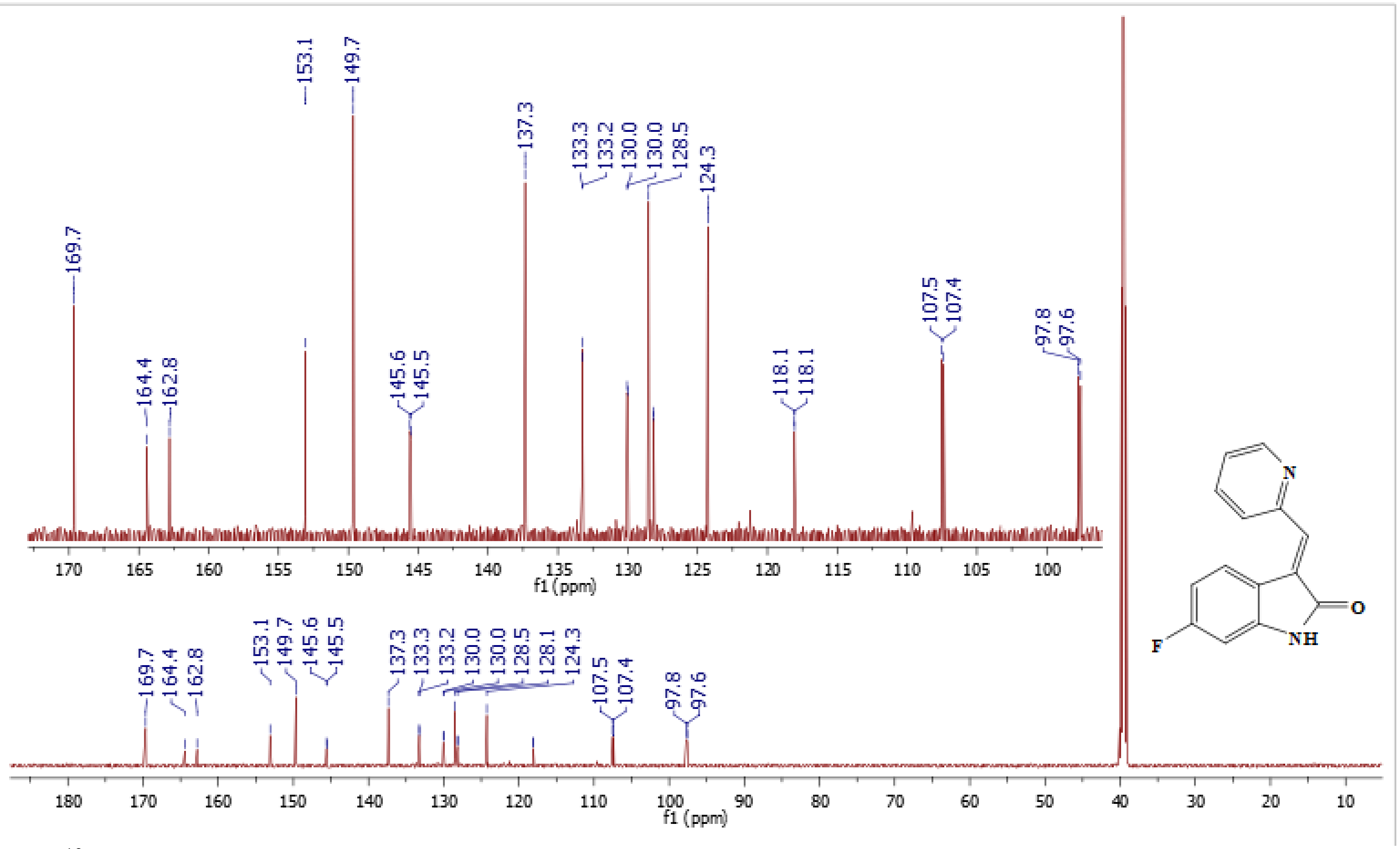

Figure S15. ^{13}C-NMR spectrum of compound **(*E*)-2e** (150MHz, DMSO-*d*6)

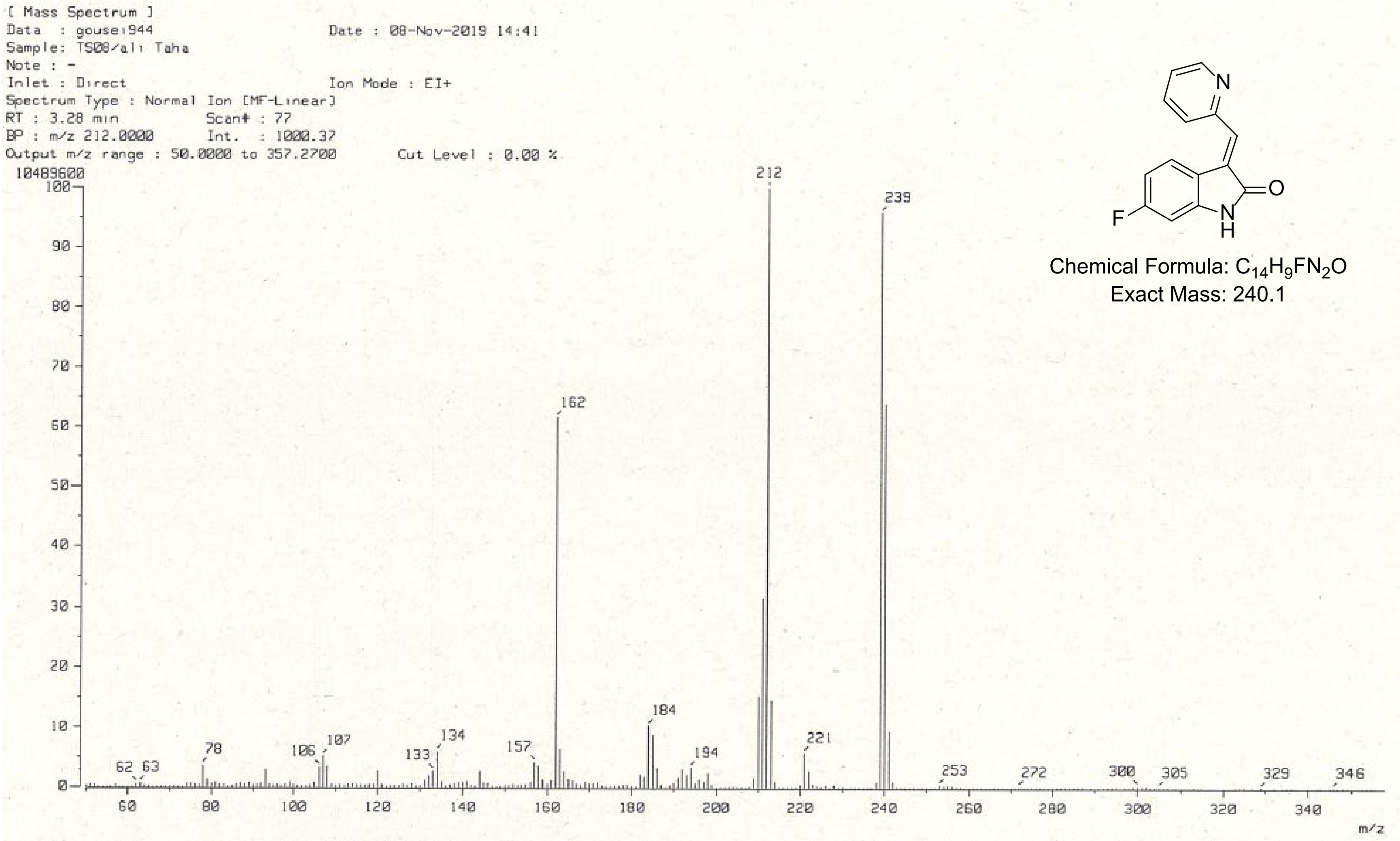

Figure S16. MS (EI+) of compound ***(E)*-2e**

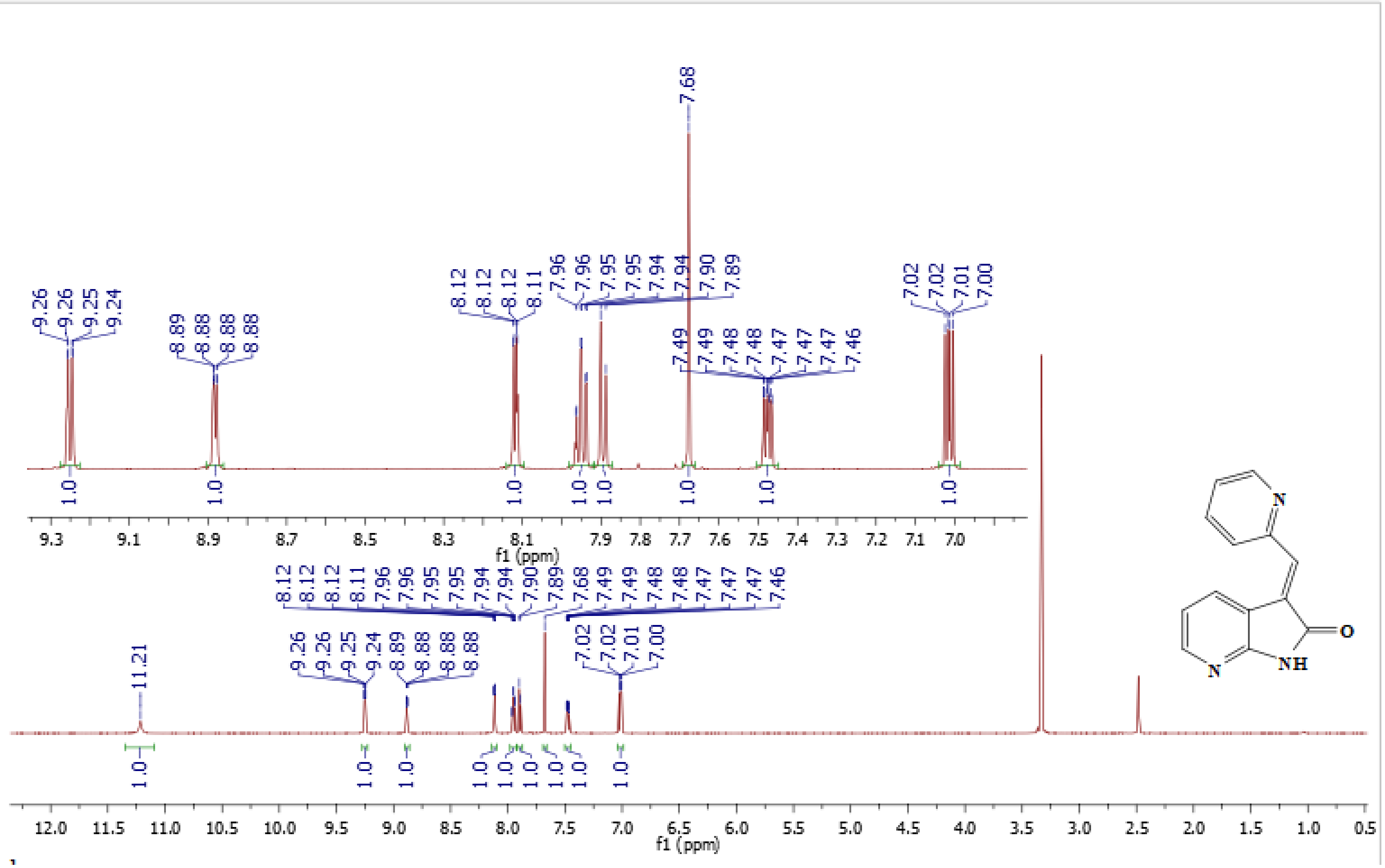

Figure S17. ^{1}H-NMR spectrum of compound ***(E)*-2f** (600MHz, DMSO-*d*6)

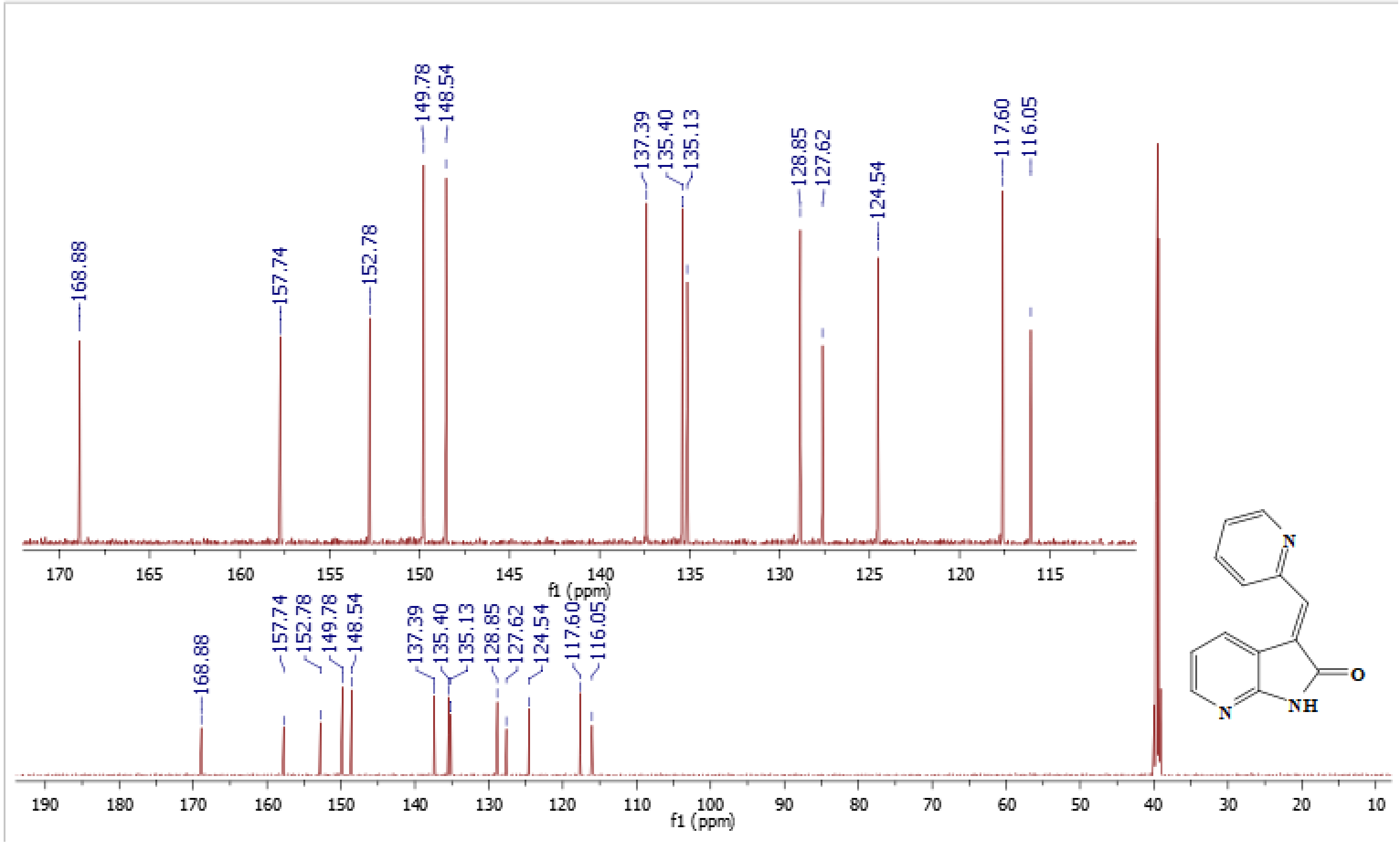

Figure S18. ^{13}C-NMR spectrum of compound **(*E*)-2f** (150MHz, DMSO-*d*6)

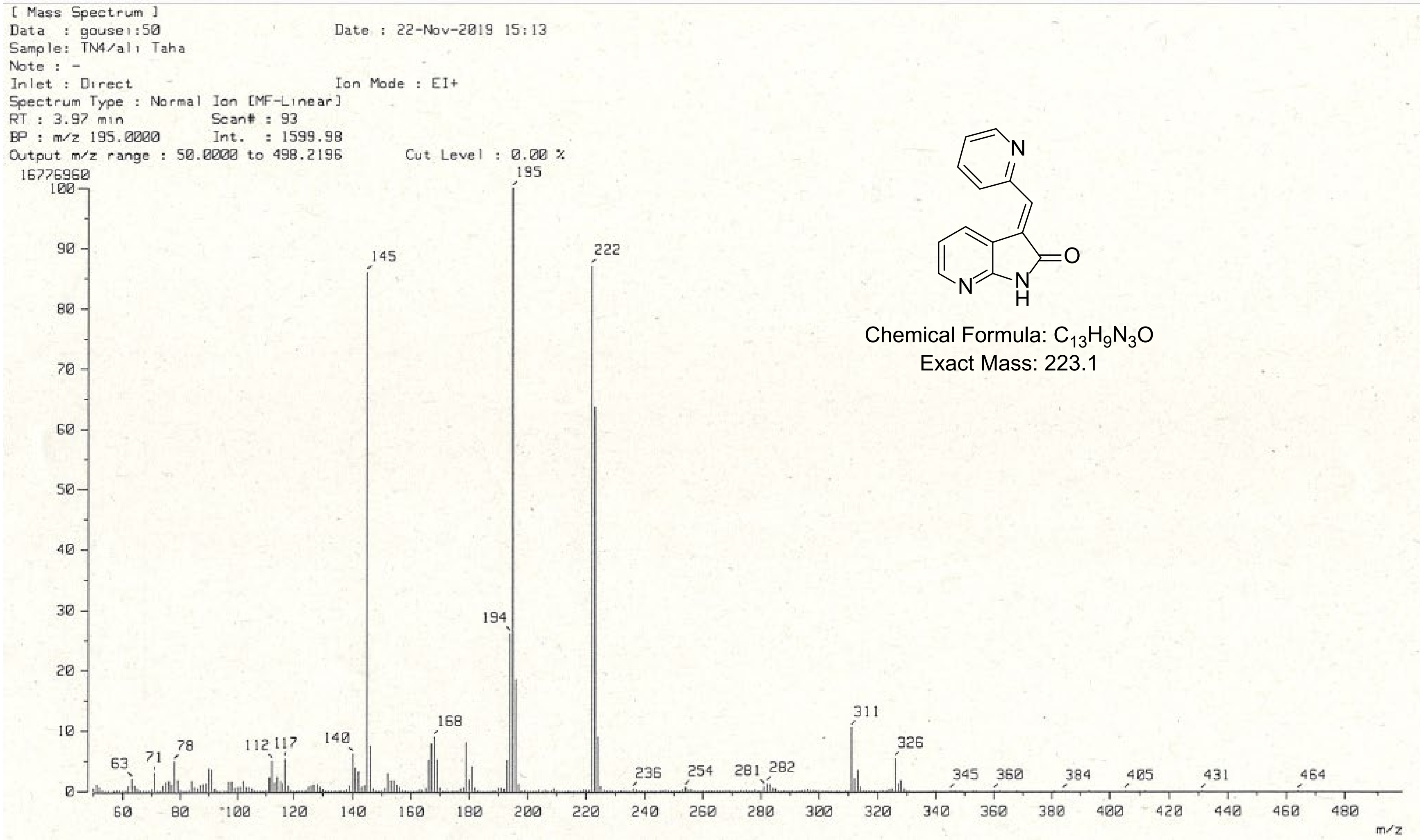

Figure S19. MS (EI+) of compound ***(E)*-2f**

[Elemental Composition] Page: 1

Data : gousei:51 Date : 22-Nov-2019 15:23

Sample: TN4/ali Taha

Note : -

Inlet : Direct Ion Mode : EI+

RT : 1.45 min Scan#: 41

Elements : C 100/0, H 100/0, O 5/0, N 5/0

Mass Tolerance : 10mmu

Unsaturation (U.S.) : -0.5 - 150.0

Observed m/z	Int%	Err[ppm / mmu]	U.S.	Composition
222.0647	59.1	-15.4 / -3.4	11.0	C 15 H 10 O 2
		+41.3 / +9.2	11.5	C 14 H 8 O 2 N
		+2.7 / +0.6	7.0	C 10 H 10 O 4 N 2
		-9.3 / -2.1	11.5	C 13 H 8 O N 3
		+8.8 / +2.0	7.5	C 8 H 8 O 3 N 5
223.0724	43.7	-15.7 / -3.5	10.5	C 15 H 11 O 2
		+40.7 / +9.1	11.0	C 14 H 9 O 2 N
		+2.3 / +0.5	6.5	C 10 H 11 O 4 N 2
		-9.7 / -2.2	11.0	C 13 H 9 O N 3
		+8.4 / +1.9	7.0	C 8 H 9 O 3 N 5

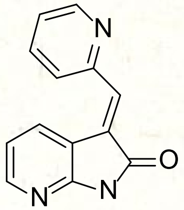

Chemical Formula: $C_{13}H_8N_3O$
Exact Mass: 222.0667

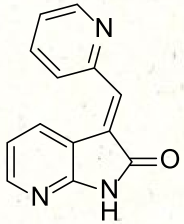

Chemical Formula: $C_{13}H_9N_3O$
Exact Mass: 223.0746

Figure S20. HRMS (EI+) of compound ***(E)*-2f**

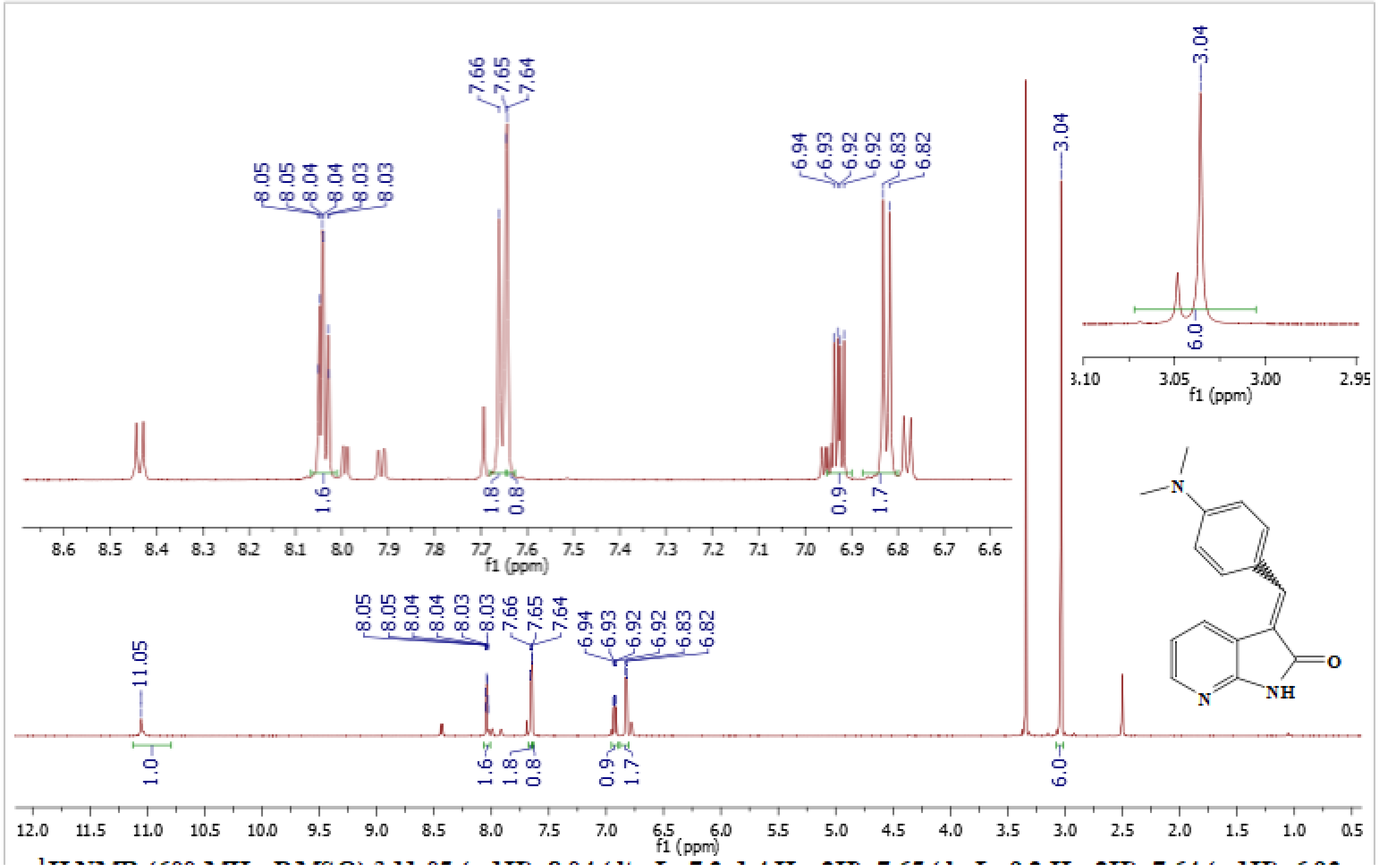

Figure S21. ^{1}H-NMR spectrum of compound ***(E/Z)*-3a** (600MHz, DMSO-*d*6)

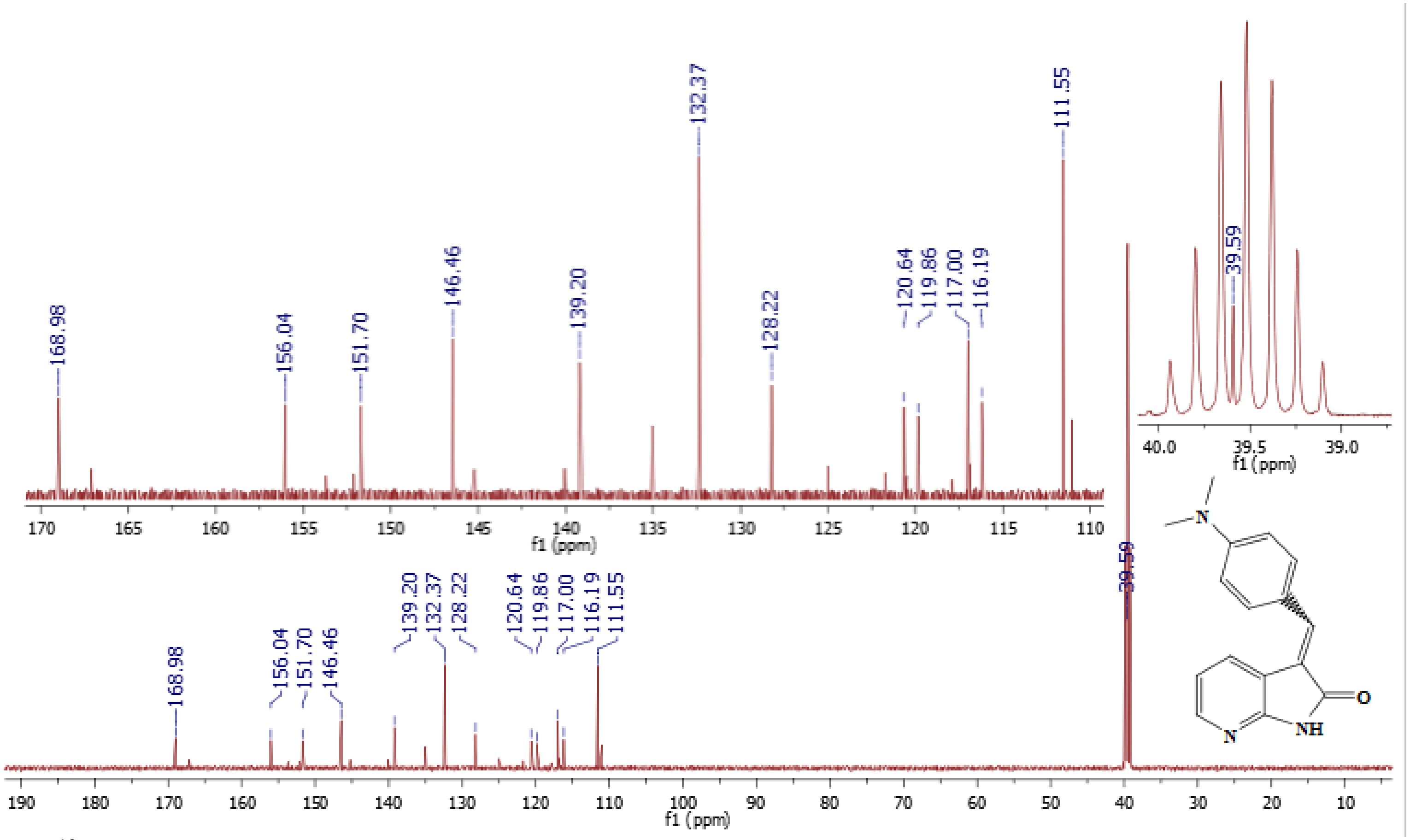

Figure S22. ^{13}C-NMR spectrum of compound **(*E*/*Z*)-3a** (150MHz, DMSO-*d*6)

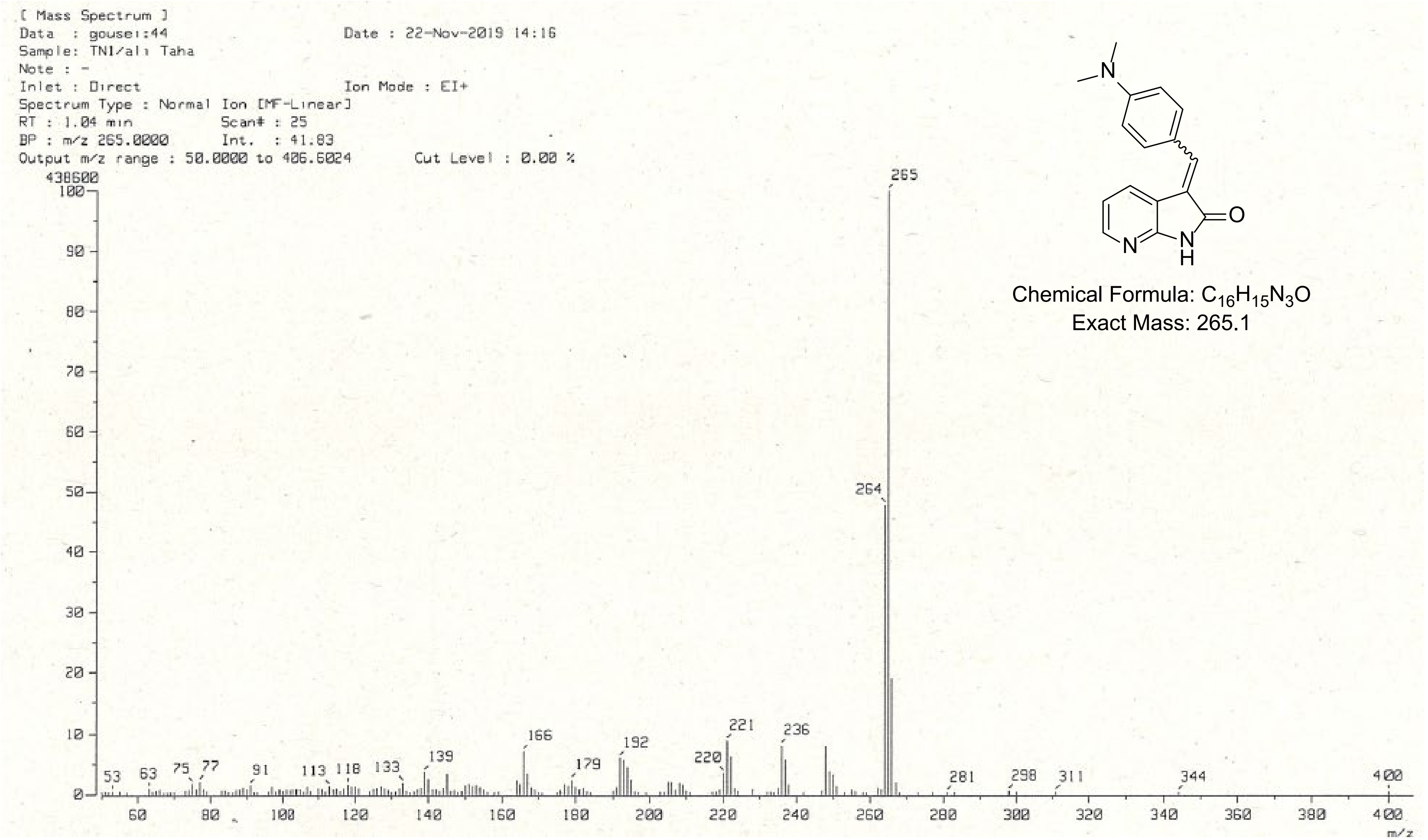

Figure S23. MS (EI+) of compound **(*E*/*Z*)-3a**

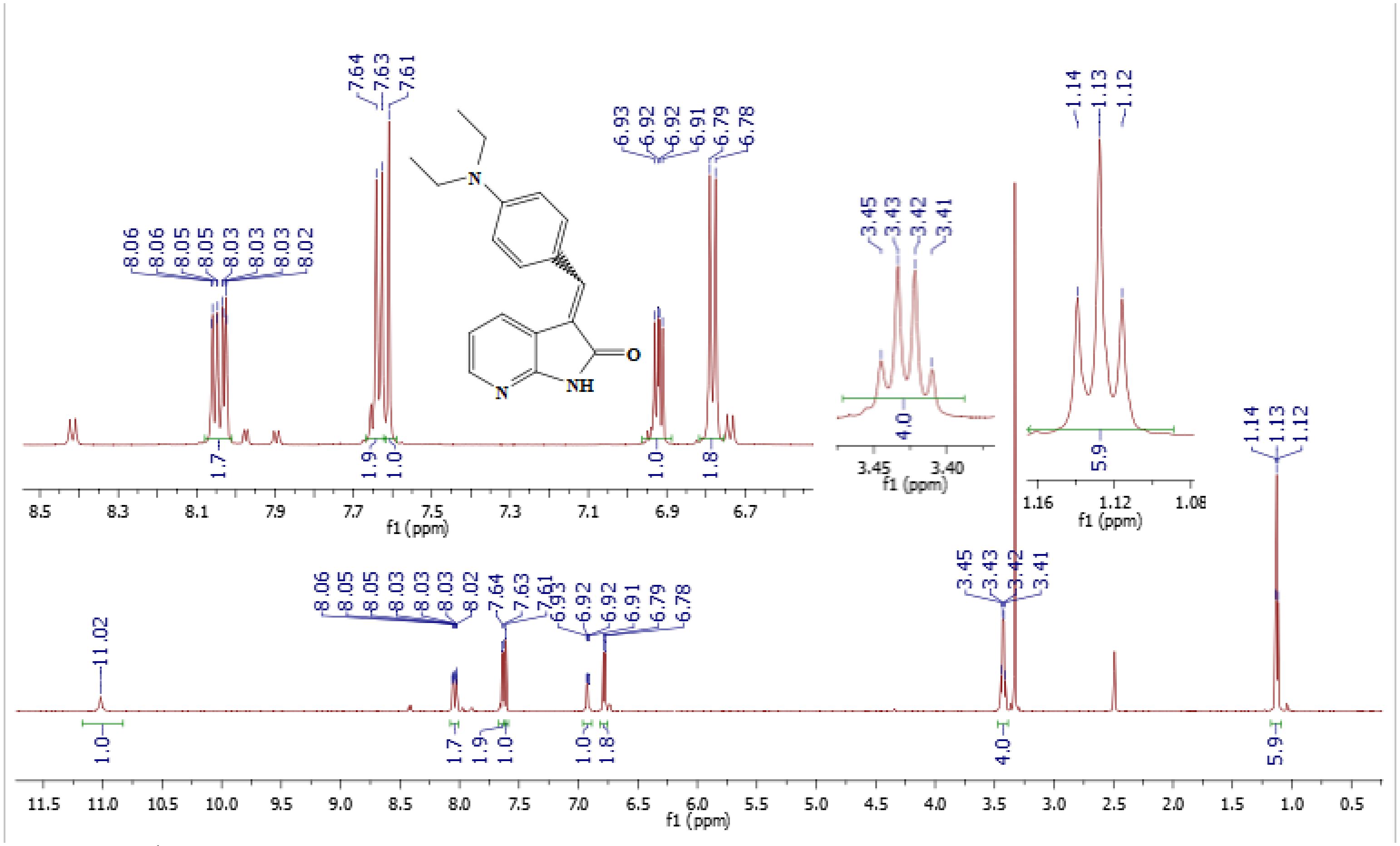

Figure S24. ^{1}H-NMR spectrum of compound ***(E/Z)*-3b** (600MHz, DMSO-*d*6)

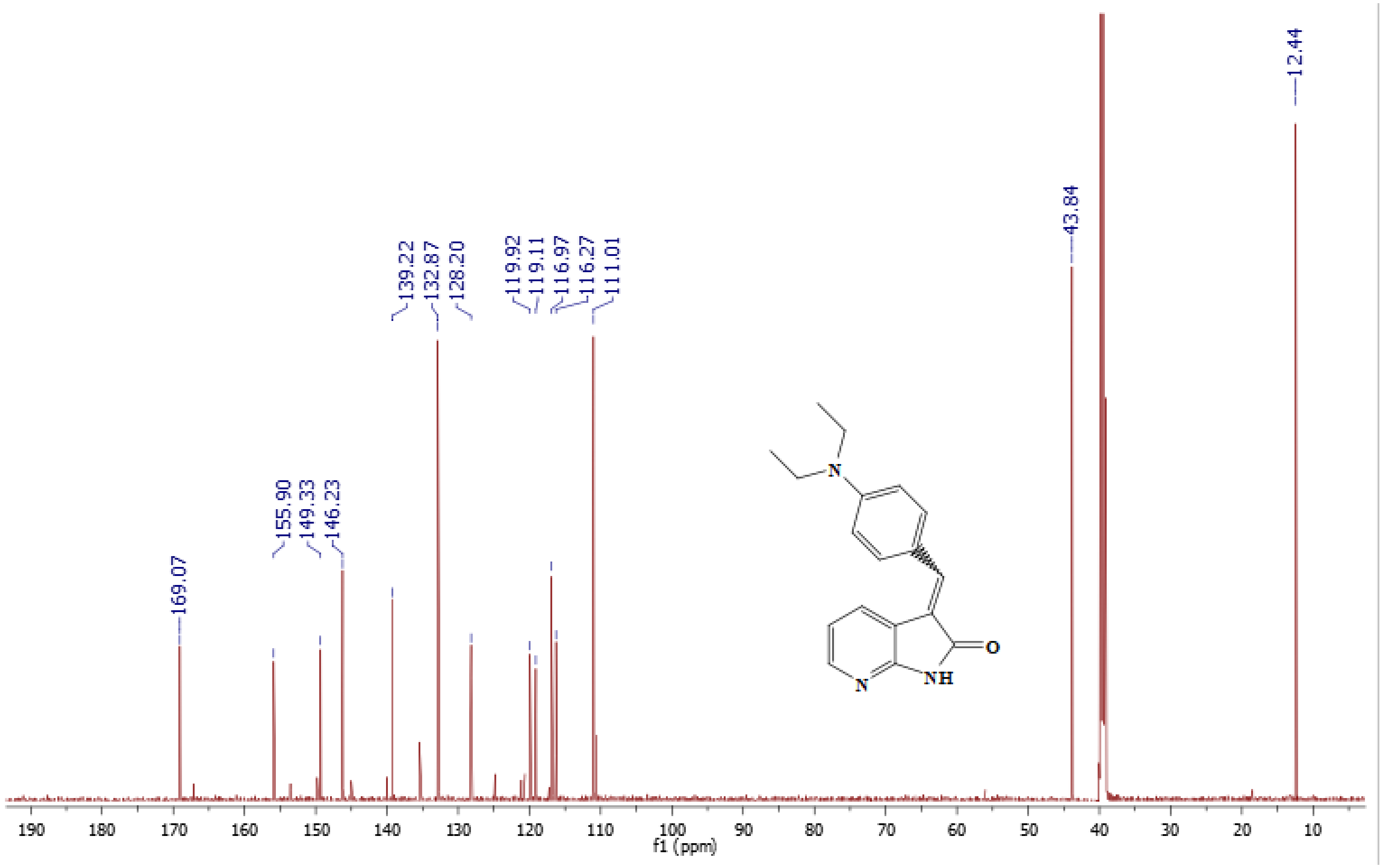

Figure S25. ^{13}C-NMR spectrum of compound **(*E*/*Z*)-3b** (150MHz, DMSO-*d*6)

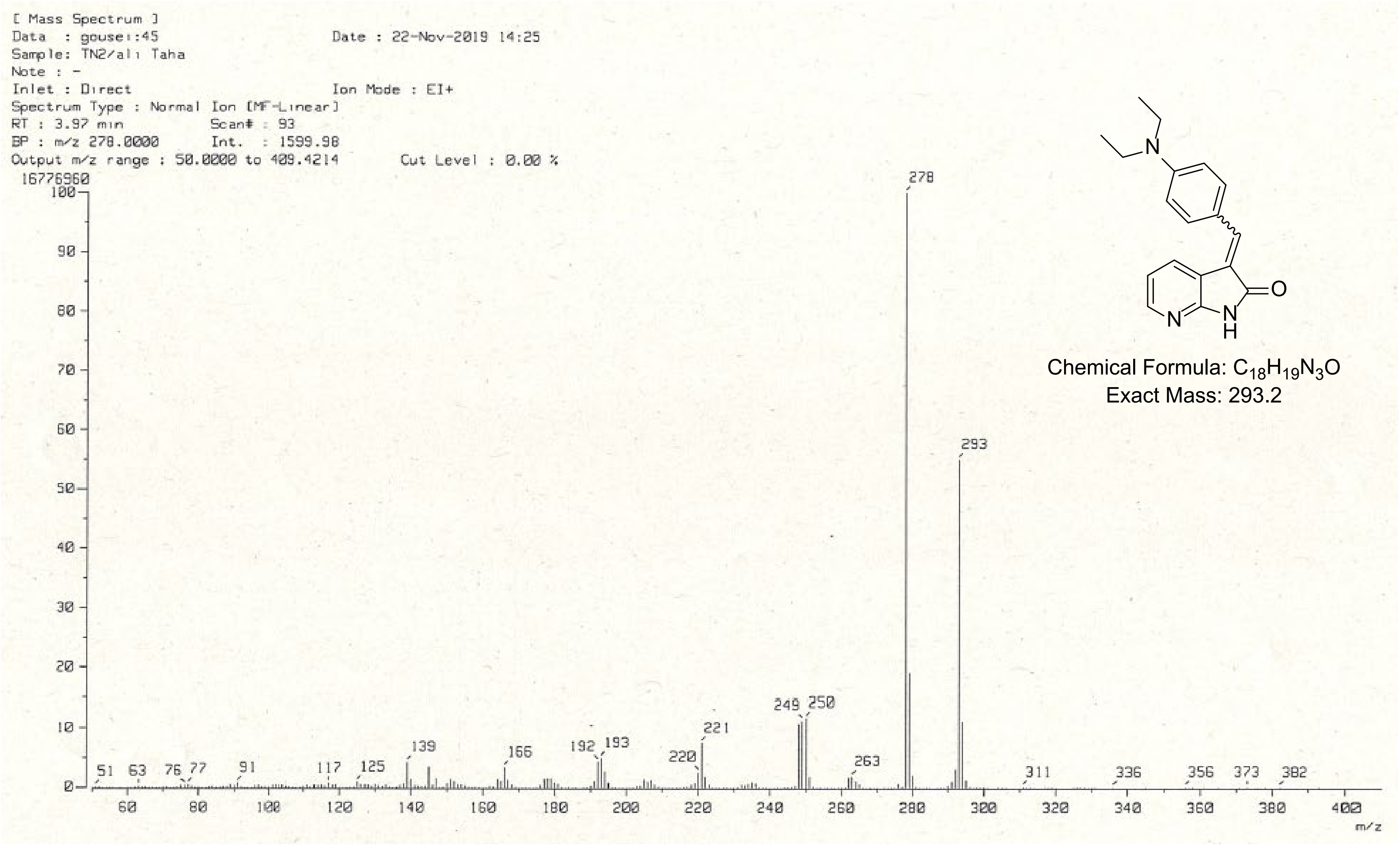

Figure S26. MS (EI+) of compound ***(E/Z)*-3b**

[Elemental Composition]

Page: 1

Data : gousei:46

Date : 22-Nov-2019 14:38

Sample: TN2/ali Taha

Note : -

Inlet : Direct

Ion Mode : EI+

RT : 1.20 min

Scan#: 38

Elements : C 100/0, H 100/0, O 3/0, N 5/0

Mass Tolerance : 10mmu

Unsaturation (U.S.) : -0.5 - 150.0

Observed m/z	Int%	Err[ppm / mmu]	U.S.	Composition
293.1517	52.8	-8.3 / -2.4	10.5	C 20 H 21 O 2
		-3.7 / -1.1	11.0	C 18 H 19 O N 3
		-32.9 / -9.6	6.5	C 14 H 21 O 3 N 4
		+10.0 / +2.9	7.0	C 13 H 19 O 3 N 5

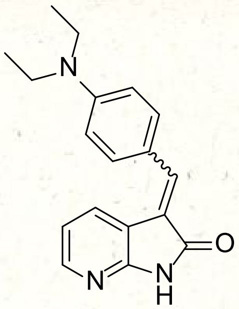

Chemical Formula: $C_{18}H_{19}N_3O$

Exact Mass: 293.1528

Figure S27. HRMS (EI+) of compound ***(E/Z)*-3b**

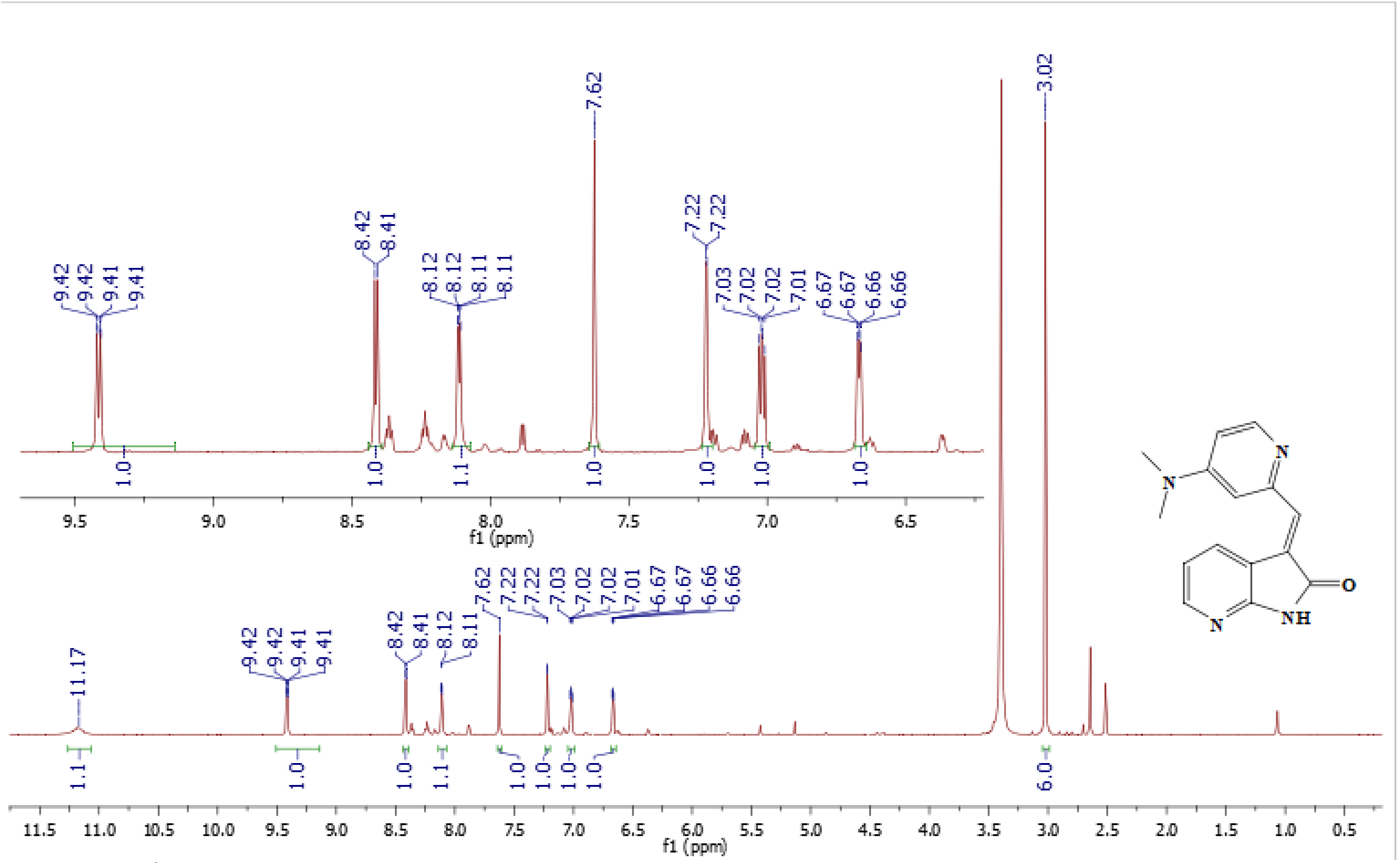

Figure S28. ^{1}H-NMR spectrum of compound ***(E)*-3c** (600MHz, DMSO-*d*6)

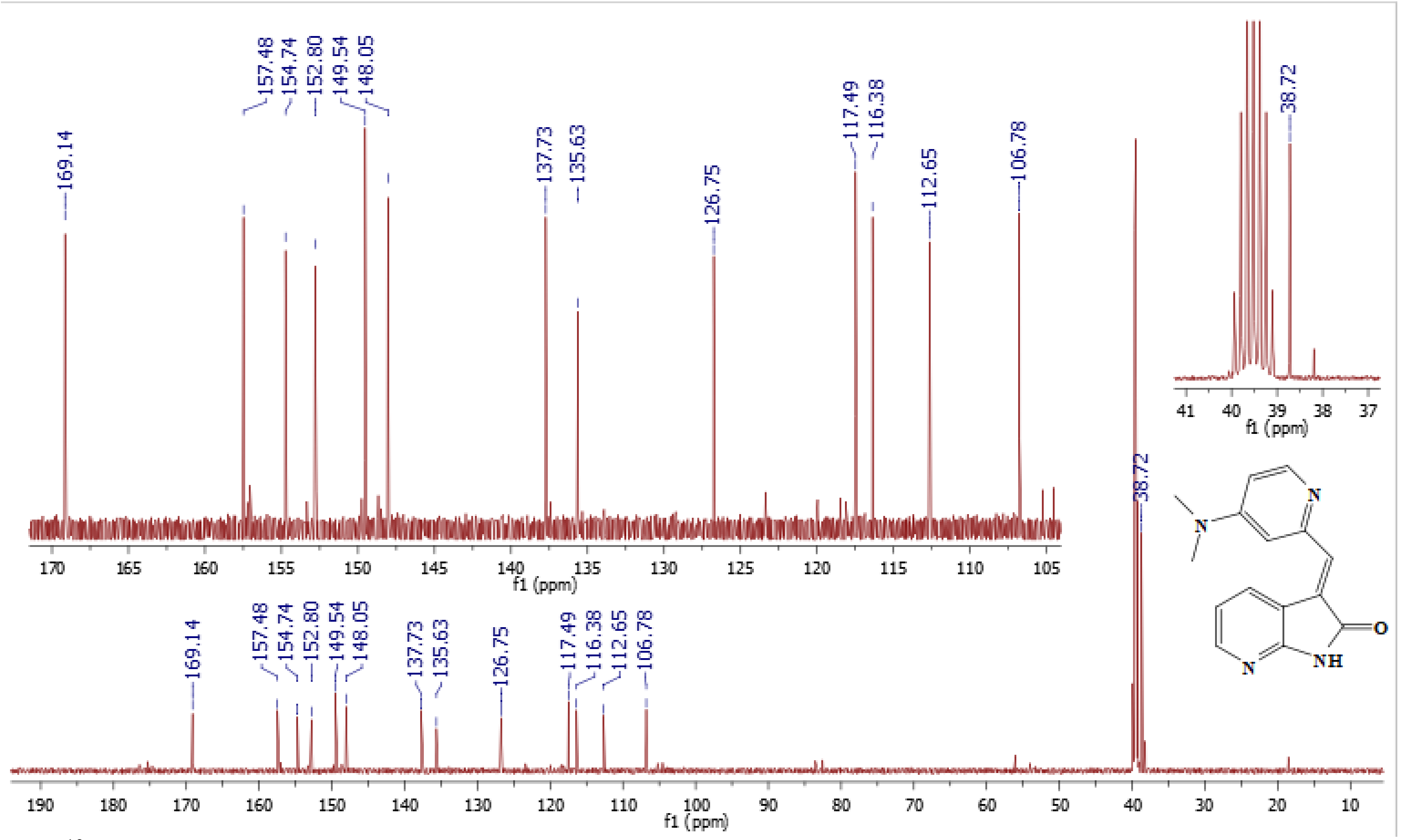

Figure S29. ^{13}C-NMR spectrum of compound **(*E*)-3c** (150MHz, DMSO-*d6*)

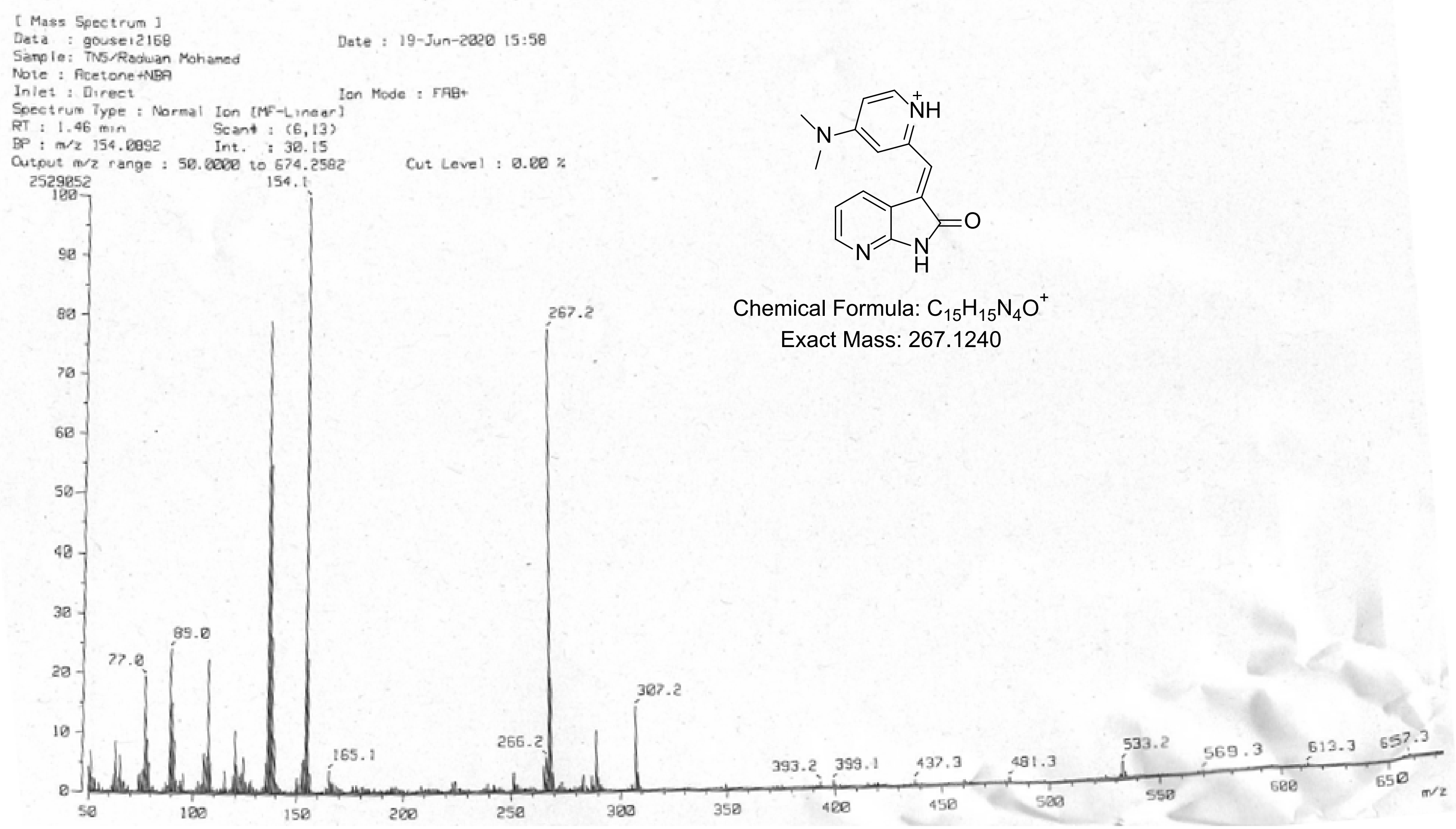

Figure S30. MS (FAB+) of compound ***(E)*-3c**

Page: 1

[Elemental Composition]

Data : gousei2169

Date : 19-Jun-2020 16:03

Sample: TN5/Radwan Mohamed

Note : Acetone+NBA

Inlet : Direct

Ion Mode : FAB+

RT : 5.13 min

Scan#: (19,24)

Elements : C 200/0, H 200/0, O 5/0, N 8/0

Mass Tolerance : 20ppm, 10mmu if m/z < 500, 20mmu if m/z > 1000

Unsaturation (U.S.) : -0.5 - 100.0

Observed m/z	Int%	Err [ppm / mmu]	U.S.	Composition
267.1268	100.0	+35.4 / +9.5	14.5	C 21 H 15
		+13.4 / +3.6	5.5	C 14 H 19 O 5
		+3.4 / +0.9	10.0	C 17 H 17 O 2 N
		-28.6 / -7.7	5.5	C 13 H 19 O 4 N 2
		+18.4 / +4.9	6.0	C 12 H 17 O 4 N 3
		+8.4 / +2.2	10.5	C 15 H 15 O N 4
		-23.6 / -6.3	6.0	C 11 H 17 O 3 N 5
		-33.6 / -9.0	10.5	C 14 H 15 N 6
		+23.5 / +6.3	6.5	C 10 H 15 O 3 N 6
		+13.4 / +3.6	11.0	C 13 H 13 N 7
		-8.6 / -2.3	2.0	C 6 H 17 O 5 N 7
		-18.6 / -5.0	6.5	C 9 H 15 O 2 N 8

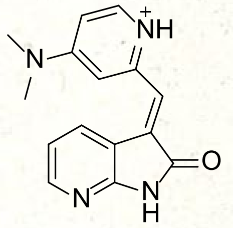

Chemical Formula: $C_{15}H_{15}N_4O^+$

Exact Mass: 267.1240

Figure S31. HRMS (FAB+) of compound ***(E)*-3c**

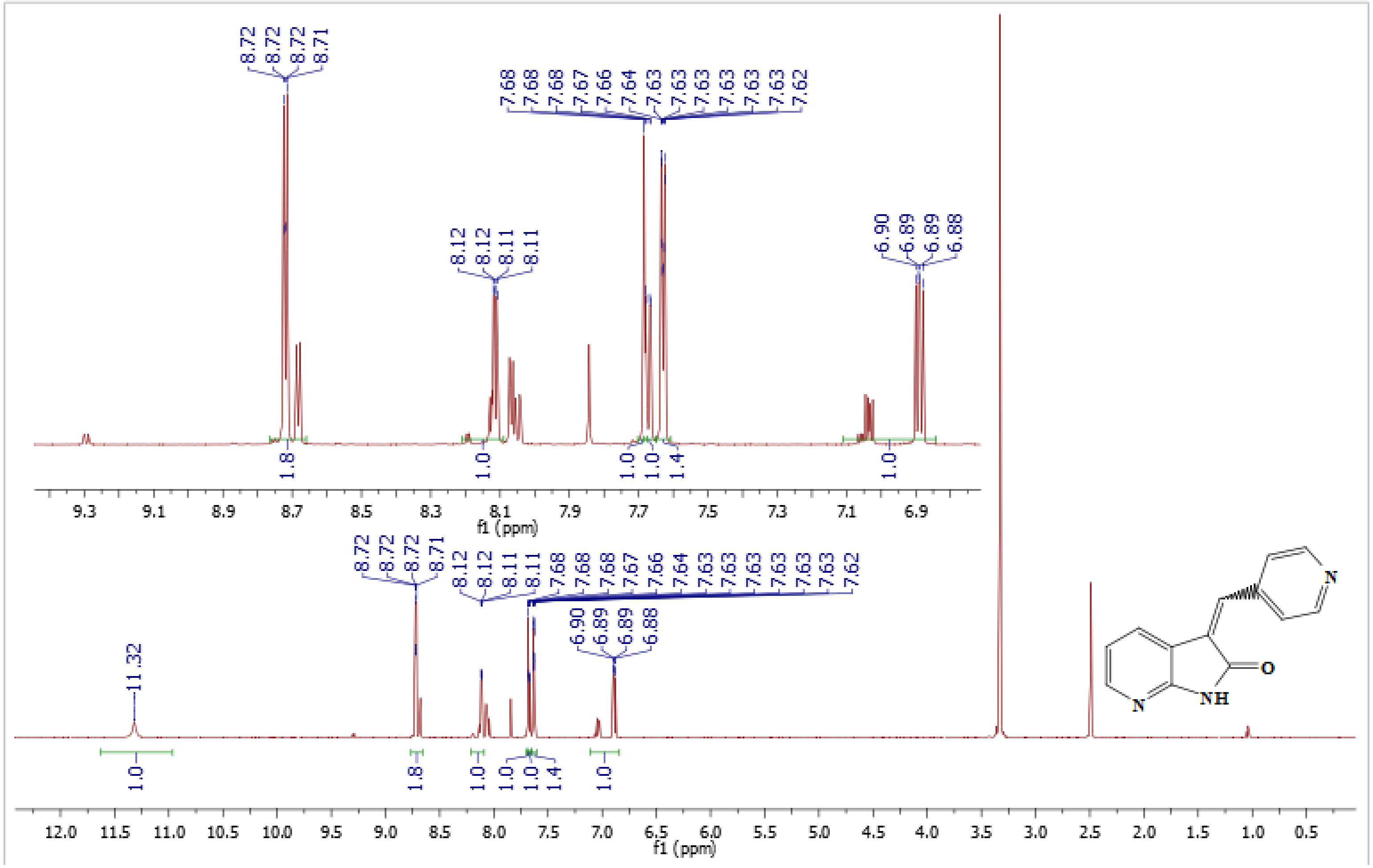

Figure S32. ^{1}H-NMR spectrum of compound ***(E/Z)*-3d** (600MHz, DMSO-*d*6)

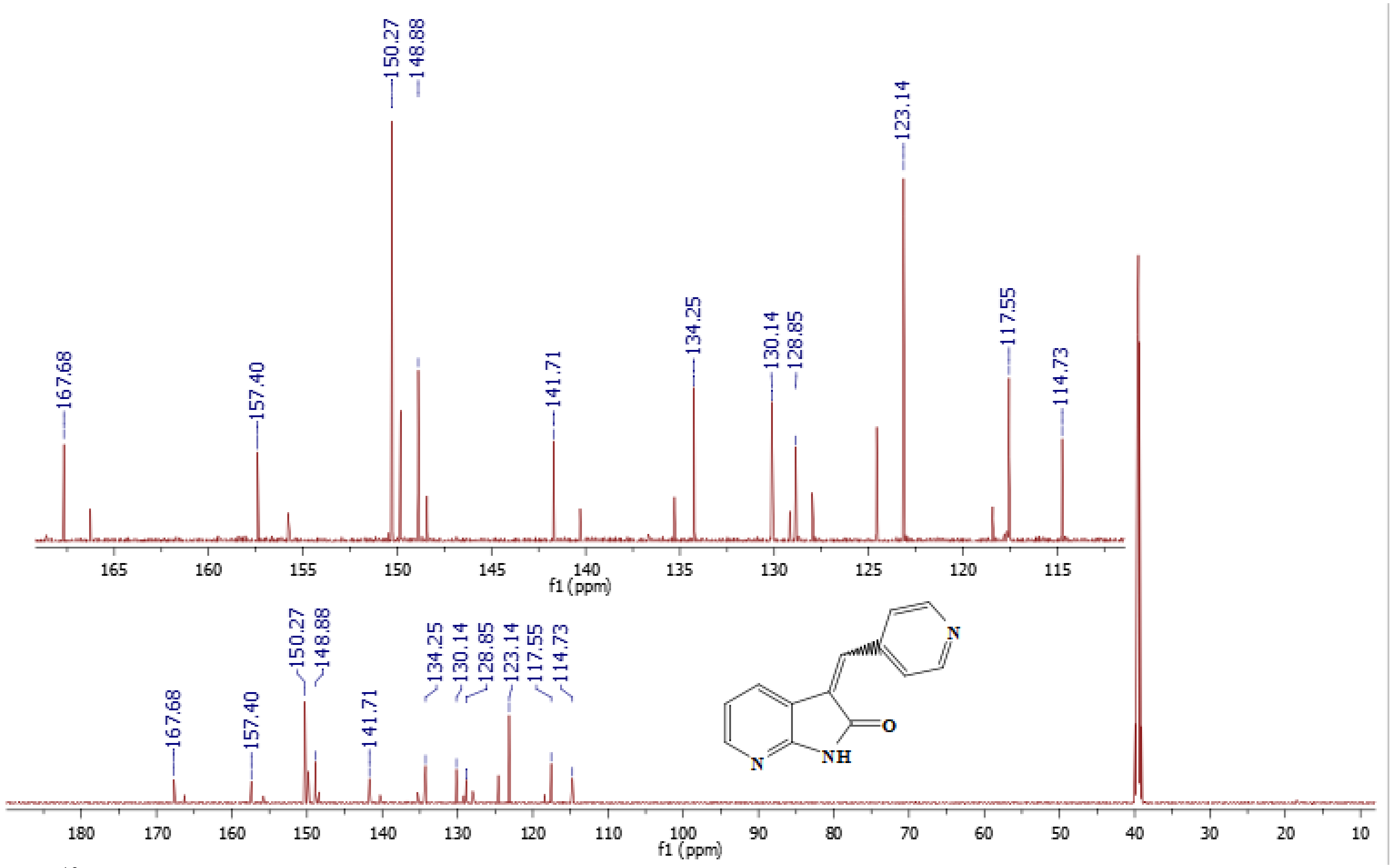

Figure S33. ^{13}C-NMR spectrum of compound ***(E/Z)*-3d** (150MHz, DMSO-*d*6)

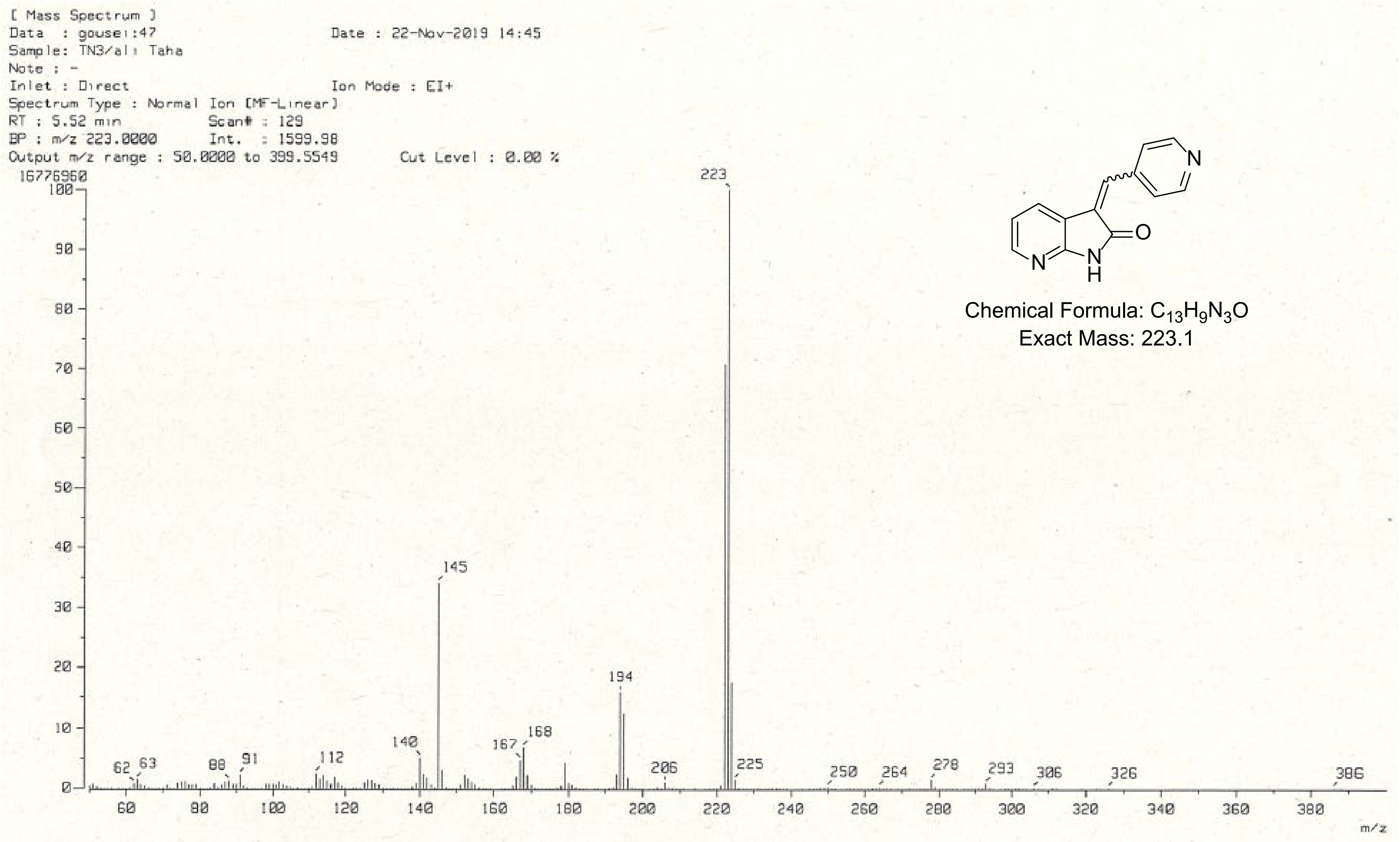

Figure S34. MS (EI+) of compound **(*E*/*Z*)-3d**

[Elemental Composition] Page: 1
Data : gousei:48 Date : 22-Nov-2019 14:56
Sample: TN3/ali Taha
Note : -
Inlet : Direct Ion Mode : EI+
RT : 0.87 min Scan#: 25
Elements : C 100/0, H 100/0, O 3/0, N 5/0
Mass Tolerance : 10mmu
Unsaturation (U.S.) : -0.5 - 150.0

Observed m/z	Int%	Err[ppm / mmu]	U.S.	Composition
223.0717	100.0	-18.8 / -4.2	10.5	C 15 H 11 O 2
		+37.5 / +8.4	11.0	C 14 H 9 O 2 N
		-12.8 / -2.9	11.0	C 13 H 9 O N 3
		+43.6 / +9.7	11.5	C 12 H 7 O N 4
		+5.2 / +1.2	7.0	C 8 H 9 O 3 N 5

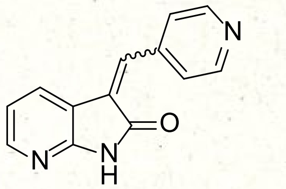

Chemical Formula: $C_{13}H_9N_3O$
Exact Mass: 223.0746

Figure S35. HRMS (EI+) of compound ***(E/Z)*-3d**

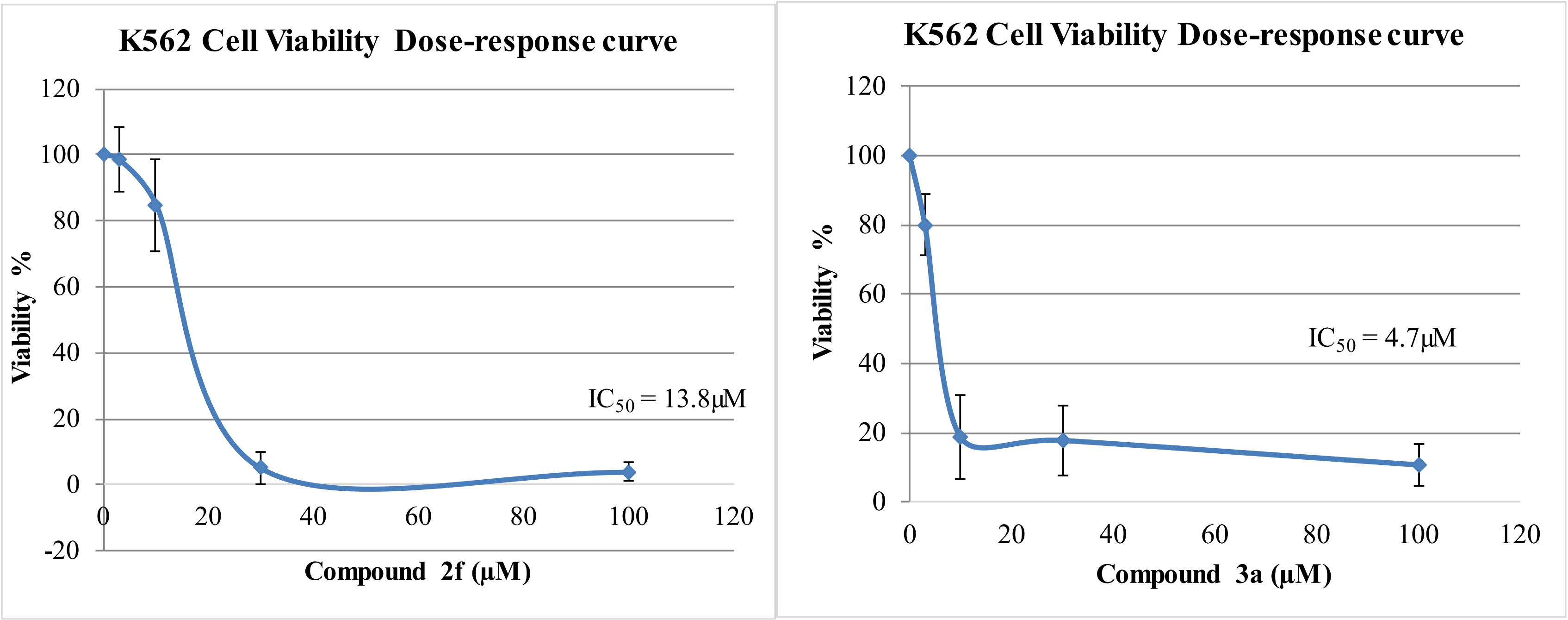

Figure S36. Cell viability Dose-response curve for inhibition of K562 cells. Data are given as mean ± SEM of n= 3 independent experiments, shown as percentage of control, DMSO (= 100%).

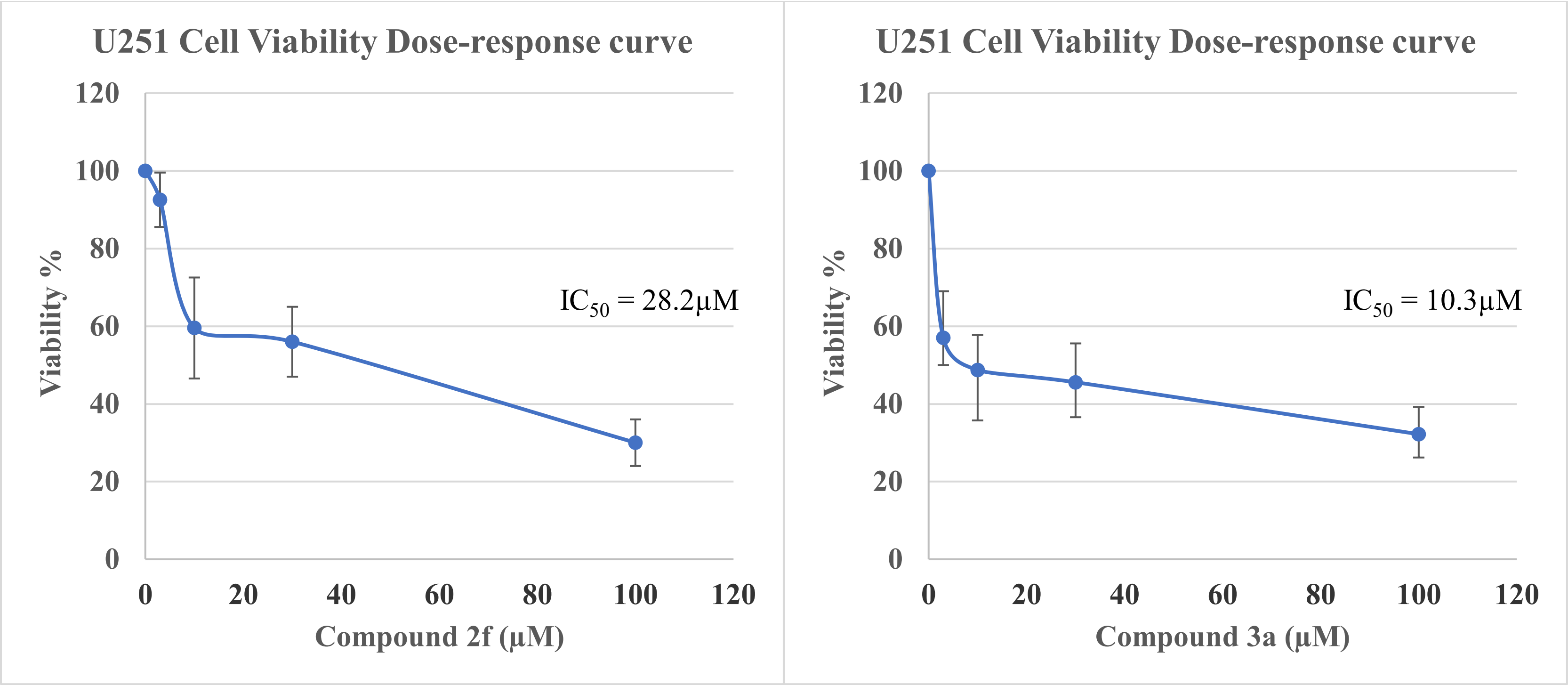

Figure S37. Cell viability Dose-response curve for inhibition of U251 cells. Data are given as mean ± SEM of n= 3 independent experiments, shown as percentage of control, DMSO (= 100%).

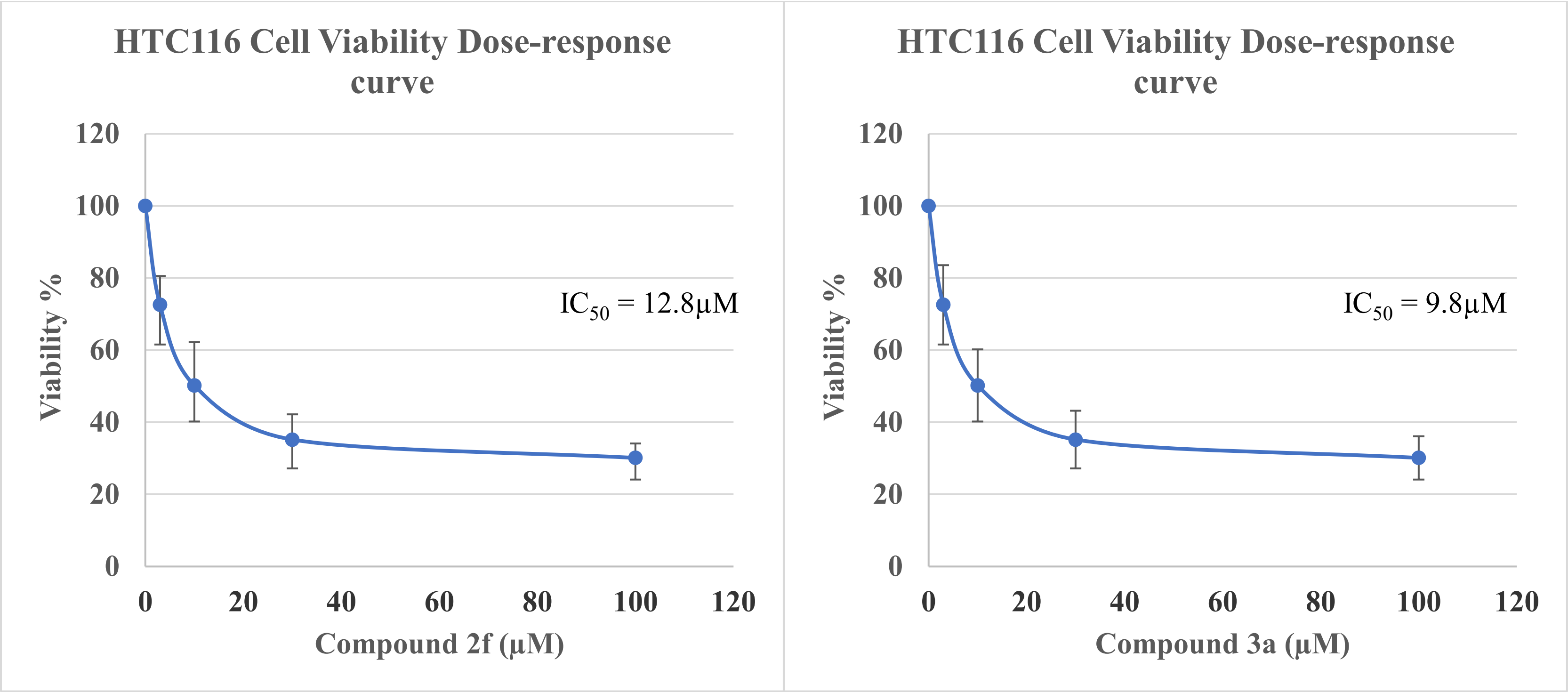

Figure S38. Cell viability Dose-response curve for inhibition of HTC116 cells. Data are given as mean ± SEM of n= 3 independent experiments, shown as percentage of control, DMSO (= 100%).

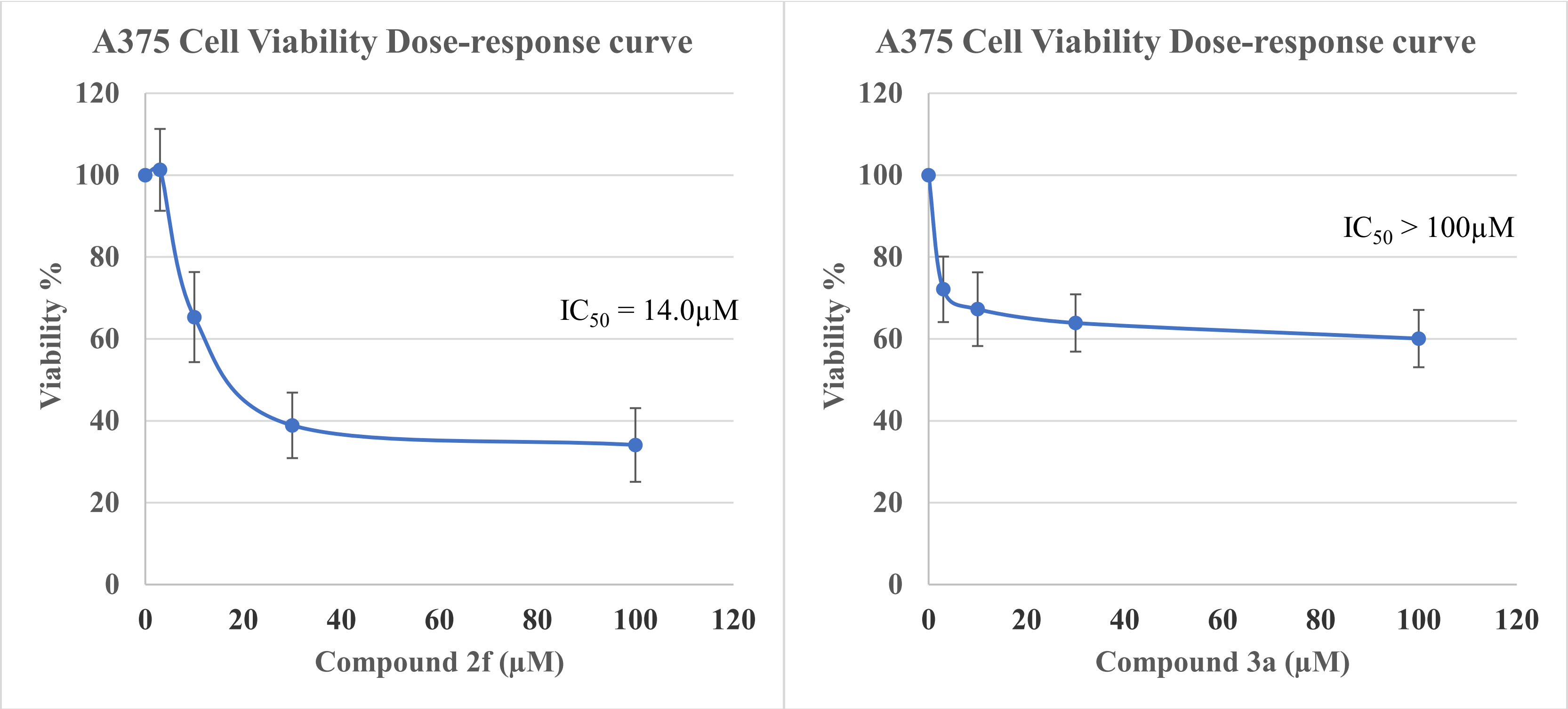

Figure S39. Cell viability Dose-response curve for inhibition of A375 cells. Data are given as mean ± SEM of n= 3 independent experiments, shown as percentage of control, DMSO (= 100%).

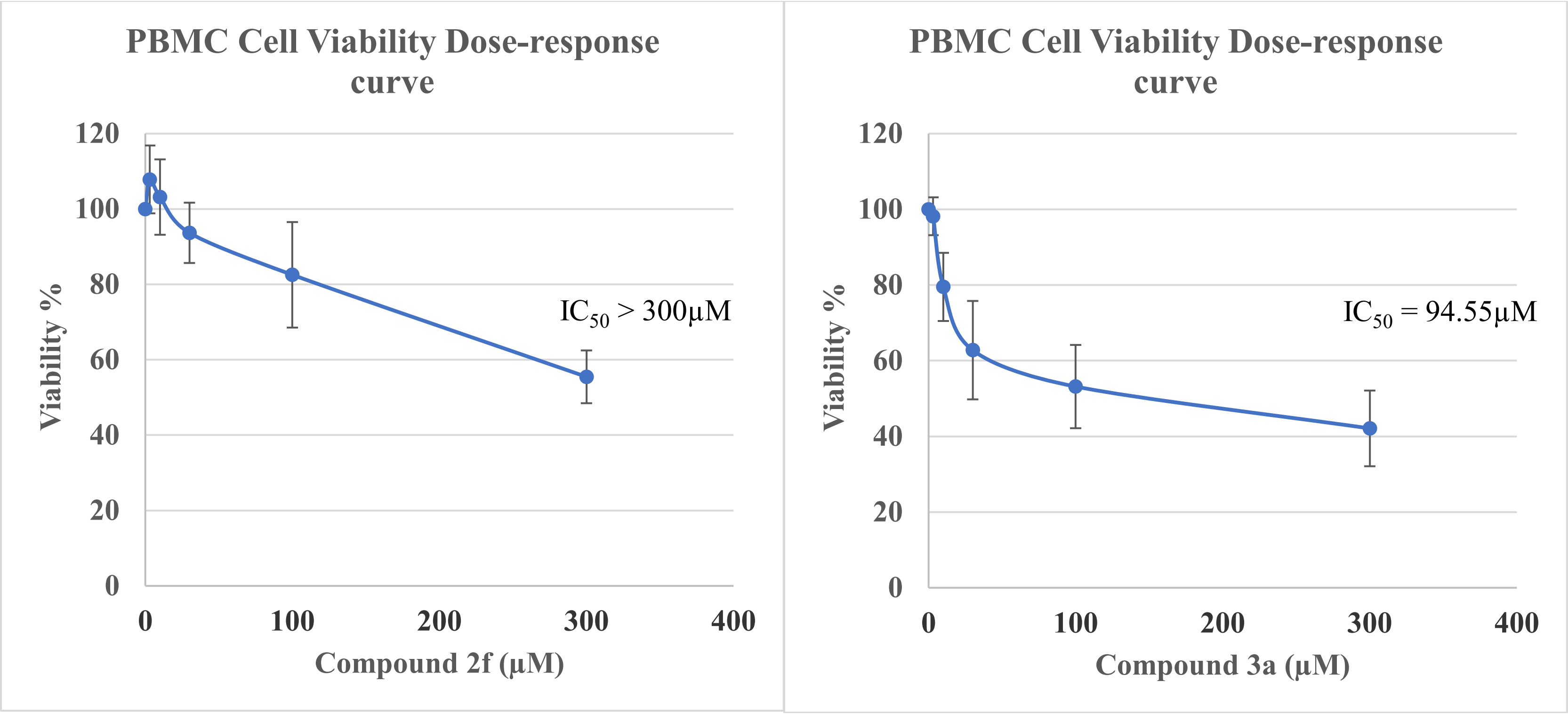

Figure S40. Cell viability Dose-response curve for inhibition of PBMC cells. Data are given as mean ± SEM of n= 3 independent experiments, shown as percentage of control, DMSO (= 100%).

Seed – **Seed +** **3a (30µM)** **2f (30µM)**

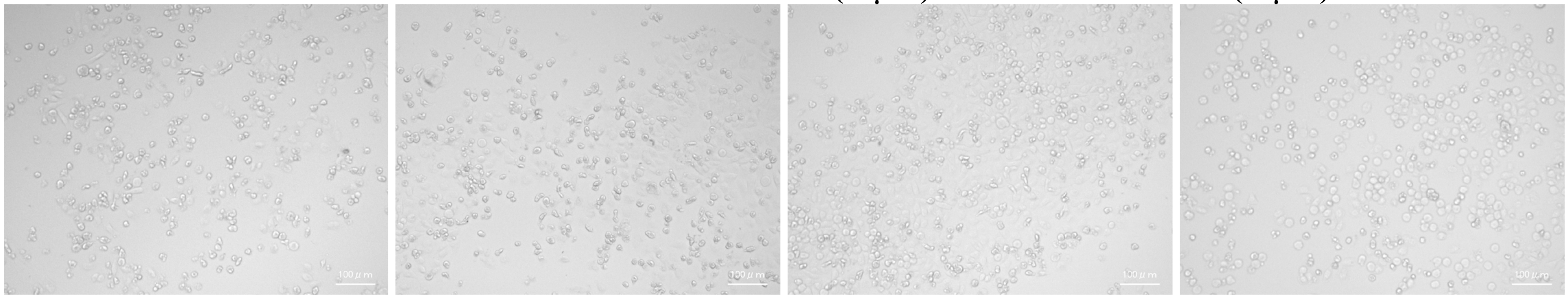

Figure S41. Effects of different concentrations of seed positive (seed +), **2f (30µM)** and **3a (30µM)** on Hela cell morphology photographed under an inverted microscope 24 hours after treatment compared to untreated Hela cells, seed negative (seed -).

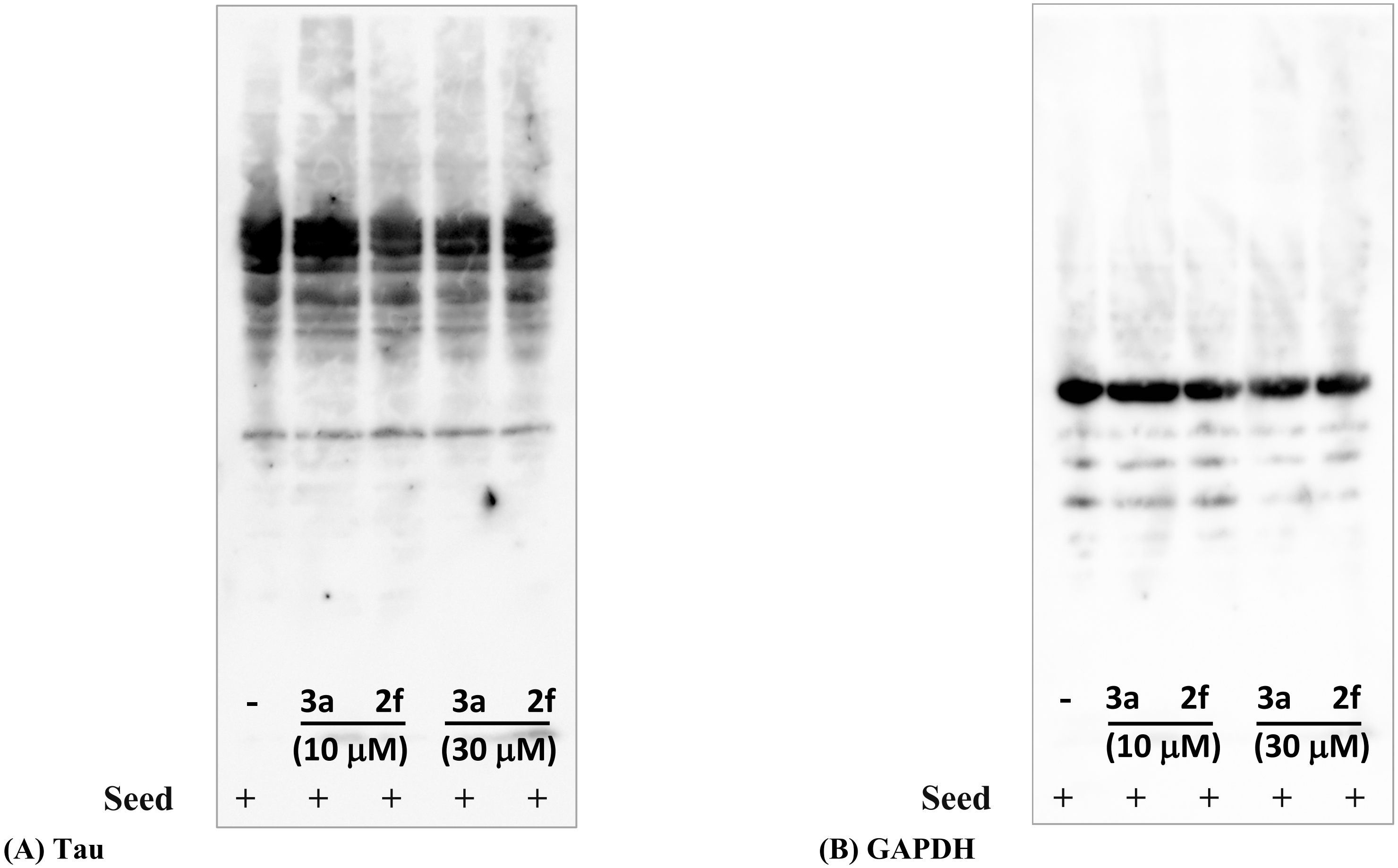

Figure S42. The whole gel of western blot analysis showing the effect of different concentrations of **3a (10μM)**, **2f (10μM) 3a (30μM)** and **2f (30μM)** on total tau proteins **(A),** using GAPDH as a reference **(B)** after 24 hours treatment compared to Hela cells, seed positive (seed +). The same membrane stained with anti-Tau in **(A)** and anti-GAPDH in **(B)**.
